# Research on the severity of symptoms in children with ASD based on integrated machine learning and structural equation modeling: age-specific predictive features and mediation effect path analysis

**DOI:** 10.3389/fped.2026.1841816

**Published:** 2026-06-17

**Authors:** Shimei Lu, Liangqiong Deng, Daoqing Gong, Xinyi Jia, Xiya Lang, Lianghui Diao, Liqin Ma, Pingjing Wen

**Affiliations:** 1 Department of Preventive Medicine, Guangxi University of Chinese Medicine, Nanning, China; 2 Department of Children Healthcare, Liuzhou Hospital of Guangzhou Women and Children's Medical Center, Liuzhou, Guangxi, China

**Keywords:** age at first diagnosis, autism spectrum disorder, developmental level, machine learning, physical development, structural equation modeling, symptom severity

## Abstract

**Background:**

The correlation between age at first diagnosis and symptom severity in autism spectrum disorder (ASD) remains inconsistent in existing research, and the age-specific mechanisms through which developmental level and physical development mediate this relationship remain to be elucidated.

**Methods:**

A total of 608 children with ASD aged 24∼71 months at first diagnosis were recruited and stratified into a low-age group (24∼47 months) and a high-age group (48∼71 months). The Gesell Developmental Schedule (GDS) was utilized to assess developmental level, the Childhood Autism Rating Scale (CARS) to evaluate symptom severity, and height-for-age Z-score (HAZ), weight-for-age Z-score (WAZ), and body mass index-for-age Z-score (BAZ) were calculated to assess physical development. Integrated machine learning was applied to construct classification models for distinguishing severe from non-severe ASD and to identify age-specific predictive features, and structural equation modeling (SEM) was used to analyze the mediation effects and path mechanisms.

**Results:**

Machine learning revealed language as the core predictive feature for the low-age group; whereas in the high-age group, the predictive importance of personal-social, fine motor, gross motor, HAZ, and WAZ increased significantly, with a relative decline in the predictive importance of language. SEM showed a masking effect in the total sample: age at first diagnosis had a positive total indirect effect on symptom severity via developmental level and physical development, but a negative direct effect that offset the total indirect effect, resulting in a non-significant total effect. The mediating role of developmental level was significant, and the effect of developmental level on symptom severity was more pronounced in the low-age group.

**Conclusion:**

This study identifies age-specific predictive features—language as the core predictive feature in the low-age group, whereas in the high-age group, the importance of personal-social, motor, and physical development increases while that of language declines. It also reveals a masking effect of age at first diagnosis on ASD symptom severity: a negative direct effect offset by a positive indirect effect via developmental level and physical development, resulting in a non-significant total effect. These findings provide a preliminary reference for age-stratified intervention in children with ASD.

## 1 Introduction

Autism Spectrum Disorder (ASD) is a neurodevelopmental disorder that manifests in early childhood and is characterized by varying degrees of social interaction impairments, language impairments, restricted interests, and stereotyped behaviors. It severely impairs the social functioning and quality of life of affected children. It is one of the most significant disorders causing mental disability in children, thereby imposing a substantial disease burden on society (1). Currently, the global prevalence of ASD is 1%, with an increasing trend year by year (2). Symptom severity, as a core indicator for the clinical management of ASD, directly determines the priority of intervention strategies and is closely associated with children's social integration, academic performance, and independent living abilities in adulthood (3). Therefore, identifying the key influencing factors and intrinsic mechanisms of symptom severity holds significant clinical value for constructing a precise and efficient intervention system. ASD can lead to various functional disorders or impairments, and poor dietary behaviors and limited physical activity make children with ASD more prone to malnutrition or overnutrition (4). Existing research has found that the prevalence of growth retardation in children aged 2 to 5 with ASD is 4.3%, and the prevalence of wasting is 6.3%. Furthermore, children with severe ASD are more prone to wasting (5). Studies have also shown that the older the age at first diagnosis, the more severe the deviation in physical development (6). This indicates a close relationship between physical development and the age at first diagnosis of ASD, as well as symptom severity.

Li et al. (7) found that the age of first diagnosis was negatively correlated with the developmental quotients of locomotor, hearing and language, eye and hand coordination, performance, and practical reasoning. Studies have also reported that age at first diagnosis is negatively correlated with developmental quotients in adaptive behavior, gross motor, fine motor, and personal-social (6, 8). The severity of ASD symptoms was significantly negatively correlated with the developmental quotients in adaptive behavior, gross motor, fine motor, language, and personal-social (8)**.** This indicates a close relationship between developmental level and the age at first diagnosis of ASD, as well as symptom severity.

While the preceding evidence establishes that both developmental level and physical development are individually correlated with age at first diagnosis and symptom severity, the direct relationship between age at first diagnosis and symptom severity remains controversial. Some studies found that older age at first diagnosis is associated with less severe symptoms (6, 7). However, one study found that the older the age at first diagnosis, the more severe the symptoms (8). This contradiction implies that the relationship is complex and may not be a direct linear one. Instead, age at first diagnosis may indirectly affect symptom severity by influencing children's neurodevelopment and physical health. Developmental level and physical development, as important indicators reflecting neurodevelopment and physical health in children (9), warrant further in-depth exploration regarding whether they mediate the relationship between age at first diagnosis and symptom severity, and whether this mediating role changes dynamically with age.

Previous studies have mostly analyzed the impact of the language and motor sectors on the severity of ASD symptoms in isolation, failing to systematically integrate multidimensional structures, quantify their overall mediating pathways, or explore differences in mediating pathways across age groups (10, 11). Farmer et al. (12) demonstrated, through structural equation modeling (SEM) analyses, that latent variable analysis is methodologically valid for investigating the multidimensional construct of adaptive behavior. To clarify these complex relationships, this study first applies SEM to examine the mediating pathways through which age at first diagnosis, developmental level, and physical development affect symptom severity in children with ASD. Given that machine learning classification approaches have been shown to have good discriminative ability in stratifying clinical severity levels (13), this study further constructs classification prediction models for severe and non-severe ASD using integrated machine learning algorithms, and selects age-specific key predictive features.

## 2 Methods

### 2.1 Participants

A total of 608 children aged 24 to 71 months, who were initially diagnosed with ASD at the Children's Healthcare Department of Liuzhou Hospital, Guangzhou Women's and Children's Medical Centre, between January 2022 and December 2023, were selected as study subjects. Children aged 24 to 47 months were classified as the low-age group, and those aged 48 to 71 months as the high-age group. The 48-month cut-off was selected based on developmental considerations. From a developmental perspective, 48 months marks a pivotal transition in ASD trajectories: core deficits accelerate in manifestation between 2 and 4 years, whereas between 4 and 6 years, differences across developmental domains become more pronounced and increasingly differentiated, signifying a qualitative shift in developmental stage (14). Moreover, language developmental quotient tends to plateau after 48 months, further supporting this time point as a critical developmental juncture (15). Inclusion criteria: The children met the diagnostic criteria for ASD as outlined in the Diagnostic and Statistical Manual of Mental Disorders, Fifth Edition (DSM-5) and were diagnosed by senior doctors with many years of experience in developmental-behavioral pediatrics and rich clinical experience. Additionally, they had not received professional and systematic rehabilitation training. Exclusion criteria: Children with Rett syndrome, trisomy 21, fragile X syndrome, genetic metabolic diseases, organic neuropsychiatric disorders, other disintegrative mental disorders, congenital deafness, visual impairment, etc., were excluded. The children's guardians were informed, agreed to participate in this study, and signed the informed consent form. The Ethics Committee of Liuzhou Hospital approved this study, Guangzhou Women's and Children's Medical Center (NO: 2024-213).

#### 2.1.1 Assessment tools

Physical measurements were conducted by trained and qualified specialist nurses from the Department of Children's Healthcare in our hospital. The same model of infant horizontal measuring bed (<3 years old) or wall-mounted human body meter (≥3 years old) was used for measurement. Body weight and length/height were recorded in kilograms (kg) and centimeters (cm), respectively, both rounded to one decimal place. Before the measurement, children removed their shoes, hats, and diapers and put on lightweight clothing.

Physical development assessment: The measured values were converted into Z-scores for weight and length/height, based on age and sex, to evaluate physical development status. A weight-for-age Z-score (WAZ) below −2 indicates underweight, reflecting acute or chronic malnutrition. A height-for-age Z-score (HAZ) below −2 indicates stunting, reflecting chronic malnutrition. A body mass index-for-age Z-score (BAZ) below −2 indicates wasting, reflecting recent or acute malnutrition (16).

ASD symptom assessment: ASD symptom severity was assessed with the Childhood Autism Rating Scale (CARS). This scale comprises 15 items, each rated on a 1 to 4 scale, with a total score of 60 points. A total score below 30 indicates non-ASD; a score of 30 to 36 with fewer than 5 items with a score below 3 indicates mild-to-moderate ASD; a score of 37 to 60 with at least 5 items with a score above 3 indicates severe ASD. The Cronbach's alpha coefficient for this scale was 0.9, demonstrating good reliability (17).

Developmental level assessment: Intellectual and behavioral development was assessed using the Gesell Developmental Schedule (GDS) (18). The GDS covers five sectors, including adaptive behavior, gross motor, fine motor, language, and personal-social. The evaluation results are presented as a developmental quotient (DQ);DQ ≥86 indicates normal,76–85 indicates borderline, 55–75 indicates mild developmental delay, 40–54 indicates moderate developmental delay, 25–39 indicates severe developmental delay, and <25 indicates extremely severe developmental delay (19).

#### 2.1.2 Quality control

Physical measurements were conducted using standard methods, with instruments calibrated before each measurement and quality supervision provided by on-site inspectors. All professional evaluators possessed evaluation qualifications. The CARS scale assessment was conducted by professionals based on observations of children's behavior and interviews with parents. The GDS scale assessment was conducted strictly in accordance with operational procedures, using a unified set of administration instructions. Data were entered and checked by two people, and consistency checks were performed.

### 2.2 Statistical methods

Statistical analysis was performed using SPSS 26.0. The Shapiro–Wilk test was used to assess normality. For normally distributed quantitative data, results were expressed as mean ± SD, and group comparisons were performed using the independent samples t-test (or Satterthwaite's t-test for heterogeneous variances). For non-normally distributed data, results were expressed as median (P25, P75), and the Mann–Whitney U test was used for group comparisons. For correlation analysis, Pearson's correlation would have been used for normally distributed data; however, given that most continuous variables in this study were not normally distributed, Spearman's rank correlation coefficient was applied instead. Differences were deemed statistically significant when *P* < 0.050.

### 2.3 Construction and evaluation of an integrated machine learning classification prediction model

#### 2.3.1 Algorithm selection and feature processing

To construct a classification prediction model for the severity of ASD symptoms to assist clinical decision-making, the continuous CARS scores were dichotomized based on their clinical diagnostic threshold (CARS scores ≥37 for severe, CARS scores <37 for non-severe). Predictive variables included age at first diagnosis, HAZ, WAZ, BAZ, adaptive behavior, gross motor, fine motor, language, and personal-social. Categorical variables were encoded using one-hot encoding, and continuous predictive variables were standardized using Z-score normalization to eliminate dimensional differences. The selected machine learning algorithms included Random Forest (RF), Gradient Boosting (GBoost), Support Vector Machine (SVM), Logistic Regression (LR), and K-Nearest Neighbors (KNN). These algorithms were chosen based on their complementary advantages: RF has good anti-overfitting ability and feature importance evaluation function; GBoost can effectively capture complex nonlinear relationships through iterative optimization of weak learners; SVM has excellent classification performance in high-dimensional space; LR model is simple and highly interpretable; KNN is suitable for local pattern recognition.

#### 2.3.2 Data preprocessing and enhancement

The total sample was randomly split into a training set and a test set at a 7:3 ratio, with a random seed of 42. The low-age group was split into a training set and a test set at an 8:2 ratio, with a random seed of 30; the high-age group was split into a training set and a test set at a 7:3 ratio, with a random seed of 42. The training set was used for model training and parameter optimization, while the test set served as an independent validation set to evaluate the final model performance. To address potential class imbalance in the training set, the study employs the Synthetic Minority Over-sampling Technique (SMOTE) for data augmentation to improve the model's ability to recognize minority classes.

#### 2.3.3 Parameter optimization and construction of integrated machine learning models

GridSearchCV combined with 5-fold stratified cross-validation was employed to tune the parameters of each algorithm. To avoid overfitting and further enhance predictive performance, a soft voting ensemble model was constructed. The top four single models were selected based on their cross-validation performance, and a weighted soft voting classifier was constructed using their cross-validation accuracies as weights.

#### 2.3.4 Model performance evaluation

The model performance was evaluated on the independent test set using the following metrics: Accuracy, Precision, Recall, F1-Score, Balanced Accuracy, and Area Under the Receiver Operating Characteristic Curve (AUC-ROC). For the AUC-ROC, a value between 0.7 and 0.8 was considered acceptable, 0.8 to 0.9 excellent, and greater than 0.9 outstanding (20). Additionally, the optimal parameter combinations for each model and the cross-validation accuracy were recorded.

#### 2.3.5 Feature importance ranking

To identify the core predictive features that distinguish severe from non-severe ASD, a multi-method feature importance evaluation framework based on six machine learning models was adopted. Specifically, it included the Gini importance of RF and GBoost, the absolute values of LR coefficients, the permutation importance of SVM and KNN, and an integrated feature importance analysis weighted by model performance. All feature importance values were normalized across models and aggregated by ranking to identify stable and consistent core predictive features. Model training and evaluation were performed using Python (version 3.13.5) and Scikit-learn (version 1.6.1).

### 2.4 Construction and validation of SEM

To test the mediating hypothesis that different ages at first diagnosis indirectly affect the severity of ASD symptoms through developmental level and physical development, this study constructed a SEM in R (version 4.3.1) using the lavaan package for analysis.

#### 2.4.1 Model setting

Using age at first diagnosis as the independent variable and ASD symptom severity (CARS scores) as the dependent variable, the latent variables were developmental level (adaptive behavior, gross motor, fine motor, language, and personal-social) and physical development (HAZ and WAZ). Initially, BAZ was included in the model, but its factor load was not significant (*P* > 0.05), and it exhibited a strong residual correlation with WAZ (r = 0.585, *P* < 0.001). Including BAZ would have introduced local dependence and potentially biased the latent variable estimation. Retaining only HAZ and WAZ yields a more parsimonious and statistically sound measurement model while still capturing the essential dimensions of physical development (height and weight). When the factor loading of a measurement indicator is non-significant, removing such an indicator to improve model parsimony is standard SEM practice (21). When two indicators share variance beyond that explained by the latent variable, specifying a correlated residual to account for this shared variance is also an established model modification strategy (22).

#### 2.4.2 Model fitting and evaluation

The maximum likelihood estimation method was employed for parameter estimation. Standard errors and confidence intervals for the parameter estimates were calculated using the Bootstrap method with 5,000 resamples. Results were considered statistically significant if their 95% confidence intervals excluded 0 and the associated *p*-value was less than 0.05. The evaluation of the measurement model generally adhered to the following criteria: a standardized factor loading of greater than 0.70 was considered ideal, while a value of greater than 0.50 was deemed acceptable. A range of 0.30 to 0.50 was regarded as marginally acceptable (23). The model's goodness-of-fit was assessed using the following indicators: Comparative Fit Index (CFI), Tucker–Lewis Index (TLI), Root Mean Square Error of Approximation (RMSEA), and Standardized Root Mean Square Residual (SRMR). An acceptable fit was defined by CFI and TLI values greater than 0.90, and RMSEA and SRMR values less than 0.08. A good fit was characterized by CFI and TLI values greater than 0.95 and RMSEA and SRMR values less than 0.06 (12). SEM was fit separately for the total sample and the low-age group. Given the relatively small sample size in the high-age group, to ensure statistical validity and model stability, the total sample was the main analysis object, with the low-age group serving as a sensitivity test. The model results for the high-age group were used only for reference and were not subjected to in-depth path comparisons or discussion.

## 3 Results

### 3.1 Clinical features

A total of 608 children with ASD were enrolled in this study, including 461 in the low-age group and 147 in the high-age group. There were no statistically significant differences in gender regarding the medians of adaptive behavior, gross motor, fine motor, language, personal-social DQ, as well as HAZ, WAZ, BAZ, and CARS scores between the two groups (*P* > 0.05), indicating that the two groups were balanced and comparable in terms of gender. The medians of adaptive behavior, gross motor, fine motor, personal-social DQ, HAZ, and WAZ of ASD children in the low-age group were significantly higher than those in the high-age group (*P* < 0.05). At the same time, there were no statistically significant differences in language DQ, CARS scores, and BAZ between the two groups (*P* > 0.05). In addition, the medians of adaptive behavior, gross motor, fine motor, language, personal-social DQ, HAZ, and WAZ of ASD children in the severe group were significantly lower than those in the non-severe group (*P* < 0.05) (Table 1).

**Table 1:** Comparison of developmental levels and physical development in children with ASD by age at first diagnosis, gender, and symptom severity.

Group	*N*	HAZ	WAZ	BAZ	CARS scores	GDS				
						Adaptive behavior	Gross motor	Fine motor	Language	Personal-social
Age										
Low-age group	461	−0.35 (−1.22,0.65)	−0.17 (−0.83,0.79)	−0.14 (−0.535,0.81)	37 (34,41)	60 (50,69)	79 (71,86)	68 (57,77)	35 (28,44)	57 (48,64)
High-age group	147	−1.16 (−2.25,−0.3)	−0.75 (−1.54,0.05)	−0.11 (−0.57,0.81)	37 (34,41)	54 (45,61)	73 (62,79)	64 (51,72)	37 (27,46)	54 (45,61)
Z score		−6.533	−6.148	−0.009	−1.751	−4.529	−5.424	−3.537	−0.105	−2.403
*P* value		＜0.001	＜0.001	0.993	0.080	＜0.001	＜0.001	＜0.001	0.916	0.016
Symptom severity of ASD										
Non-severe	275	−0.41 (−1.28,0.72)	−0.18 (−0.85,0.65)	−0.15 (−0.48,0.83)	34 (31,35)	65 (50,71)	79 (70,86)	69 (59,80)	42 (35,51)	60 (52,68)
Severe	333	−0.62 (−1.49,−0.17)	−0.38 (−1.14,0.51)	−0.11 (−0.59,0.75)	40 (38,44)	54 (45,65)	77 (66.5,84)	63 (52,73)	31 (25,38.5)	53 (45,59)
Z score		−2.263	−2.251	−0.759	−21.277	−6.565	−2.227	−5.144	−10.285	−7.746
*P* value		0.024	0.024	0.448	＜0.001	＜0.001	0.026	＜0.001	＜0.001	＜0.001
Gender										
Male		−0.54 (−1.43,0.49)	−0.27 (−1.0,0.62)	0.14 (−0.51,0.84)	37 (34,41)	59 (49,67)	77 (67.5,85)	66 (56,76)	36 (28,46)	56 (48,64)
Female		−0.61 (−1.38,0.16)	−0.37 (−1.15,0.45)	−0.07 (−0.64,0.64)	38 (34,41)	58 (46,69)	78 (67,86)	66 (54,74)	36 (27,42)	57 (48,64)
Z score		−0.696	−1.197	−1.511	−0.479	−0.215	−0.217	−0.504	−0.589	−0.085
*P value*		0.486	0.231	0.131	0.632	0.830	0.828	0.615	0.556	0.932

Data are presented as median (P25, P75). HAZ, height-for-age z-score; WAZ, weight-for-age z-score; BAZ, BMI-for-age z-score; GDS, Gesell Developmental Schedules; CARS, Childhood Autism Rating Scale. Group comparisons were performed using the Mann–Whitney U test.

The prevalence of developmental delays in children with ASD in the areas of adaptive behavior, gross motor, fine motor, language, and personal-social was 89.3%, 42.3%, 74.2%, 98.7%, and 94.7%, respectively (Table 2).

**Table 2:** Distribution of GDS developmental levels in children with ASD.

Adaptive behavior			Gross motor			Fine motor			Language			Personal-social		
Normal	Borderline	Developmental delay	Normal	Borderline	Developmental delay	Normal	Borderline	Developmental delay	Normal	Borderline	Developmental delay	Normal	Borderline	Developmental delay
9 (1.5)	56 (9.2)	543 (89.3)	143 (23.5)	208 (34.2)	257 (42.3)	55 (9.0)	102 (16.8)	451 (74.2)	3 (0.5)	5 (0.8)	600 (98.7)	4 (0.7)	28 (4.6)	576(94.7)

### 3.2 Analysis of the correlation between variables

Except for language, the age at first diagnosis was significantly negatively correlated with adaptive behavior, gross motor, fine motor, personal-social DQ, HAZ, and WAZ (*P* < 0.001). The CARS scores were significantly negatively correlated with adaptive behavior, gross motor, fine motor, language, personal-social DQ, HAZ, and WAZ (*P* < 0.05) (Table 3).

**Table 3:** Correlation analysis of age at first diagnosis, CARS scores, developmental level, and physical development in children with ASD.

Variable	Age at first diagnosis	CARS scores	Adaptive behavior	gross motor	Fine motor	Language	Personal-social	HAZ	WAZ	BAZ
Age at first diagnosis	—	0.014	−0.221***	−0.238***	−0.173***	−0.063	−0.180***	−0.275***	−0.265***	−0.010
CARS scores	0.014	—	−0.360***	−0.123***	−0.277***	−0.479***	−0.345***	−0.124**	−0.102*	−0.013

Values shown are Spearman's correlation coefficients. HAZ, height-for-age z-score; WAZ, weight-for-age z-score; BAZ, BMI-for-age z-score; CARS, childhood autism rating scale.

*P < 0.05,**P < 0.01,***P < 0.001.

### 3.3 Model evaluation and machine learning performance

**Figure 1:**
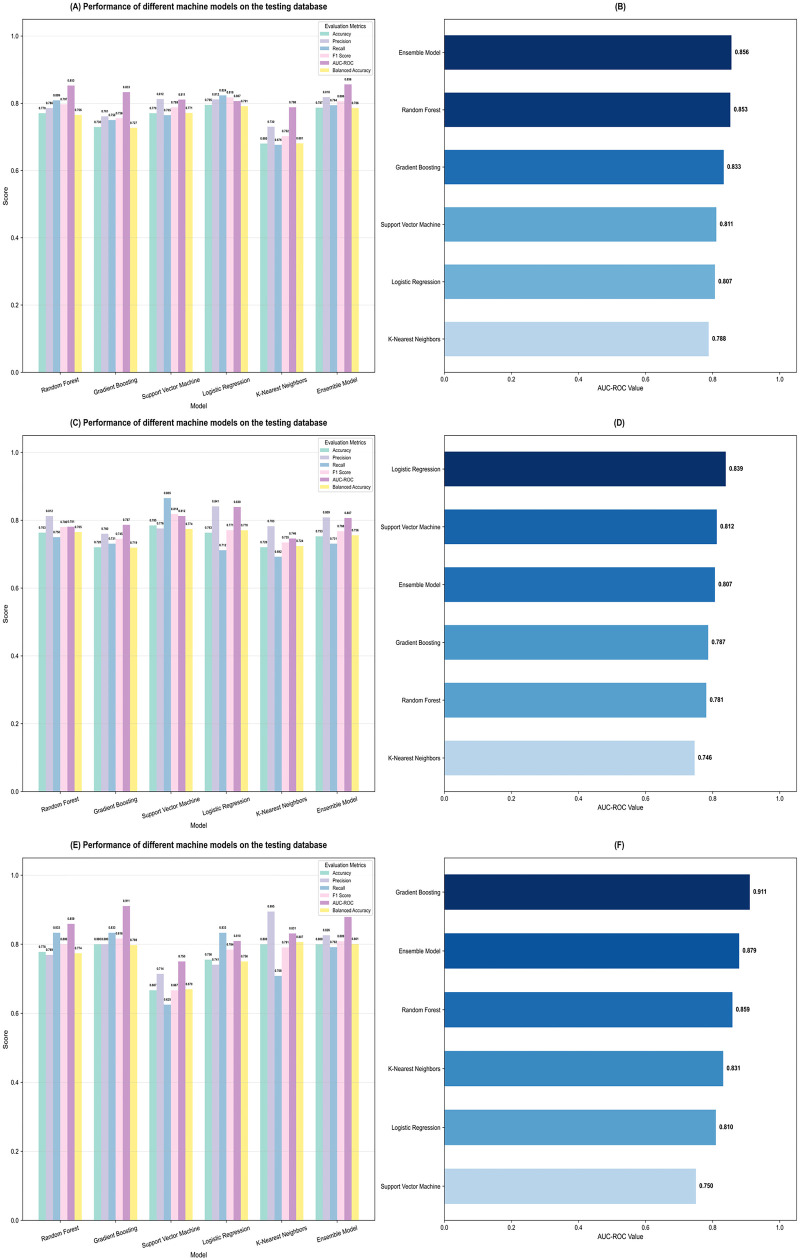
Performance evaluation of machine learning models for distinguishing severe from non-severe ASD in the total sample **(A, B)**, low-age group (24–47 months) **(C, D)**, and high-age group (48–71 months) **(E, F)**. ROC, receiver operating characteristic; AUC, area under the curve; SVM, support vector machine; LR, logistic regression; RF, random forest; GBoost, gradient boosting; KNN, k-nearest neighbors.

In the total sample, the accuracy of the RF, GBoost, SVM, LR, KNN, and ensemble models ranged from 0.680 to 0.795, and the F1 scores ranged from 0.702 to 0.818, indicating robust overall performance across all models. The AUC-ROC values ranged from 0.788 to 0.856 (Figures 1A, B), all exceeding the acceptable threshold.

In the low-age group, the accuracy of the RF, GBoost, SVM, LR, KNN, and ensemble models ranged from 0.720 to 0.785, and the F1 scores ranged from 0.735 to 0.818, again indicating robust performance across all models. The AUC-ROC values ranged from 0.746 to 0.839 (Figures 1C, D), all exceeding the acceptable threshold.

In the high-age group, the accuracy of the RF, GBoost, SVM, LR, KNN, and ensemble models ranged from 0.667 to 0.800, and the F1 scores ranged from 0.667 to 0.816, again indicating robust performance across all models. The AUC-ROC values ranged from 0.750 to 0.911 (Figures 1E, F), all exceeding the acceptable threshold.

### 3.4 Analysis of feature importance ranking

**Figure 2:**
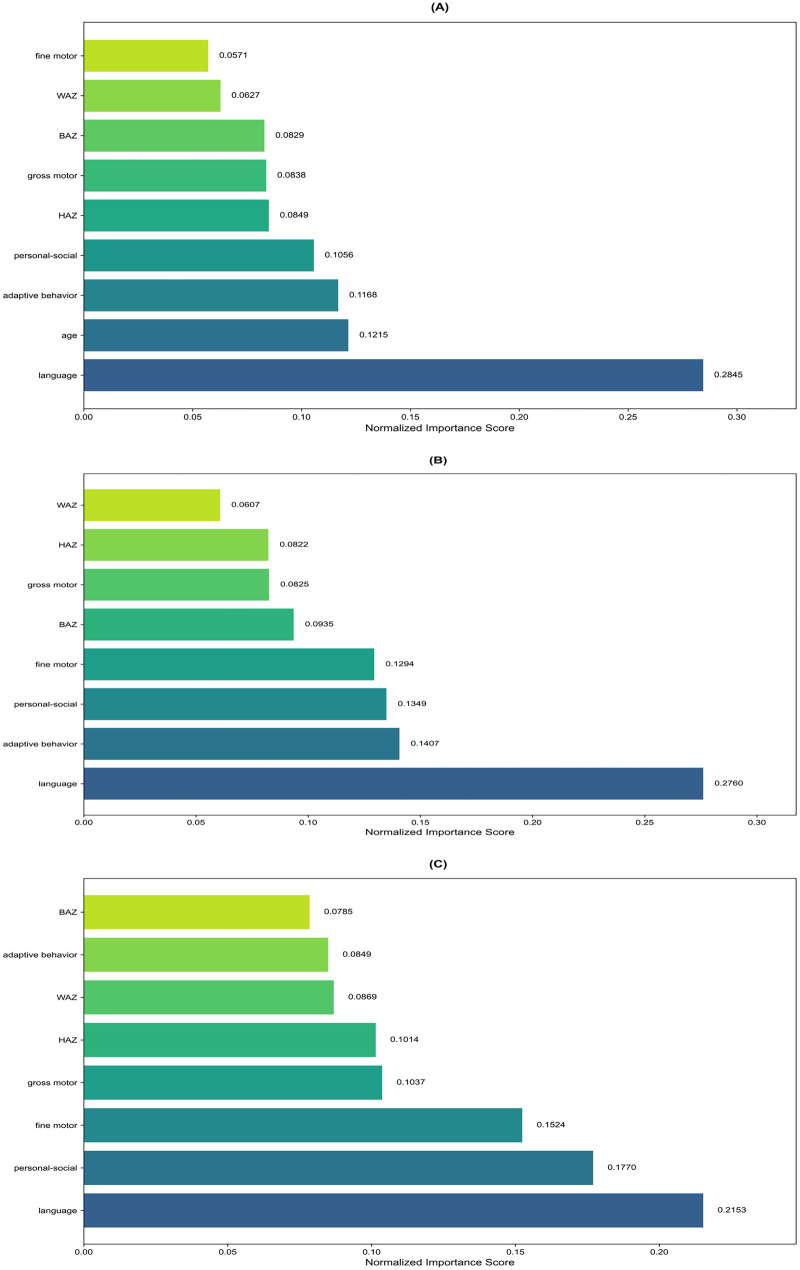
Normalized feature importance scores for distinguishing severe from non-severe ASD in the total sample (A), low-age group (24–47 months) (B) and high-age group (48–71 months) (C). The vertical axis denotes feature names, and the horizontal axis denotes normalized feature importance scores. HAZ, height-for-age Z-score; WAZ, weight-for-age Z-score; BAZ, body mass index-for-age Z-score.

In the total sample, the predictive importance scores of each feature for distinguishing between severe and non-severe ASD were ranked from high to low as follows: language (0.2845), age at first diagnosis (0.1215), adaptive behavior (0.1168), and personal-social(0.1056). The predictive scores for gross motor, fine motor, HAZ, BAZ, and WAZ were relatively low (all < 0.09) (Figure 2A). In the low-age group, language remained the most important predictive feature (0.2760), followed by adaptive behavior (0.1407), personal-social (0.1349), and fine motor (0.1294). The predictive scores for gross motor (0.0825), BAZ (0.0935), HAZ (0.0822), and WAZ (0.0607) were relatively low (Figure 2B). Compared with the low-age group, the high-age group showed a lower score in the language (0.2153 vs. 0.2760), while the score in the personal-social significantly increased (0.1770 vs. 0.1349). The scores for fine motor (0.1524 vs. 0.1294), gross motor (0.1037 vs. 0.0825), HAZ (0.1014 vs. 0.0822), and WAZ (0.0869 vs. 0.0607) all significantly increased (Figure 2C).

### 3.5 SEM

The model-fitting results showed that the CFI, TLI, RMSEA, and SRMR for the total sample were 0.982, 0.968, 0.074, and 0.038, respectively; for the low-age group, they were 0.988, 0.978, 0.060, and 0.036. All fitting indices met the good or acceptable standards, indicating that the model fit the data well. Especially in the low-age group, the RMSEA and SRMR further decreased, suggesting that the model fit better in this age group.

#### 3.5.1 Measurement model

The standardized loadings of all observed variables on their respective latent variables were significant (*P* < 0.001). In the total sample, the loadings for each developmental level dimension ranged from 0.575 to 0.851, and those for each physical development dimension ranged from 0.439 to 0.496. In the low-age group, the loadings for developmental level ranged from 0.433 to 0.789, and the loadings for physical development ranged from 0.501 to 0.605.

#### 3.5.2 Structural model

**Figure 3:**
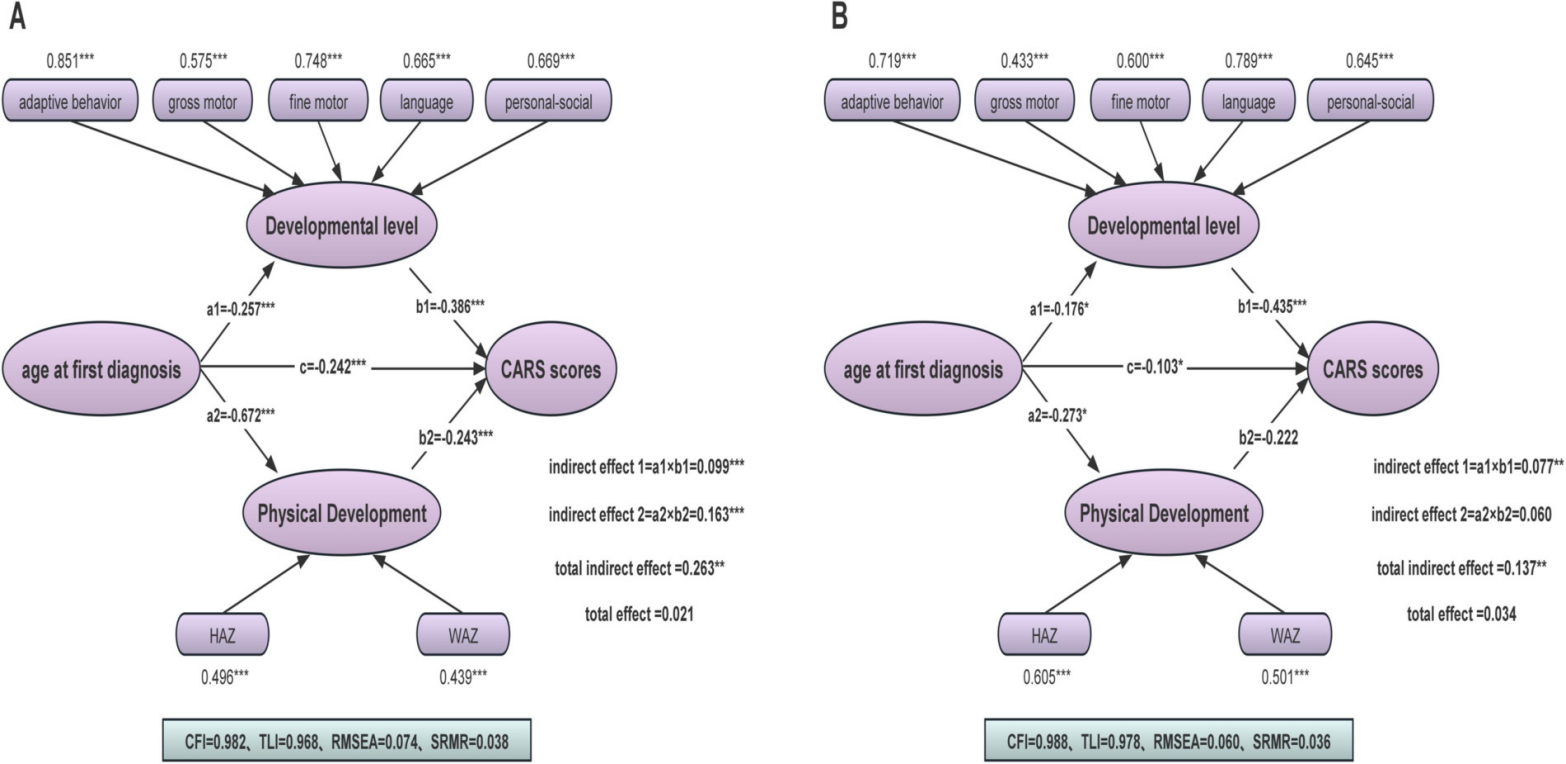
Path coefficient diagrams with standardized coefficients for the total sample **(A)** and low-age group (24–47 months) **(B)** All paths are significant at *P < 0.05, **P < 0.01, ***P < 0.001. CARS, childhood autism rating scale; HAZ, height-for-age Z-score; WAZ, weight-for-age Z-score.

The SEM for the total sample revealed that age at first diagnosis exerted a significant negative effect on both developmental level (*β* = −0.257; 95% CI: −4.574 to −2.214; *P* < 0.001) and physical development (*β* = −0.672; 95% CI: −0.893 to −0.527; *P* < 0.001). Meanwhile, both developmental level (*β* = −0.386; 95% CI: −0.306 to −0.179; *P* < 0.001) and physical development (*β* = −0.243; 95% CI: −2.273 to −1.324; *P* < 0.001) had a significant negative impact on CARS scores. Mediation analysis results indicated that the mediating effect of developmental level on the relationship between age at first diagnosis and CARS scores was significant (*β* = 0.099; 95% CI: 0.504 to 1.169; *P* < 0.001), and that of physical development was also significant (*β* = 0.163; 95% CI: 0.846 to 1.708; *P* < 0.001), with the total indirect effect being 0.263 (95% CI: 1.638 to 2.591; *P* < 0.001). The direct effect of age at first diagnosis on CARS scores was −0.242 (95% CI: −2.573 to −1.375; *P* < 0.001), which was in the opposite direction to the total indirect effect; the two effects offset each other, ultimately resulting in a non-significant total effect (Figure 3A; Table 4).

**Table 4:** Path coefficients of the structural equation model for the total sample.

Variables	B	95% Confidence interval		Z	*P*	*β*
		Lower	Upper			
Measurement model						
Adaptive behavior→developmental level	1.000	1.000	1.000			0.851
Gross motor→developmental level	0.607	0.517	0.699	12.874	<0.001	0.575
Fine motor→developmental level	0.978	0.867	1.084	17.734	<0.001	0.748
Language→developmental level	0.779	0.655	0.925	11.310	<0.001	0.665
Personal-social→developmental level	0.707	0.589	0.845	11.059	<0.001	0.669
HAZ→physical development	1.000	1.000	1.000			0.496
WAZ→physical development	0.662	0.639	0.685	56.628	<0.001	0.439
Structural model						
Age at first diagnosis→developmental level	−3.377	−4.574	−2.214	−5.607	<0.001	−0.257
Age at first diagnosis→physical development	−0.718	−0.893	−0.527	−7.825	<0.001	−0.672
Developmental level→CARS scores	−0.241	−0.306	−0.179	−7.466	<0.001	−0.386
Physical development→CARS scores	−1.868	−2.273	−1.324	−7.882	<0.001	−0.243
Age at first diagnosis→CARS scores	−1.987	−2.573	−1.375	−6.504	<0.001	−0.242
Mediating effect						
Indirect effect 1	0.815	0.504	1.169	4.791	<0.001	0.099
Indirect effect 2	1.341	0.846	1.708	6.226	<0.001	0.163
Total indirect effect	2.156	1.638	2.591	8.673	<0.001	0.263
Direct effect	−1.987	−2.573	−1.375	−6.500	<0.001	−0.242
Total effect	0.169	−0.490	0.859	0.498	0.619	0.021

Indirect effect 1 = age at first diagnosis→developmental level→CARS scores,indirect effect 2 = age at first diagnosis→physical development→CARS scores,total indirect effect = indirect effect 1 + indirect effect 2,direct effect = age at first diagnosis→CARS scores,total effect = total indirect effect + direct effect. B, unstandardized regression coefficient; *β*, standardized regression coefficient; CI, confidence interval; z: z-value; p: *p*-value.

In the low-age group, the path relationships exhibited significant changes: the negative effect of age at first diagnosis on developmental level was attenuated (*β* = −0.176; 95% CI: −6.066 to −0.775; *P* < 0.05), whereas the negative effect of developmental level on CARS scores was enhanced (*β* = −0.435; 95% CI: −0.398 to −0.229; *P* < 0.001). Mediation analysis results showed that the mediating effect of developmental level on the relationship between age at first diagnosis and CARS scores was attenuated (*β* = 0.077; 95% CI: 0.253 to 1.787; *P* < 0.01). The total indirect effect in this subgroup attenuated to 0.137 (95% CI: 0.159 to 2.641; *P* < 0.01). (Figure 3B; Table 5).

**Table 5:** Path coefficients of the structural equation model for the low-age group.

Variables	B	95% Confidence interval		Z	*P*	*β*
		Lower	Upper			
Measurement model						
Adaptive behavior→developmental level	1.000	1.000	1.000			0.719
Gross motor→developmental level	0.517	0.405	0.631	8.905	<0.001	0.433
Fine motor→developmental level	0.931	0.798	1.078	13.011	<0.001	0.600
Language→developmental level	1.116	0.866	1.609	5.932	<0.001	0.789
Personal-social→developmental level	0.789	0.579	1.057	6.748	<0.001	0.645
HAZ→physical development	1.000	1.000	1.000			0.605
WAZ→physical development	0.612	0.280	0.850	4.033	<0.001	0.501
Structural model						
Age at first diagnosis→developmental level	−3.385	−6.066	−0.775	−2.546	0.011	−0.176
Age at first diagnosis→physical development	−0.613	−1.001	0.108	−1.982	0.047	−0.273
Developmental level→CARS scores	−0.309	−0.398	−0.229	−7.306	<0.001	−0.435
Physical development→CARS scores	−1.349	−1.958	1.702	−1.181	0.238	−0.222
Age at first diagnosis→CARS scores	−1.407	−2.475	0.352	−2.001	0.045	−0.103
Mediating effect						
Indirect effect 1	1.046	0.253	1.787	2.680	0.007	0.077
Indirect effect 2	0.827	−0.477	1.431	1.572	0.116	0.060
Total indirect effect	1.874	0.159	2.641	2.921	0.003	0.137
Direct effect	−1.407	−2.475	0.352	−2.000	0.046	−0.103
Total effect	0.466	−0.645	1.577	0.815	0.415	0.034

Indirect effect 1 = age at first diagnosis→developmental level→CARS scores,indirect effect 2 = age at first diagnosis→physical development→CARS scores,total indirect effect = indirect effect 1 + indirect effect 2, direct effect = age at first diagnosis→CARS scores,total effect = total indirect effect + direct effect. B, unstandardized regression coefficient; *β*, standardized regression coefficient; CI, confidence interval; z: z-value; p: *p*-value.

## 4 Discussion

ASD is a highly heterogeneous neurodevelopmental disorder, with clinical manifestations not only reflected in core social and behavioral traits but also frequently accompanied by issues such as developmental delay and physical developmental deviations. As individuals age, the correlation between these issues and symptom severity may change dynamically; therefore, identifying the key factors influencing symptom severity at different ages is crucial for understanding the age-specific manifestations of developmental deviations of ASD and implementing stratified interventions.

Inconsistent conclusions have been reached regarding the association between age at first diagnosis and ASD symptom severity. Through SEM-based mediation effect analysis, the present study systematically revealed, for the first time, the complex pathways underlying this inconsistency. Specifically, in the total sample, age at first diagnosis exerted a significant negative direct effect on ASD symptom severity, which is consistent with the findings that an older age at first diagnosis correlates with milder symptoms (6, 7). However, age at first diagnosis simultaneously exerted a positive indirect effect on symptom severity through two mediating pathways: First, age at first diagnosis had a significant negative effect on developmental level, which in turn exerted a significant negative effect on symptom severity, forming a mediating pathway suggesting that an older age at first diagnosis may be associated with a lower developmental level, which in turn may contribute to more severe symptoms; second, age at first diagnosis had a significant negative effect on physical development, which also exerted a significant negative effect on symptom severity, forming another mediating pathway where an older age at first diagnosis may be linked to lower physical development, which may further contribute to more severe symptoms. The total indirect effect of these two pathways was in the opposite direction to the direct effect, and the two effects offset each other. This ultimately resulted in a non-significant total effect of age at first diagnosis on ASD symptom severity, leading to a statistical masking effect (24). This may explain why there was no significant correlation between age at first diagnosis and CARS scores, while a significant negative correlation was revealed after controlling for developmental level and physical development.

The research results showed that the prevalence of developmental delays in children with ASD in the DQ of adaptive behavior, gross motor, fine motor, language, and personal-social were 89.3%, 42.3%, 74.2%, 98.7%, and 94.7%, respectively, presenting an imbalance with language developmental delay being the most prominent. Pan et al. (25) also found that the proportion of children with ASD who exhibited language developmental delays was 94%, which is similar to the rate of language developmental delay in this study. Language is an important tool for social communication, and children with language developmental delays often exhibit deficits in social interaction. Roseberry et al. (26) suggested that systematic, long-term language training can help children with ASD improve their social communication. Furthermore, adaptive behavior and personal-social developmental delays are also developmental issues that children with ASD face simultaneously. The study found that the rate of adaptive behavior developmental delay is 89.3%. Estes et al. (27) provided longitudinal evidence for this: high-risk infants with ASD exhibited adaptive behavior and functional delays as early as 6 months, and these differences gradually widened with age, further declined in areas such as communication, daily living skills, and social interaction by 24 months. These findings are highly consistent with our results.

In the low-age group, language showed the highest predictive score in the model distinguishing between severe and non-severe ASD, which is consistent with clinical observations that children with ASD generally lag in early language development. Zhao et al. (28) pointed out that the first word appeared between 8 and 14 months in normal children, while it might be delayed until 38 months in ASD, and pragmatic communication disorders were particularly prominent**.** Seol et al. (29) research further confirmed that ASD toddlers exhibited significant impaired receptive language abilities in the early stages, and the younger the age, the more pronounced this defect became. This suggests that the lag in language ability development may form a stable developmental defect pattern in the early stages of ASD. Recently, Hodge et al. (30) also reported that about 25.9% of children who meet the criteria for high-risk ASD exhibited severe impairment in language function, such as an inability to use nouns and verb phrases, on average at 21.49 months. The above research results collectively support this model's findings: language is the most predictive indicator for distinguishing between severe and non-severe symptoms in the low-age group.

Compared to the low-age group, in the high-age group, distinguishing between severe and non-severe ASD, scores in language decreased, whereas scores in personal-social increased significantly. Additionally, scores in fine motor and gross motor also demonstrated significant improvement. These changes suggest that, as age increases, the roles of social and motor abilities in distinguishing between severe and non-severe ASD become progressively more prominent, while the influence of language remains relatively weak. Zhao et al. (28) found that even if some ASD children's semantic and grammatical abilities reached normal levels, they still had persistent difficulties in understanding and using language in social interactions. This problem could persist into adulthood, indicating that as age increases, the actual needs and deficiencies of personal-social became more prominent. Meanwhile, Provost et al. (31) showed that ASD toddlers often exhibited coordinated developmental delays in gross and fine motor abilities. After entering the preschool age, motor tasks became more complex, requiring greater planning and imitation, making motor deficits more apparent. In addition, Xiong et al. (11) research also demonstrated that among ASD children under 4 years old, receptive language items such as “point out the action in the picture” and “point out a picture,” as well as expressive language items such as “express one's needs in language,” were the most significant language milestones affecting core symptoms; while among children aged 4 and above, social interaction-related language items such as “calling peers by name” and “saying ‘no'” became key indicators for predicting core symptoms. This finding is highly consistent with the results of this study. Furthermore, Wang et al. (10) research on ASD children aged 2–6 also revealed an age-dynamic pattern: among ASD children with normal adaptive behavior abilities, the degree to which gross motor abilities lag behind adaptive behavior abilities intensifies with age; while among children with adaptive behavior at a critical level, the relative advantage of gross motor abilities weakens after the age of 4. This finding corroborates the phenomenon revealed by this study, based on machine learning models, in which the weights of core predictive features shift across age groups, jointly indicating that the influence of different developmental ability areas on distinguishing between severe and non-severe ASD in children is age-specific.

The SEM path results showed that in the total sample, age at first diagnosis had a significant negative effect on developmental level, which in turn negatively affected ASD symptom severity, establishing developmental level as a significant mediator. This suggests that a later diagnosis age is associated with lower developmental level and more severe symptoms. Notably, in the low-age group, the negative effect of age at first diagnosis on developmental level was weakened, whereas the negative effect of developmental level on symptom severity was enhanced, with an attenuated mediating effect. Yet, this finding underscores the central role of developmental level in the low-age group. The age range of 2 to 4 years is considered a sensitive period for the formation and remodeling of neural circuits in the brain. During this period, synaptic formation, pruning, and neural circuit functional differentiation are particularly active and are easily influenced by internal and external environments. If ASD continues to experience developmental delays due to diagnostic delays or lack of intervention during this period, their neural circuits may not be fully optimized within the critical window, leading to biases in neural network development, manifested as more severe core symptoms of ASD (32). Therefore, the role of developmental level in the early stages may reflect deviations and irreversibility in neural developmental pathways due to the lack of effective intervention during the critical period of brain plasticity. From a neurophysiological perspective, this mediating pathway may be closely related to abnormalities in sensorimotor processing. Ebishima et al. (33) reported that ASD was characterized by prolonged latency in the auditory startle reflex (ASR), which was significantly correlated with lower levels of adaptive behavior. Abnormal ASR reflects deficits in ASD individuals' ability to receive, integrate, and respond to sensory information, which may lead to delayed processing of language input and difficulty in recognizing social cues, thus forming a cascade effect of “abnormal sensory processing → developmental delay → symptom aggravation”. Therefore, delays in age at first diagnosis may lead to children not receiving timely intervention to optimize neural circuit remodeling, resulting in more severe symptoms. This mechanism provides a neurophysiological explanation for the “age at first diagnosis → developmental level → symptom severity” pathway revealed in this study.

This study found that the DQ in adaptive behavior, gross motor, fine motor, and personal-social was significantly lower in the high-age group compared to the low-age group. Wang et al. (8) also showed that the DQ in adaptive behavior, gross motor, fine motor, and personal-social was significantly lower in children aged 3 and above than in those under 3 years old. Li et al. (15) also reported that the locomotor quotient decreased significantly with increasing age at first diagnosis. These conclusions are consistent with the model's finding that the age at first diagnosis has a significant negative effect on developmental level; Spearman's correlation analysis revealed significant negative correlations between the CARS scores and the DQ in adaptive behavior, gross motor, fine motor, language, and personal-social. The longitudinal study by Pino et al. (34) also showed that developmental levels in Personal-Social, Hearing and Language, and Practical Reasoning were significantly negatively correlated with the severity of ASD symptoms. These conclusions are consistent with the model's finding that developmental level has a significant negative effect on the severity of ASD symptoms; more compellingly, the mediating role of developmental level between age at first diagnosis and symptom severity revealed in this study has been validated in early intervention practice. Colombi et al. (35) reported a case of an infant who showed early behavioral signs of ASD at 6 months of age. Through parent-mediated intervention based on the Early Start Denver Model (ESDM), the infant's developmental level continued to improve throughout the intervention, and by 32 months of age, the infant no longer met the diagnostic criteria for ASD. This case demonstrates that implementing intervention during the earliest developmental window can effectively enhance developmental level, thereby improving the trajectory of ASD symptom severity and, to some extent, reversing the developmental path.

The results of this study showed that the HAZ and WAZ of the high-age group were significantly lower than those of the low-age group, indicating that physical development deviation is more prominent in older children with ASD. This overall trend is further elucidated in SEM. Between 24 and 71 months of age, age at first diagnosis had a significant negative effect on physical development, which in turn negatively affected ASD symptom severity, establishing physical development as a significant mediator. This suggests that a later diagnosis age is associated with lower physical development and more severe symptoms. Notably, in the low-age group, the negative effect of age at first diagnosis on physical development was attenuated, and the negative effect of physical development on symptom severity was no longer significant, with a non-significant mediating effect. This finding indicates an age-specific association between physical development and ASD symptom severity. Given the unique characteristics of the developmental window, developmental level exerts a more pronounced impact on ASD symptom severity in early stages, while the mediating pathway of physical development may not be fully established at this stage, and its effects may require a longer accumulation period to manifest. Xie et al. (36) reported that individuals with ASD frequently exhibited behavioral traits including low levels of physical activity and atypical dietary patterns (e.g., a predilection for high-calorie foods), which were recognized as potential risk factors for nutritional imbalance and physical development deviation. Sammels et al. (37) also identified that the prevalence of obesity in children with ASD increased with age and was positively correlated with ASD symptom severity. The present study also reveals more prominent deviation in physical development within the high-age group, where physical development is significantly positively associated with ASD symptom severity, consistent with previous literature. Furthermore, results from the machine learning models demonstrated a marked increase in the predictive importance of HAZ and WAZ for distinguishing between severe and non-severe ASD in the high-age group, methodologically confirming that physical development exerts a more prominent predictive effect on symptom severity, specifically in the high-age group.

## 5 Conclusion

Using machine learning, the present study identifies age-specific predictive features for distinguishing severe and non-severe ASD: language is the core predictive feature in the low-age group, whereas in the high-age group, the predictive importance of personal-social, fine motor, gross motor, HAZ, and WAZ increases significantly, and that of language decreases relatively. Using SEM, this study identifies for the first time a masking effect of age at first diagnosis on ASD symptom severity. Specifically, age at first diagnosis exerts a negative direct effect on symptom severity, yet it simultaneously exerts a positive indirect effect via developmental level and physical development. The offsetting of these two effects results in a non-significant total effect, thereby explaining the inconsistencies in previous research conclusions. The mediating role of developmental level was significant, and the effect of developmental level on symptom severity was more prominent in the low-age group. These findings provide a preliminary reference for age-stratified intervention in children with ASD.

The study is limited to a single center and a cross-sectional design, which may introduce selection bias, limit the generalizability of the findings, and preclude causal inference. The relatively small sample size of the high-age group (*n* = 147) limits the robustness of the predictive models in this subgroup; the machine learning findings in this subgroup should be considered exploratory. The predictive models lacked external validation, and future multicenter, prospective studies with larger cohorts are warranted to include ASD children from diverse geographic regions and with different clinical characteristics to cross-validate these age-specific features.

## Data Availability

The original contributions presented in the study are included in the article/Supplementary Material, further inquiries can be directed to the corresponding authors.
