# Supplementary material for: Research on the severity of symptoms in children with ASD based on integrated machine learning and structural equation modeling: age-specific predictive features and mediation effect path analysis
Source: Front Pediatr. 2026 Jun 17;14:1841816. doi: 10.3389/fped.2026.1841816 (PMC13318893; doi:10.3389/fped.2026.1841816)
Supplement: Supplementary file 10 [file Datasheet1.pdf]

Parameter optimization code for the total sample

```
#-*- coding: utf-8 -*-
"""
Multiple Machine Learning Algorithms Comparison (Optimized with GridSearchCV + Ensemble Learning) - ASD Severity Prediction (608 cases)
"""

import pandas as pd
import numpy as np
from sklearn.model_selection import train_test_split, cross_val_score, GridSearchCV, StratifiedKFold
from sklearn.preprocessing import StandardScaler
from sklearn.metrics import (accuracy_score, precision_score, recall_score,
                             f1_score, roc_auc_score, matthews_corrcoef,
                             balanced_accuracy_score)
import matplotlib.pyplot as plt
import seaborn as sns
from sklearn.ensemble import RandomForestClassifier, GradientBoostingClassifier, VotingClassifier
from sklearn.svm import SVC
from sklearn.linear_model import LogisticRegression
from sklearn.neighbors import KNeighborsClassifier
from sklearn.tree import DecisionTreeClassifier
import warnings
import os
import json
from datetime import datetime

warnings.filterwarnings('ignore')

# Create output directory if it doesn't exist
output_dir = "output"
if not os.path.exists(output_dir):
    os.makedirs(output_dir)

# Set Chinese font (optional, keep for compatibility)
plt.rcParams['font.sans-serif'] = ['SimHei']
plt.rcParams['axes.unicode_minus'] = False

# Read data
data = pd.read_excel("608.xlsx",
    usecols=['Age', 'HAZ', 'WAZ', 'BAZ', 'Adaptive', 'Gross_Motor', 'Fine_Motor', 'Language', 'Social_Skills', 'Is_Severe'])
```

```
print("Basic data information:")
print(f"Data shape: {data.shape}")
print("Class distribution:")
print(data['Is_Severe'].value_counts())

# Split data
x_train, x_test, y_train, y_test = train_test_split(
    data.iloc[:,0:9], data.iloc[:,9], test_size=0.2, random_state=42)

# Standardization
scaler = StandardScaler()
x_train_scaled = scaler.fit_transform(x_train)
x_test_scaled = scaler.transform(x_test)

# ==================== Data Augmentation Strategy ====================
print("\nApplying data augmentation strategy...")
try:
    from imblearn.over_sampling import SMOTE
    # Automatically adjust k_neighbors to prevent errors with small samples
    min_samples = y_train.value_counts().min()
    k_neighbors = min(5, min_samples - 1) if min_samples > 1 else 1

    smote = SMOTE(random_state=42, k_neighbors=k_neighbors)
    x_train_scaled, y_train = smote.fit_resample(x_train_scaled, y_train)
    print(f"✅ SMOTE data augmentation successful! Training samples increased from {len(x_train)} to {len(x_train_scaled)}")
except ImportError:
    print("    imblearn library not detected, switching to random oversampling...")
    # Manual simple oversampling implementation
    import numpy as np
    y_train_np = y_train.values if hasattr(y_train, 'values') else y_train
    classes, counts = np.unique(y_train_np, return_counts=True)
    max_count = counts.max()

    x_resampled = []
    y_resampled = []

    for cls in classes:
        cls_idx = np.where(y_train_np == cls)[0]
        x_cls = x_train_scaled[cls_idx]

        if len(x_cls) < max_count:
            indices = np.random.choice(len(x_cls), max_count, replace=True)
            x_cls = x_cls[indices]
```

```
            x_resampled.append(x_cls)
            y_resampled.append(np.full(max_count, cls))

        x_train_scaled = np.vstack(x_resampled)
        y_train = np.concatenate(y_resampled)
        print(f"✅ Random oversampling successful! Training samples increased to {len(y_train)}")
    except Exception as e:
        print(f"❌ Data augmentation failed: {e}, continuing with original data...")

    # Cross-validation strategy
    cv_strategy = StratifiedKFold(n_splits=5, shuffle=True, random_state=42)

    # Define models and their parameter grids
    model_params = {
        'Random Forest': {
            'model': RandomForestClassifier(random_state=42, n_jobs=-1),
            'params': {
                'n_estimators': [100, 200, 300],
                'max_depth': [4, 5, 10, 20, None], # Include original values
                'min_samples_split': [2, 5],
                'min_samples_leaf': [1, 2, 4],
                'max_features': ['sqrt'],
                'class_weight': [None, 'balanced'] # Optional class balancing
            }
        },
        'Gradient Boosting': {
            'model': GradientBoostingClassifier(random_state=42),
            'params': {
                'n_estimators': [50, 100, 200], # Include original values
                'learning_rate': [0.05, 0.1, 0.2],
                'max_depth': [3, 4, 5], # Include original values
                'subsample': [0.8, 1.0]
            }
        },
        'Support Vector Machine': {
            'model': SVC(probability=True, random_state=42),
            'params': {
                'C': [0.1, 1, 10, 100],
                'kernel': ['rbf', 'linear'],
                'gamma': ['scale', 'auto', 0.1, 0.01],
                'class_weight': [None, 'balanced']
            }
        },
```

```
    'Logistic Regression': {
        'model': LogisticRegression(random_state=42, max_iter=2000),
        'params': {
            'C': [0.1, 1, 10, 100],
            'solver': ['liblinear', 'lbfgs'],
            'penalty': ['l2'],
            'class_weight': [None, 'balanced']
        }
    },
    'K-Nearest Neighbors': {
        'model': KNeighborsClassifier(),
        'params': {
            'n_neighbors': [3, 5, 7, 9, 11],
            'weights': ['uniform', 'distance'],
            'p': [1, 2]
        }
    },

}

# Store model performance metrics
results = {
    'Model': [],
    'Accuracy': [], 'Precision': [], 'Recall': [], 'F1 Score': [],
    'AUC-ROC': [], 'Balanced Accuracy': [], 'Matthews Correlation Coefficient': [],
'Cross-validation Accuracy': []
}

# Store best parameters for each model
best_params_records = {
    'Model': [],
    'Best Parameters': [],
    'Best CV Score': [],
    'Parameter Grid': []
}

print("Starting training and evaluation of each model (GridSearchCV optimization + Ensemble
Learning)...")
print("=" * 80)

optimized_models = {}

# Train and evaluate each model
for model_name, mp in model_params.items():
```

```
    print(f"\nOptimizing and training {model_name}...")

    # Use GridSearchCV for hyperparameter optimization
    grid = GridSearchCV(mp['model'], mp['params'], cv=cv_strategy, scoring='accuracy',
n_jobs=-1)
    grid.fit(x_train_scaled, y_train)

    best_model = grid.best_estimator_
    optimized_models[model_name] = best_model

    print(f"    Best parameters: {grid.best_params_}")
    print(f"    Best CV score: {grid.best_score_:.4f}")

    # Record best parameters
    best_params_records['Model'].append(model_name)
    best_params_records['Best Parameters'].append(grid.best_params_)
    best_params_records['Best CV Score'].append(grid.best_score_)
    best_params_records['Parameter Grid'].append(mp['params'])

    # Predictions
    y_pred = best_model.predict(x_test_scaled)
    y_prob = best_model.predict_proba(x_test_scaled)

    # Calculate metrics
    accuracy = accuracy_score(y_test, y_pred)
    precision = precision_score(y_test, y_pred, average='binary', pos_label=2)
    recall = recall_score(y_test, y_pred, average='binary', pos_label=2)
    f1 = f1_score(y_test, y_pred, average='binary', pos_label=2)

    if hasattr(best_model, "predict_proba"):
        auc_roc = roc_auc_score(y_test, y_prob[:, 1])
    else:
        auc_roc = 0.0

    balanced_acc = balanced_accuracy_score(y_test, y_pred)
    mcc = matthews_corrcoef(y_test, y_pred)

    cv_scores = cross_val_score(best_model, x_train_scaled, y_train, cv=cv_strategy,
scoring='accuracy')
    cv_mean = cv_scores.mean()

    # Store results
    results['Model'].append(model_name)
    results['Accuracy'].append(accuracy)
```

```
        results['Precision'].append(precision)
        results['Recall'].append(recall)
        results['F1 Score'].append(f1)
        results['AUC-ROC'].append(auc_roc)
        results['Balanced Accuracy'].append(balanced_acc)
        results['Matthews Correlation Coefficient'].append(mcc)
        results['Cross-validation Accuracy'].append(cv_mean)

        print(f"{model_name} optimized results: Acc={accuracy:.3f} | F1={f1:.3f} |
AUC={auc_roc:.3f}")

# =================== Add Ensemble Model (Voting Classifier) ===================
print("\nTraining ensemble model (Soft Voting)...")

# 1. Sort models by CV scores
model_cv_scores = []
for name, model in optimized_models.items():
        idx = results['Model'].index(name)
        score = results['Cross-validation Accuracy'][idx]
        model_cv_scores.append((name, model, score))

# Sort in descending order
model_cv_scores.sort(key=lambda x: x[2], reverse=True)

# 2. Select Top 4 models (or all if less than 4)
top_n = 4
selected_models = model_cv_scores[:top_n]

print(f"\nTop {len(selected_models)} models for ensemble:")
estimators = []
weights = []
for name, model, score in selected_models:
        print(f"    {name}: CV Score = {score:.4f}")
        estimators.append((name, model))
        weights.append(score**2) # Use squared CV scores as weights to amplify differences

# 3. Build weighted VotingClassifier
print(f"Training weighted ensemble model (Weighted Soft Voting)...")
voting_clf = VotingClassifier(estimators=estimators, voting='soft', weights=weights)
voting_clf.fit(x_train_scaled, y_train)

y_pred_vote = voting_clf.predict(x_test_scaled)
y_prob_vote = voting_clf.predict_proba(x_test_scaled)
```

```
# Calculate ensemble model metrics
acc_vote = accuracy_score(y_test, y_pred_vote)
prec_vote = precision_score(y_test, y_pred_vote, average='binary', pos_label=2)
rec_vote = recall_score(y_test, y_pred_vote, average='binary', pos_label=2)
f1_vote = f1_score(y_test, y_pred_vote, average='binary', pos_label=2)
auc_vote = roc_auc_score(y_test, y_prob_vote[:, 1])
bal_acc_vote = balanced_accuracy_score(y_test, y_pred_vote)
mcc_vote = matthews_corrcoef(y_test, y_pred_vote)
cv_vote = cross_val_score(voting_clf, x_train_scaled, y_train, cv=cv_strategy,
scoring='accuracy').mean()

# Add ensemble model results
results['Model'].append('Ensemble Model(Voting)')
results['Accuracy'].append(acc_vote)
results['Precision'].append(prec_vote)
results['Recall'].append(rec_vote)
results['F1 Score'].append(f1_vote)
results['AUC-ROC'].append(auc_vote)
results['Balanced Accuracy'].append(bal_acc_vote)
results['Matthews Correlation Coefficient'].append(mcc_vote)
results['Cross-validation Accuracy'].append(cv_vote)

print(f"Ensemble Model results: Acc={acc_vote:.3f} | F1={f1_vote:.3f} | AUC={auc_vote:.3f}")

# =================== Save Parameters to Table ===================
print("\n" + "=" * 80)
print("Best Parameters Records")
print("=" * 80)

# Create DataFrame for best parameters
best_params_df = pd.DataFrame(best_params_records)

# Format parameters for better readability
def format_params(params_dict):
    if isinstance(params_dict, dict):
        formatted = []
        for key, value in params_dict.items():
            formatted.append(f"{key}: {value}")
        return "\n".join(formatted)
    return str(params_dict)

best_params_df['Best Parameters (Formatted)'] = best_params_df['Best
Parameters'].apply(format_params)
```

```
# Calculate total parameter combinations
def count_param_combinations(param_grid):
    total = 1
    for key, values in param_grid.items():
        total *= len(values) if isinstance(values, list) else 1
    return total

best_params_df['Parameter Combinations'] = best_params_df['Parameter Grid'].apply(count_param_combinations)
best_params_df['Best CV Score'] = best_params_df['Best CV Score'].round(4)

# Select columns for display
display_columns = ['Model', 'Best CV Score', 'Parameter Combinations', 'Best Parameters (Formatted)']
print(best_params_df[display_columns].to_string())

# Save to CSV
output_path = os.path.join(output_dir, '608_Best_Parameters_Table.csv')
best_params_df.to_csv(output_path, index=False)
print(f"\n✅ Best parameters table saved as: {output_path}")

# Save detailed parameters to JSON
output_json_path = os.path.join(output_dir, '608_Detailed_Parameters.json')
params_details = {
    'timestamp': datetime.now().strftime('%Y-%m-%d %H:%M:%S'),
    'data_info': {
        'dataset': '608.xlsx',
        'total_samples': data.shape[0],
        'training_samples': len(y_train),
        'test_samples': len(y_test),
        'features': list(data.columns[:-1])
    },
    'best_parameters': best_params_records,
    'ensemble_info': {
        'selected_models': [name for name, _, _ in selected_models],
        'weights': weights,
        'voting_method': 'soft'
    }
}

with open(output_json_path, 'w', encoding='utf-8') as f:
    json.dump(params_details, f, indent=2, ensure_ascii=False)
print(f"✅ Detailed parameters saved as: {output_json_path}")
```

```
# Create results DataFrame
results_df = pd.DataFrame(results)
results_df.set_index('Model', inplace=True)
print("\n" + "=" * 80)
print("Performance Comparison Summary (Optimized+Ensemble):")
print("=" * 80)
print(results_df.round(3))

# Find best model for each metric
print("\nBest models for each metric:")
for metric in ['Accuracy', 'Precision', 'Recall', 'F1 Score', 'AUC-ROC', 'Balanced Accuracy', 'Matthews Correlation Coefficient', 'Cross-validation Accuracy']:
    best_model = results_df[metric].idxmax()
    best_score = results_df[metric].max()
    print(f"{metric}: {best_model} ({best_score:.3f})")

# =================== Generate separate charts and save to output directory ===================

# 1. Bar Chart - Comprehensive Performance Comparison
print("\nGenerating comprehensive performance bar chart...")
try:
    plt.figure(figsize=(14, 8))
    metrics_for_bar = ['Accuracy', 'Precision', 'Recall', 'F1 Score', 'AUC-ROC', 'Balanced Accuracy']

    x = np.arange(len(results_df.index))
    width = 0.14
    colors = plt.cm.Set3(np.linspace(0, 1, len(metrics_for_bar)))

    ax = plt.subplot(111)

    for i, metric in enumerate(metrics_for_bar):
        offset = width * i - (width * (len(metrics_for_bar) - 1) / 2)
        values = results_df[metric].values
        bars = ax.bar(x + offset, values, width, label=metric, color=colors[i], alpha=0.8)

        for bar, value in zip(bars, values):
            height = bar.get_height()
            ax.text(bar.get_x() + bar.get_width()/2., height + 0.01,
                    f'{value:.3f}', ha='center', va='bottom', fontsize=4)

    ax.set_xlabel('Model')
    ax.set_ylabel('Score')
```

```
    ax.set_title('Comprehensive Performance Comparison of Models for 24-71 Months
(Optimized+Ensemble)', size=14, fontweight='bold')
    ax.set_xticks(x)
    ax.set_xticklabels(results_df.index)
    ax.set_ylim(0, 1.05)
    ax.legend(title='Evaluation Metrics', bbox_to_anchor=(1.05, 1), loc='upper left')
    ax.grid(True, alpha=0.3, axis='y')

    plt.tight_layout()
    # Save as PDF in output directory
    output_path = os.path.join(output_dir, '608_Comprehensive_Performance_Bar_Chart.pdf')
    plt.savefig(output_path, dpi=300, bbox_inches='tight')
    print(f"✅ Saved as: {output_path}")
    plt.show()

    # 2. Bar Chart - AUC-ROC Comparison
    print("\nGenerating AUC-ROC comparison chart...")
    plt.figure(figsize=(10, 6))
    sorted_auc = results_df['AUC-ROC'].sort_values(ascending=True)
    colors = plt.cm.Set3(np.linspace(0, 1, len(sorted_auc)))
    bars = plt.barh(range(len(sorted_auc)), sorted_auc.values, color=colors)
    plt.yticks(range(len(sorted_auc)), sorted_auc.index)
    plt.xlabel('AUC-ROC')
    plt.title('AUC-ROC Comparison of Models (Optimized+Ensemble)')
    for i, bar in enumerate(bars):
        width = bar.get_width()
        plt.text(width + 0.01, bar.get_y() + bar.get_height()/2,
                 f'{width:.3f}', ha='left', va='center')
    plt.tight_layout()
    # Save as PDF in output directory
    output_path = os.path.join(output_dir, '608_AUC_ROC_Comparison_Chart.pdf')
    plt.savefig(output_path, dpi=300, bbox_inches='tight')
    print(f"✅ Saved as: {output_path}")
    plt.show()

    # ==================== Combined Chart: Subplot Format ====================

    print("\nGenerating combined comparison chart...")
    fig, (ax1, ax2) = plt.subplots(1, 2, figsize=(20, 9))

    # 1. Left subplot
    metrics_for_bar = ['Accuracy', 'Precision', 'Recall', 'F1 Score', 'AUC-ROC', 'Balanced
Accuracy']
    x = np.arange(len(results_df.index))
```

```
    width = 0.13
    colors = plt.cm.Set3(np.linspace(0, 1, len(metrics_for_bar)))

    for i, metric in enumerate(metrics_for_bar):
        offset = width * i - (width * (len(metrics_for_bar) - 1) / 2)
        values = results_df[metric].values
        bars = ax1.bar(x + offset, values, width, label=metric, color=colors[i], alpha=0.8)

        for bar, value in zip(bars, values):
            height = bar.get_height()
            ax1.text(bar.get_x() + bar.get_width()/2., height + 0.01,
                    f'{value:.3f}', ha='center', va='bottom', fontsize=5)

    ax1.set_xlabel('Model', fontsize=11)
    ax1.set_ylabel('Score', fontsize=11)
    ax1.set_title('(C) Comprehensive Performance Comparison (Optimized+Ensemble)', size=14,
fontweight='bold', pad=15)
    ax1.set_xticks(x)
    ax1.set_xticklabels(results_df.index, rotation=15)
    ax1.set_ylim(0, 1.05)
    ax1.legend(title='Evaluation Metrics', fontsize=8, title_fontsize=9)
    ax1.grid(True, alpha=0.3, axis='y')

    # 2. Right subplot
    sorted_auc = results_df['AUC-ROC'].sort_values(ascending=True)
    normalized_auc = (sorted_auc.values - sorted_auc.min()) / (sorted_auc.max() -
sorted_auc.min() + 1e-8)
    colors_auc = plt.cm.Blues(normalized_auc * 0.7 + 0.3)

    bars = ax2.barh(range(len(sorted_auc)), sorted_auc.values, color=colors_auc, height=0.6)
    ax2.set_yticks(range(len(sorted_auc)))
    ax2.set_yticklabels(sorted_auc.index)
    ax2.set_xlabel('AUC-ROC Value', fontsize=11)
    ax2.set_title('(D) AUC-ROC Comparison of Models (Optimized+Ensemble)', size=14,
fontweight='bold', pad=15)
    ax2.set_xlim(0, 1.05)

    for i, bar in enumerate(bars):
        width = bar.get_width()
        label_x = width + 0.01
        if label_x > 1.0:
            label_x = width - 0.02
            text_color = 'white'
        else:
```

```
                text_color = 'black'

            ax2.text(label_x, bar.get_y() + bar.get_height()/2,
                    f'{width:.3f}', ha='left', va='center', fontsize=10,
                    fontweight='bold', color=text_color)

    plt.suptitle('ASD Severity Prediction Model Performance for 24-71 Months
(Optimized+Ensemble)', fontsize=18, fontweight='bold', y=0.98)
    plt.tight_layout(rect=[0, 0, 1, 0.95])
    # Save as PDF in output directory
    output_path = os.path.join(output_dir, '608_Combined_Comparison_Chart.pdf')
    plt.savefig(output_path, dpi=300, bbox_inches='tight')
    print(f"✅ Saved as: {output_path}")
    plt.show()

except Exception as e:
    print(f"Error during plotting: {e}")

# =================== Detailed Performance Comparison Table ===================
print("\n" + "=" * 100)
print("Detailed Performance Comparison Table (Suitable for Research Papers)")
print("=" * 100)

detailed_comparison = results_df.round(4)
detailed_comparison['Rank'] =
detailed_comparison.mean(axis=1).rank(ascending=False).astype(int)
detailed_comparison = detailed_comparison.sort_values('Rank')

print("\nDetailed Model Performance Comparison (Sorted by Overall Rank):")
print(detailed_comparison)

# Save detailed comparison table as CSV
output_path = os.path.join(output_dir, '608_Detailed_Performance_Comparison.csv')
detailed_comparison.to_csv(output_path)
print(f"\n✅ Detailed performance comparison table saved as: {output_path}")

best_overall = detailed_comparison.index[0]
print(f"\n    Overall Recommended Best Model: {best_overall}")
print("Reason: Performs well and balanced across multiple evaluation metrics")

print("\n" + "=" * 80)
print("Optimized analysis completed!")
print(f"All results have been saved in the '{output_dir}' directory:")
print(f"1. 608_Best_Parameters_Table.csv - Best hyperparameters for each model")
```

```
print(f"2. 608_Detailed_Parameters.json - Detailed parameter information")
print(f"3. 608_Detailed_Performance_Comparison.csv - Performance metrics")
print(f"4. Various performance charts in PDF format")
print("=" * 80)
```

Parameter optimization code for the low-age group

```
# -*- coding: utf-8 -*-
"""
Multiple Machine Learning Algorithms Comparison (with GridSearchCV Optimization + Ensemble Learning) - ASD Severity Prediction (461 Cases)
"""

import pandas as pd
import numpy as np
from sklearn.model_selection import train_test_split, cross_val_score, GridSearchCV, StratifiedKFold
from sklearn.preprocessing import StandardScaler
from sklearn.metrics import (accuracy_score, precision_score, recall_score,
                             f1_score, roc_auc_score, matthews_corrcoef,
                             balanced_accuracy_score)
import matplotlib.pyplot as plt
import seaborn as sns
from sklearn.ensemble import RandomForestClassifier, GradientBoostingClassifier, VotingClassifier
from sklearn.svm import SVC
from sklearn.linear_model import LogisticRegression
from sklearn.neighbors import KNeighborsClassifier
from sklearn.tree import DecisionTreeClassifier
import warnings
import os
import json
from datetime import datetime

warnings.filterwarnings('ignore')

# Create output directory if it doesn't exist
output_dir = 'output'
if not os.path.exists(output_dir):
    os.makedirs(output_dir)

# Set plotting parameters for PDF output
plt.rcParams['font.sans-serif'] = ['SimHei', 'DejaVu Sans']
plt.rcParams['axes.unicode_minus'] = False
plt.rcParams['pdf.fonttype'] = 42
plt.rcParams['ps.fonttype'] = 42

# Read data
```

```
data = pd.read_excel('461.xlsx',
        usecols=['HAZ', 'WAZ','BAZ','Adaptive', 'Gross_Motor', 'Fine_Motor', 'Language',
'Social_Skills', 'Is_Severe'])

print("Data Information:")
print(f"Data Shape: {data.shape}")
print("Class Distribution:")
print(data['Is_Severe'].value_counts())

# Split data (maintaining random_state=10 from original code)
x_train, x_test, y_train, y_test = train_test_split(
        data.iloc[:,0:8], data.iloc[:,8], test_size=0.2, random_state=30)

# Standardization
scaler = StandardScaler()
x_train_scaled = scaler.fit_transform(x_train)
x_test_scaled = scaler.transform(x_test)

# =================== Data Augmentation Strategy ===================
print("\nApplying data augmentation strategy...")
try:
        from imblearn.over_sampling import SMOTE
        # Automatically adjust k_neighbors to avoid errors with small samples
        min_samples = y_train.value_counts().min()
        k_neighbors = min(5, min_samples - 1) if min_samples > 1 else 1

        smote = SMOTE(random_state=42, k_neighbors=k_neighbors)
        x_train_scaled, y_train = smote.fit_resample(x_train_scaled, y_train)
        print(f" ✅ SMOTE data augmentation successful! Training samples increased from
{len(x_train)} to {len(x_train_scaled)}")
except ImportError:
        print("    imblearn library not detected, switching to random oversampling...")
        # Manual simple oversampling implementation
        import numpy as np
        y_train_np = y_train.values if hasattr(y_train, 'values') else y_train
        classes, counts = np.unique(y_train_np, return_counts=True)
        max_count = counts.max()

        x_resampled = []
        y_resampled = []

        for cls in classes:
                cls_idx = np.where(y_train_np == cls)[0]
                x_cls = x_train_scaled[cls_idx]
```

```
            if len(x_cls) < max_count:
                indices = np.random.choice(len(x_cls), max_count, replace=True)
                x_cls = x_cls[indices]

            x_resampled.append(x_cls)
            y_resampled.append(np.full(max_count, cls))

        x_train_scaled = np.vstack(x_resampled)
        y_train = np.concatenate(y_resampled)
        print(f"✅ Random oversampling successful! Training samples increased to {len(y_train)}")
except Exception as e:
        print(f"❌ Data augmentation failed: {e}, continuing with original data...")

# Cross-validation strategy
cv_strategy = StratifiedKFold(n_splits=5, shuffle=True, random_state=42)

# Define models and parameter grids (optimized for medium-sized dataset)
model_params = {
        'Random Forest': {
            'model': RandomForestClassifier(random_state=20, n_jobs=-1),
            'params': {
                'n_estimators': [80, 100, 200, 300],
                'max_depth': [4, 5, 10, 15, None],
                'min_samples_split': [2, 3, 5],
                'min_samples_leaf': [1, 2, 4],
                'max_features': ['sqrt'],
                'class_weight': [None, 'balanced']
            }
        },
        'Gradient Boosting': {
            'model': GradientBoostingClassifier(random_state=42),
            'params': {
                'n_estimators': [50, 100, 200],
                'learning_rate': [0.05, 0.1, 0.2],
                'max_depth': [3, 4, 5],
                'subsample': [0.8, 1.0]
            }
        },
        'SVM': {
            'model': SVC(probability=True, random_state=42),
            'params': {
                'C': [0.1, 1, 10, 50],
                'kernel': ['rbf', 'linear'],
```

```
            'gamma': ['scale', 'auto', 0.1, 0.01],
            'class_weight': [None, 'balanced']
        }
    },
    'Logistic Regression': {
        'model': LogisticRegression(random_state=42, max_iter=2000),
        'params': {
            'C': [0.1, 1, 10, 100],
            'solver': ['liblinear', 'lbfgs'],
            'penalty': ['l2'],
            'class_weight': [None, 'balanced']
        }
    },
    'KNN': {
        'model': KNeighborsClassifier(),
        'params': {
            'n_neighbors': [3, 5, 7, 9, 11],
            'weights': ['uniform', 'distance'],
            'p': [1, 2]
        }
    },

}

# Store performance metrics for each model
results = {
    'Model': [],
    'Accuracy': [], 'Precision': [], 'Recall': [], 'F1-Score': [],
    'AUC-ROC': [], 'Balanced Accuracy': [], 'MCC': [], 'CV Accuracy': []
}

# Store best parameters for each model
best_params_records = {
    'Model': [],
    'Best Parameters': [],
    'Best CV Score': [],
    'Parameter Grid': [],
    'Search Space Size': []
}

print("Starting model training and evaluation (GridSearchCV Optimization + Ensemble
Learning)...")
print("=" * 80)
```

```
optimized_models = {}

# Train and evaluate each model
for model_name, mp in model_params.items():
    print(f"\nOptimizing and training {model_name}...")

    # Calculate search space size
    search_space_size = 1
    for key, values in mp['params'].items():
        search_space_size *= len(values)
    print(f"    Search space: {search_space_size} parameter combinations")

    # Hyperparameter optimization using GridSearchCV
    grid = GridSearchCV(mp['model'], mp['params'], cv=cv_strategy, scoring='accuracy',
n_jobs=-1, verbose=0)
    grid.fit(x_train_scaled, y_train)

    best_model = grid.best_estimator_
    optimized_models[model_name] = best_model

    print(f"    Best CV score: {grid.best_score_:.4f}")
    print(f"    Best parameters: {grid.best_params_}")

    # Record best parameters
    best_params_records['Model'].append(model_name)
    best_params_records['Best Parameters'].append(grid.best_params_)
    best_params_records['Best CV Score'].append(grid.best_score_)
    best_params_records['Parameter Grid'].append(mp['params'])
    best_params_records['Search Space Size'].append(search_space_size)

    # Predictions
    y_pred = best_model.predict(x_test_scaled)
    y_prob = best_model.predict_proba(x_test_scaled)

    # Calculate metrics
    accuracy = accuracy_score(y_test, y_pred)
    precision = precision_score(y_test, y_pred, average='binary', pos_label=2)
    recall = recall_score(y_test, y_pred, average='binary', pos_label=2)
    f1 = f1_score(y_test, y_pred, average='binary', pos_label=2)

    if hasattr(best_model, "predict_proba"):
        auc_roc = roc_auc_score(y_test, y_prob[:, 1])
    else:
        auc_roc = 0.0
```

```
        balanced_acc = balanced_accuracy_score(y_test, y_pred)
        mcc = matthews_corrcoef(y_test, y_pred)

        cv_scores = cross_val_score(best_model, x_train_scaled, y_train, cv=cv_strategy, scoring='accuracy')
        cv_mean = cv_scores.mean()

        # Store results
        results['Model'].append(model_name)
        results['Accuracy'].append(accuracy)
        results['Precision'].append(precision)
        results['Recall'].append(recall)
        results['F1-Score'].append(f1)
        results['AUC-ROC'].append(auc_roc)
        results['Balanced Accuracy'].append(balanced_acc)
        results['MCC'].append(mcc)
        results['CV Accuracy'].append(cv_mean)

        print(f"{model_name} optimized results: Acc={accuracy:.3f} | F1={f1:.3f} | AUC={auc_roc:.3f}")

# =================== Add Ensemble Model (Voting Classifier) ===================
print("\nTraining ensemble model (Soft Voting)...")

# 1. Sort models by CV score
model_cv_scores = []
for name, model in optimized_models.items():
        idx = results['Model'].index(name)
        score = results['CV Accuracy'][idx]
        model_cv_scores.append((name, model, score))

# Sort in descending order
model_cv_scores.sort(key=lambda x: x[2], reverse=True)

# 2. Select Top 4 models (use all if less than 4)
top_n = 4
selected_models = model_cv_scores[:top_n]

print(f"\nTop {len(selected_models)} models for ensemble:")
estimators = []
weights = []
for name, model, score in selected_models:
        print(f"    {name}: CV Score = {score:.4f}")
```

```
        estimators.append((name, model))
        weights.append(score**2) # Use square of CV score as weight to increase differentiation

# 3. Build weighted VotingClassifier
print(f"Training weighted ensemble model (Weighted Soft Voting)...")
voting_clf = VotingClassifier(estimators=estimators, voting='soft', weights=weights)
voting_clf.fit(x_train_scaled, y_train)

y_pred_vote = voting_clf.predict(x_test_scaled)
y_prob_vote = voting_clf.predict_proba(x_test_scaled)

# Calculate ensemble model metrics
acc_vote = accuracy_score(y_test, y_pred_vote)
prec_vote = precision_score(y_test, y_pred_vote, average='binary', pos_label=2)
rec_vote = recall_score(y_test, y_pred_vote, average='binary', pos_label=2)
f1_vote = f1_score(y_test, y_pred_vote, average='binary', pos_label=2)
auc_vote = roc_auc_score(y_test, y_prob_vote[:, 1])
bal_acc_vote = balanced_accuracy_score(y_test, y_pred_vote)
mcc_vote = matthews_corrcoef(y_test, y_pred_vote)
cv_vote = cross_val_score(voting_clf, x_train_scaled, y_train, cv=cv_strategy,
scoring='accuracy').mean()

# Add ensemble model results
results['Model'].append('Ensemble(Voting)')
results['Accuracy'].append(acc_vote)
results['Precision'].append(prec_vote)
results['Recall'].append(rec_vote)
results['F1-Score'].append(f1_vote)
results['AUC-ROC'].append(auc_vote)
results['Balanced Accuracy'].append(bal_acc_vote)
results['MCC'].append(mcc_vote)
results['CV Accuracy'].append(cv_vote)

print(f"Ensemble model results: Acc={acc_vote:.3f} | F1={f1_vote:.3f} | AUC={auc_vote:.3f}")

# ==================== Save Parameters to Table ====================
print("\n" + "=" * 80)
print("Best Parameters Records")
print("=" * 80)

# Create DataFrame for best parameters
best_params_df = pd.DataFrame(best_params_records)

# Format parameters for better readability
```

```
def format_params(params_dict):
    if isinstance(params_dict, dict):
        formatted = []
        for key, value in params_dict.items():
            if isinstance(value, (list, tuple, np.ndarray)):
                value = str(value)
            formatted.append(f"{key}: {value}")
        return "\n".join(formatted)
    return str(params_dict)

# Create formatted columns
best_params_df['Best Parameters (Formatted)'] = best_params_df['Best Parameters'].apply(format_params)
best_params_df['Parameter Grid (Formatted)'] = best_params_df['Parameter Grid'].apply(format_params)

# Format the Best CV Score
best_params_df['Best CV Score'] = best_params_df['Best CV Score'].round(4)

# Display the parameter table
print("\nModel Parameter Optimization Results:")
print("=" * 60)
for idx, row in best_params_df.iterrows():
    print(f"\n{row['Model']}:")
    print(f"  Search Space Size: {row['Search Space Size']}")
    print(f"  Best CV Score: {row['Best CV Score']:.4f}")
    print(f"  Best Parameters:")
    for key, value in row['Best Parameters'].items():
        print(f"    {key}: {value}")

# Save to CSV with detailed formatting
params_csv_path = os.path.join(output_dir, '461_best_parameters_table.csv')

# Create a more readable version for CSV
readable_params_df = pd.DataFrame({
    'Model': best_params_df['Model'],
    'Search_Space_Size': best_params_df['Search Space Size'],
    'Best_CV_Score': best_params_df['Best CV Score'],
    'Best_Parameters': best_params_df['Best Parameters (Formatted)'],
    'Parameter_Grid': best_params_df['Parameter Grid (Formatted)']
})

readable_params_df.to_csv(params_csv_path, index=False, encoding='utf-8-sig')
print(f"\n✅ Best parameters table saved as: {params_csv_path}")
```

```
# Save to Excel with better formatting
params_excel_path = os.path.join(output_dir, '461_best_parameters_table.xlsx')
with pd.ExcelWriter(params_excel_path, engine='xlsxwriter') as writer:
    # Create detailed sheet
    detailed_data = []
    for idx, row in best_params_df.iterrows():
        for key, value in row['Best Parameters'].items():
            detailed_data.append({
                'Model': row['Model'],
                'Parameter': key,
                'Best Value': str(value),
                'Search Space': str(row['Parameter Grid'].get(key, 'N/A')),
                'Best CV Score': row['Best CV Score']
            })

    detailed_df = pd.DataFrame(detailed_data)
    detailed_df.to_excel(writer, sheet_name='Detailed Parameters', index=False)

    # Create summary sheet
    summary_df = pd.DataFrame({
        'Model': best_params_df['Model'],
        'Search Space Size': best_params_df['Search Space Size'],
        'Best CV Score': best_params_df['Best CV Score'],
        'Best Parameters': best_params_df['Best Parameters (Formatted)']
    })
    summary_df.to_excel(writer, sheet_name='Summary', index=False)

    # Create workbook and format
    workbook = writer.book

    # Format for summary sheet
    summary_format = workbook.add_format({'align': 'left', 'valign': 'top'})
    summary_sheet = writer.sheets['Summary']
    summary_sheet.set_column('A:A', 20)
    summary_sheet.set_column('B:B', 15)
    summary_sheet.set_column('C:C', 12)
    summary_sheet.set_column('D:D', 50, summary_format)

    # Format for detailed sheet
    detailed_sheet = writer.sheets['Detailed Parameters']
    detailed_sheet.set_column('A:A', 20)
    detailed_sheet.set_column('B:B', 25)
    detailed_sheet.set_column('C:C', 25)
```

```
        detailed_sheet.set_column('D:D', 30)
        detailed_sheet.set_column('E:E', 12)

print(f"✅ Best parameters table (Excel) saved as: {params_excel_path}")

# Save detailed parameters to JSON
params_json_path = os.path.join(output_dir, '461_detailed_parameters.json')
params_data = {
        'timestamp': datetime.now().strftime('%Y-%m-%d %H:%M:%S'),
        'data_info': {
                'dataset': '461.xlsx',
                'total_samples': data.shape[0],
                'training_samples': len(y_train),
                'test_samples': len(y_test),
                'features': list(data.columns[:-1]),
                'target_variable': 'Is_Severe'
        },
        'best_parameters': best_params_records,
        'ensemble_info': {
                'selected_models': [name for name, _, _ in selected_models],
                'weights': weights,
                'voting_method': 'soft'
        }
}

with open(params_json_path, 'w', encoding='utf-8') as f:
        json.dump(params_data, f, indent=2, ensure_ascii=False)
print(f"✅ Detailed parameters (JSON) saved as: {params_json_path}")

# Create results DataFrame
results_df = pd.DataFrame(results)
results_df.set_index('Model', inplace=True)
print("\n" + "=" * 80)
print("Model Performance Comparison Summary (Optimized + Ensemble):")
print("=" * 80)
print(results_df.round(3))

# Find best model for each metric
print("\nBest models for each metric:")
for metric in ['Accuracy', 'Precision', 'Recall', 'F1-Score', 'AUC-ROC', 'Balanced Accuracy', 'MCC',
'CV Accuracy']:
        best_model = results_df[metric].idxmax()
        best_score = results_df[metric].max()
        print(f"{metric}: {best_model} ({best_score:.3f})")
```

```
# =================== Generate separate charts and save as PDF ===================

# 1. Bar chart - Comprehensive performance comparison
print("\nGenerating comprehensive performance bar chart and saving as PDF...")
try:
    plt.figure(figsize=(14, 8))
    metrics_for_bar = ['Accuracy', 'Precision', 'Recall', 'F1-Score', 'AUC-ROC', 'Balanced Accuracy']

    x = np.arange(len(results_df.index))
    width = 0.14
    colors = plt.cm.Set3(np.linspace(0, 1, len(metrics_for_bar)))

    ax = plt.subplot(111)

    for i, metric in enumerate(metrics_for_bar):
        offset = width * i - (width * (len(metrics_for_bar) - 1) / 2)
        values = results_df[metric].values
        bars = ax.bar(x + offset, values, width, label=metric, color=colors[i], alpha=0.8)

        for bar, value in zip(bars, values):
            height = bar.get_height()
            ax.text(bar.get_x() + bar.get_width()/2., height + 0.01,
                    f'{value:.3f}', ha='center', va='bottom', fontsize=4)

    ax.set_xlabel('Model')
    ax.set_ylabel('Score')
    ax.set_title('Comprehensive Performance Comparison of Models for 24-47 Months (Optimized+Ensemble)', size=14, fontweight='bold')
    ax.set_xticks(x)
    ax.set_xticklabels(results_df.index)
    ax.set_ylim(0, 1.05)
    ax.legend(title='Evaluation Metrics', bbox_to_anchor=(1.05, 1), loc='upper left')
    ax.grid(True, alpha=0.3, axis='y')

    plt.tight_layout()
    # Save as PDF
    output_path = os.path.join(output_dir, '461_comprehensive_performance_bar.pdf')
    plt.savefig(output_path, dpi=300, bbox_inches='tight')
    print(f"✅ Comprehensive performance bar chart saved as: {output_path}")
    plt.show()

    # 2. Bar chart - AUC-ROC comparison
```

```
print("Generating AUC-ROC comparison chart and saving as PDF...")
plt.figure(figsize=(10, 6))
sorted_auc = results_df['AUC-ROC'].sort_values(ascending=True)
colors = plt.cm.Set3(np.linspace(0, 1, len(sorted_auc)))
bars = plt.barh(range(len(sorted_auc)), sorted_auc.values, color=colors)
plt.yticks(range(len(sorted_auc)), sorted_auc.index)
plt.xlabel('AUC-ROC')
plt.title('AUC-ROC Comparison of Models (Optimized + Ensemble)')
for i, bar in enumerate(bars):
    width = bar.get_width()
    plt.text(width + 0.01, bar.get_y() + bar.get_height()/2,
             f'{width:.3f}', ha='left', va='center')
plt.tight_layout()
# Save as PDF
output_path = os.path.join(output_dir, '461_AUC_ROC_comparison.pdf')
plt.savefig(output_path, dpi=300, bbox_inches='tight')
print(f"✅ AUC-ROC comparison chart saved as: {output_path}")
plt.show()

# =================== Combined chart: subplot format ===================

print("\nGenerating combined comparison chart and saving as PDF...")
fig, (ax1, ax2) = plt.subplots(1, 2, figsize=(20, 9))

# 1. Left subplot
metrics_for_bar = ['Accuracy', 'Precision', 'Recall', 'F1-Score', 'AUC-ROC', 'Balanced
Accuracy']
x = np.arange(len(results_df.index))
width = 0.13
colors = plt.cm.Set3(np.linspace(0, 1, len(metrics_for_bar)))

for i, metric in enumerate(metrics_for_bar):
    offset = width * i - (width * (len(metrics_for_bar) - 1) / 2)
    values = results_df[metric].values
    bars = ax1.bar(x + offset, values, width, label=metric, color=colors[i], alpha=0.8)

    for bar, value in zip(bars, values):
        height = bar.get_height()
        ax1.text(bar.get_x() + bar.get_width()/2., height + 0.01,
                 f'{value:.3f}', ha='center', va='bottom', fontsize=5)

ax1.set_xlabel('Model', fontsize=11)
ax1.set_ylabel('Score', fontsize=11)
ax1.set_title('(C) Comprehensive Performance Comparison (Optimized + Ensemble)', size=14,
```

```
fontweight='bold', pad=15)
    ax1.set_xticks(x)
    ax1.set_xticklabels(results_df.index, rotation=15)
    ax1.set_ylim(0, 1.05)
    ax1.legend(title='Evaluation Metrics', fontsize=8, title_fontsize=9)
    ax1.grid(True, alpha=0.3, axis='y')

    # 2. Right subplot
    sorted_auc = results_df['AUC-ROC'].sort_values(ascending=True)
    normalized_auc = (sorted_auc.values - sorted_auc.min()) / (sorted_auc.max() - 
sorted_auc.min() + 1e-8)
    colors_auc = plt.cm.Blues(normalized_auc * 0.7 + 0.3)

    bars = ax2.barh(range(len(sorted_auc)), sorted_auc.values, color=colors_auc, height=0.6)
    ax2.set_yticks(range(len(sorted_auc)))
    ax2.set_yticklabels(sorted_auc.index)
    ax2.set_xlabel('AUC-ROC Value', fontsize=11)
    ax2.set_title('(D) AUC-ROC Comparison (Optimized + Ensemble)', size=14, fontweight='bold', 
pad=15)
    ax2.set_xlim(0, 1.05)

    for i, bar in enumerate(bars):
        width = bar.get_width()
        label_x = width + 0.01
        if label_x > 1.0:
            label_x = width - 0.02
            text_color = 'white'
        else:
            text_color = 'black'

        ax2.text(label_x, bar.get_y() + bar.get_height()/2,
                 f'{width:.3f}', ha='left', va='center', fontsize=10,
                 fontweight='bold', color=text_color)

    plt.suptitle('ASD Severity Prediction Model Performance Comparison for 24-47 Months 
(Optimized + Ensemble)', fontsize=18, fontweight='bold', y=0.98)
    plt.tight_layout(rect=[0, 0, 1, 0.95])

    # Save as PDF
    output_path = os.path.join(output_dir, '461_combined_performance_comparison.pdf')
    plt.savefig(output_path, dpi=300, bbox_inches='tight')
    print(f"✅ Combined performance comparison chart saved as: {output_path}")
    plt.show()
```

```
except Exception as e:
        print(f"Error during plotting: {e}")

# =================== Detailed performance comparison table ====================
print("\n" + "=" * 100)
print("Detailed Performance Comparison Table (Suitable for Research Papers)")
print("=" * 100)

detailed_comparison = results_df.round(4)
detailed_comparison['Rank'] =
detailed_comparison.mean(axis=1).rank(ascending=False).astype(int)
detailed_comparison = detailed_comparison.sort_values('Rank')

print("\nDetailed performance comparison of models (sorted by overall rank):")
print(detailed_comparison)

# Save detailed results to CSV file
output_csv_path = os.path.join(output_dir, '461_model_performance_detailed.csv')
detailed_comparison.to_csv(output_csv_path, encoding='utf-8-sig')
print(f"✅ Detailed performance comparison table saved as: {output_csv_path}")

# Save detailed results to Excel file
output_excel_path = os.path.join(output_dir, '461_model_performance_detailed.xlsx')
detailed_comparison.to_excel(output_excel_path)
print(f"✅ Detailed performance comparison table saved as: {output_excel_path}")

best_overall = detailed_comparison.index[0]
print(f"\n    Recommended best overall model: {best_overall}")
print("Reason: Balanced and excellent performance across multiple evaluation metrics")

print("\n" + "=" * 80)
print("Optimized analysis complete!")
print("\nGenerated the following PDF files in 'output' directory:")
print(f"1. {os.path.join(output_dir, '461_comprehensive_performance_bar.pdf')}")
print(f"2. {os.path.join(output_dir, '461_AUC_ROC_comparison.pdf')}")
print(f"3. {os.path.join(output_dir, '461_combined_performance_comparison.pdf')}")
print("\nGenerated the following data files in 'output' directory:")
print(f"1. {output_csv_path}")
print(f"2. {output_excel_path}")
print(f"3. {params_csv_path} (Parameter records)")
print(f"4. {params_excel_path} (Parameter records - Excel)")
print(f"5. {params_json_path} (Detailed parameters - JSON)")
print("=" * 80)
```

Parameter optimization code for the high-age group

```
#-*- coding: utf-8 -*-
"""
Multiple Machine Learning Algorithms Comparison (Optimized with GridSearchCV + Ensemble Learning) - ASD Severity Prediction (608 cases)
"""

import pandas as pd
import numpy as np
from sklearn.model_selection import train_test_split, cross_val_score, GridSearchCV, StratifiedKFold
from sklearn.preprocessing import StandardScaler
from sklearn.metrics import (accuracy_score, precision_score, recall_score,
                             f1_score, roc_auc_score, matthews_corrcoef,
                             balanced_accuracy_score)
import matplotlib.pyplot as plt
import seaborn as sns
from sklearn.ensemble import RandomForestClassifier, GradientBoostingClassifier, VotingClassifier
from sklearn.svm import SVC
from sklearn.linear_model import LogisticRegression
from sklearn.neighbors import KNeighborsClassifier
from sklearn.tree import DecisionTreeClassifier
import warnings
import os
import json
from datetime import datetime

warnings.filterwarnings('ignore')

# Create output directory if it doesn't exist
output_dir = "output"
if not os.path.exists(output_dir):
    os.makedirs(output_dir)

# Set Chinese font (optional, keep for compatibility)
plt.rcParams['font.sans-serif'] = ['SimHei']
plt.rcParams['axes.unicode_minus'] = False

# Read data
data = pd.read_excel("147.xlsx",
    usecols=[ 'HAZ', 'WAZ', 'BAZ', 'Adaptive', 'Gross_Motor', 'Fine_Motor', 'Language', 'Social_Skills', 'Is_Severe'])
```

```
print("Basic data information:")
print(f"Data shape: {data.shape}")
print("Class distribution:")
print(data['Is_Severe'].value_counts())

# Split data
x_train, x_test, y_train, y_test = train_test_split(
    data.iloc[:,0:8], data.iloc[:,8], test_size=0.3, random_state=42)

# Standardization
scaler = StandardScaler()
x_train_scaled = scaler.fit_transform(x_train)
x_test_scaled = scaler.transform(x_test)

# =================== Data Augmentation Strategy ===================
print("\nApplying data augmentation strategy...")
try:
    from imblearn.over_sampling import SMOTE
    # Automatically adjust k_neighbors to prevent errors with small samples
    min_samples = y_train.value_counts().min()
    k_neighbors = min(5, min_samples - 1) if min_samples > 1 else 1

    smote = SMOTE(random_state=42, k_neighbors=k_neighbors)
    x_train_scaled, y_train = smote.fit_resample(x_train_scaled, y_train)
    print(f"✅ SMOTE data augmentation successful! Training samples increased from {len(x_train)} to {len(x_train_scaled)}")
except ImportError:
    print("    imblearn library not detected, switching to random oversampling...")
    # Manual simple oversampling implementation
    import numpy as np
    y_train_np = y_train.values if hasattr(y_train, 'values') else y_train
    classes, counts = np.unique(y_train_np, return_counts=True)
    max_count = counts.max()

    x_resampled = []
    y_resampled = []

    for cls in classes:
        cls_idx = np.where(y_train_np == cls)[0]
        x_cls = x_train_scaled[cls_idx]

        if len(x_cls) < max_count:
            indices = np.random.choice(len(x_cls), max_count, replace=True)
```

```
                x_cls = x_cls[indices]

            x_resampled.append(x_cls)
            y_resampled.append(np.full(max_count, cls))

        x_train_scaled = np.vstack(x_resampled)
        y_train = np.concatenate(y_resampled)
        print(f"✅ Random oversampling successful! Training samples increased to {len(y_train)}")
except Exception as e:
        print(f"❌ Data augmentation failed: {e}, continuing with original data...")

# Cross-validation strategy
cv_strategy = StratifiedKFold(n_splits=5, shuffle=True, random_state=42)

# Define models and their parameter grids
model_params = {
        'Random Forest': {
                'model': RandomForestClassifier(random_state=42, n_jobs=-1),
                'params': {
                        'n_estimators': [100, 200, 300],
                        'max_depth': [4, 5, 10, 20, None], # Include original values
                        'min_samples_split': [2, 5],
                        'min_samples_leaf': [1, 2, 4],
                        'max_features': ['sqrt'],
                        'class_weight': [None, 'balanced'] # Optional class balancing
                }
        },
        'Gradient Boosting': {
                'model': GradientBoostingClassifier(random_state=42),
                'params': {
                        'n_estimators': [50, 100, 200], # Include original values
                        'learning_rate': [0.05, 0.1, 0.2],
                        'max_depth': [3, 4, 5], # Include original values
                        'subsample': [0.8, 1.0]
                }
        },
        'Support Vector Machine': {
                'model': SVC(probability=True, random_state=42),
                'params': {
                        'C': [0.1, 1, 10, 100],
                        'kernel': ['rbf', 'linear'],
                        'gamma': ['scale', 'auto', 0.1, 0.01],
                        'class_weight': [None, 'balanced']
                }
```

```
    },
    'Logistic Regression': {
        'model': LogisticRegression(random_state=42, max_iter=2000),
        'params': {
            'C': [0.1, 1, 10, 100],
            'solver': ['liblinear', 'lbfgs'],
            'penalty': ['l2'],
            'class_weight': [None, 'balanced']
        }
    },
    'K-Nearest Neighbors': {
        'model': KNeighborsClassifier(),
        'params': {
            'n_neighbors': [3, 5, 7, 9, 11],
            'weights': ['uniform', 'distance'],
            'p': [1, 2]
        }
    },

}

# Store model performance metrics
results = {
    'Model': [],
    'Accuracy': [], 'Precision': [], 'Recall': [], 'F1 Score': [],
    'AUC-ROC': [], 'Balanced Accuracy': [], 'Matthews Correlation Coefficient': [],
'Cross-validation Accuracy': []
}

# Store best parameters for each model
best_params_records = {
    'Model': [],
    'Best Parameters': [],
    'Best CV Score': [],
    'Parameter Grid': []
}

print("Starting training and evaluation of each model (GridSearchCV optimization + Ensemble
Learning)...")
print("=" * 80)

optimized_models = {}

# Train and evaluate each model
```

```
for model_name, mp in model_params.items():
    print(f"\nOptimizing and training {model_name}...")

    # Use GridSearchCV for hyperparameter optimization
    grid = GridSearchCV(mp['model'], mp['params'], cv=cv_strategy, scoring='accuracy',
n_jobs=-1)
    grid.fit(x_train_scaled, y_train)

    best_model = grid.best_estimator_
    optimized_models[model_name] = best_model

    print(f"    Best parameters: {grid.best_params_}")
    print(f"    Best CV score: {grid.best_score_:.4f}")

    # Record best parameters
    best_params_records['Model'].append(model_name)
    best_params_records['Best Parameters'].append(grid.best_params_)
    best_params_records['Best CV Score'].append(grid.best_score_)
    best_params_records['Parameter Grid'].append(mp['params'])

    # Predictions
    y_pred = best_model.predict(x_test_scaled)
    y_prob = best_model.predict_proba(x_test_scaled)

    # Calculate metrics
    accuracy = accuracy_score(y_test, y_pred)
    precision = precision_score(y_test, y_pred, average='binary', pos_label=2)
    recall = recall_score(y_test, y_pred, average='binary', pos_label=2)
    f1 = f1_score(y_test, y_pred, average='binary', pos_label=2)

    if hasattr(best_model, "predict_proba"):
        auc_roc = roc_auc_score(y_test, y_prob[:, 1])
    else:
        auc_roc = 0.0

    balanced_acc = balanced_accuracy_score(y_test, y_pred)
    mcc = matthews_corrcoef(y_test, y_pred)

    cv_scores = cross_val_score(best_model, x_train_scaled, y_train, cv=cv_strategy,
scoring='accuracy')
    cv_mean = cv_scores.mean()

    # Store results
    results['Model'].append(model_name)
```

```python
        results['Accuracy'].append(accuracy)
        results['Precision'].append(precision)
        results['Recall'].append(recall)
        results['F1 Score'].append(f1)
        results['AUC-ROC'].append(auc_roc)
        results['Balanced Accuracy'].append(balanced_acc)
        results['Matthews Correlation Coefficient'].append(mcc)
        results['Cross-validation Accuracy'].append(cv_mean)

        print(f"{model_name} optimized results: Acc={accuracy:.3f} | F1={f1:.3f} | AUC={auc_roc:.3f}")

# =================== Add Ensemble Model (Voting Classifier) ===================
print("\nTraining ensemble model (Soft Voting)...")

# 1. Sort models by CV scores
model_cv_scores = []
for name, model in optimized_models.items():
    idx = results['Model'].index(name)
    score = results['Cross-validation Accuracy'][idx]
    model_cv_scores.append((name, model, score))

# Sort in descending order
model_cv_scores.sort(key=lambda x: x[2], reverse=True)

# 2. Select Top 4 models (or all if less than 4)
top_n = 4
selected_models = model_cv_scores[:top_n]

print(f"\nTop {len(selected_models)} models for ensemble:")
estimators = []
weights = []
for name, model, score in selected_models:
    print(f"    {name}: CV Score = {score:.4f}")
    estimators.append((name, model))
    weights.append(score**2) # Use squared CV scores as weights to amplify differences

# 3. Build weighted VotingClassifier
print(f"Training weighted ensemble model (Weighted Soft Voting)...")
voting_clf = VotingClassifier(estimators=estimators, voting='soft', weights=weights)
voting_clf.fit(x_train_scaled, y_train)

y_pred_vote = voting_clf.predict(x_test_scaled)
y_prob_vote = voting_clf.predict_proba(x_test_scaled)
```

```
# Calculate ensemble model metrics
acc_vote = accuracy_score(y_test, y_pred_vote)
prec_vote = precision_score(y_test, y_pred_vote, average='binary', pos_label=2)
rec_vote = recall_score(y_test, y_pred_vote, average='binary', pos_label=2)
f1_vote = f1_score(y_test, y_pred_vote, average='binary', pos_label=2)
auc_vote = roc_auc_score(y_test, y_prob_vote[:, 1])
bal_acc_vote = balanced_accuracy_score(y_test, y_pred_vote)
mcc_vote = matthews_corrcoef(y_test, y_pred_vote)
cv_vote = cross_val_score(voting_clf, x_train_scaled, y_train, cv=cv_strategy, scoring='accuracy').mean()

# Add ensemble model results
results['Model'].append('Ensemble Model(Voting)')
results['Accuracy'].append(acc_vote)
results['Precision'].append(prec_vote)
results['Recall'].append(rec_vote)
results['F1 Score'].append(f1_vote)
results['AUC-ROC'].append(auc_vote)
results['Balanced Accuracy'].append(bal_acc_vote)
results['Matthews Correlation Coefficient'].append(mcc_vote)
results['Cross-validation Accuracy'].append(cv_vote)

print(f"Ensemble Model results: Acc={acc_vote:.3f} | F1={f1_vote:.3f} | AUC={auc_vote:.3f}")

# =================== Save Parameters to Table ===================
print("\n" + "=" * 80)
print("Best Parameters Records")
print("=" * 80)

# Create DataFrame for best parameters
best_params_df = pd.DataFrame(best_params_records)

# Format parameters for better readability
def format_params(params_dict):
    if isinstance(params_dict, dict):
        formatted = []
        for key, value in params_dict.items():
            formatted.append(f"{key}: {value}")
        return "\n".join(formatted)
    return str(params_dict)

best_params_df['Best Parameters (Formatted)'] = best_params_df['Best Parameters'].apply(format_params)
```

```
# Calculate total parameter combinations
def count_param_combinations(param_grid):
    total = 1
    for key, values in param_grid.items():
        total *= len(values) if isinstance(values, list) else 1
    return total

best_params_df['Parameter Combinations'] = best_params_df['Parameter Grid'].apply(count_param_combinations)
best_params_df['Best CV Score'] = best_params_df['Best CV Score'].round(4)

# Select columns for display
display_columns = ['Model', 'Best CV Score', 'Parameter Combinations', 'Best Parameters (Formatted)']
print(best_params_df[display_columns].to_string())

# Save to CSV
output_path = os.path.join(output_dir, '147_Best_Parameters_Table.csv')
best_params_df.to_csv(output_path, index=False)
print(f"\n✅ Best parameters table saved as: {output_path}")

# Save detailed parameters to JSON
output_json_path = os.path.join(output_dir, '147_Detailed_Parameters.json')
params_details = {
    'timestamp': datetime.now().strftime('%Y-%m-%d %H:%M:%S'),
    'data_info': {
        'dataset': '147.xlsx',
        'total_samples': data.shape[0],
        'training_samples': len(y_train),
        'test_samples': len(y_test),
        'features': list(data.columns[:-1])
    },
    'best_parameters': best_params_records,
    'ensemble_info': {
        'selected_models': [name for name, _, _ in selected_models],
        'weights': weights,
        'voting_method': 'soft'
    }
}

with open(output_json_path, 'w', encoding='utf-8') as f:
    json.dump(params_details, f, indent=2, ensure_ascii=False)
print(f"✅ Detailed parameters saved as: {output_json_path}")
```

```
# Create results DataFrame
results_df = pd.DataFrame(results)
results_df.set_index('Model', inplace=True)
print("\n" + "=" * 80)
print("Performance Comparison Summary (Optimized+Ensemble):")
print("=" * 80)
print(results_df.round(3))

# Find best model for each metric
print("\nBest models for each metric:")
for metric in ['Accuracy', 'Precision', 'Recall', 'F1 Score', 'AUC-ROC', 'Balanced Accuracy',
'Matthews Correlation Coefficient', 'Cross-validation Accuracy']:
    best_model = results_df[metric].idxmax()
    best_score = results_df[metric].max()
    print(f"{metric}: {best_model} ({best_score:.3f})")

# ==================== Generate separate charts and save to output directory
====================

# 1. Bar Chart - Comprehensive Performance Comparison
print("\nGenerating comprehensive performance bar chart...")
try:
    plt.figure(figsize=(14, 8))
    metrics_for_bar = ['Accuracy', 'Precision', 'Recall', 'F1 Score', 'AUC-ROC', 'Balanced
Accuracy']

    x = np.arange(len(results_df.index))
    width = 0.14
    colors = plt.cm.Set3(np.linspace(0, 1, len(metrics_for_bar)))

    ax = plt.subplot(111)

    for i, metric in enumerate(metrics_for_bar):
        offset = width * i - (width * (len(metrics_for_bar) - 1) / 2)
        values = results_df[metric].values
        bars = ax.bar(x + offset, values, width, label=metric, color=colors[i], alpha=0.8)

        for bar, value in zip(bars, values):
            height = bar.get_height()
            ax.text(bar.get_x() + bar.get_width()/2., height + 0.01,
                    f'{value:.3f}', ha='center', va='bottom', fontsize=4)

    ax.set_xlabel('Model')
```

```
    ax.set_ylabel('Score')
    ax.set_title('Comprehensive Performance Comparison of Models for 48-71 Months (Optimized+Ensemble)', size=14, fontweight='bold')
    ax.set_xticks(x)
    ax.set_xticklabels(results_df.index)
    ax.set_ylim(0, 1.05)
    ax.legend(title='Evaluation Metrics', bbox_to_anchor=(1.05, 1), loc='upper left')
    ax.grid(True, alpha=0.3, axis='y')

    plt.tight_layout()
    # Save as PDF in output directory
    output_path = os.path.join(output_dir, '147_Comprehensive_Performance_Bar_Chart.pdf')
    plt.savefig(output_path, dpi=300, bbox_inches='tight')
    print(f"✅ Saved as: {output_path}")
    plt.show()

    # 2. Bar Chart - AUC-ROC Comparison
    print("\nGenerating AUC-ROC comparison chart...")
    plt.figure(figsize=(10, 6))
    sorted_auc = results_df['AUC-ROC'].sort_values(ascending=True)
    colors = plt.cm.Set3(np.linspace(0, 1, len(sorted_auc)))
    bars = plt.barh(range(len(sorted_auc)), sorted_auc.values, color=colors)
    plt.yticks(range(len(sorted_auc)), sorted_auc.index)
    plt.xlabel('AUC-ROC')
    plt.title('AUC-ROC Comparison of Models (Optimized+Ensemble)')
    for i, bar in enumerate(bars):
        width = bar.get_width()
        plt.text(width + 0.01, bar.get_y() + bar.get_height()/2,
                 f'{width:.3f}', ha='left', va='center')
    plt.tight_layout()
    # Save as PDF in output directory
    output_path = os.path.join(output_dir, '147_AUC_ROC_Comparison_Chart.pdf')
    plt.savefig(output_path, dpi=300, bbox_inches='tight')
    print(f"✅ Saved as: {output_path}")
    plt.show()

    # ==================== Combined Chart: Subplot Format ====================

    print("\nGenerating combined comparison chart...")
    fig, (ax1, ax2) = plt.subplots(1, 2, figsize=(20, 9))

    # 1. Left subplot
    metrics_for_bar = ['Accuracy', 'Precision', 'Recall', 'F1 Score', 'AUC-ROC', 'Balanced Accuracy']
```

```
    x = np.arange(len(results_df.index))
    width = 0.13
    colors = plt.cm.Set3(np.linspace(0, 1, len(metrics_for_bar)))

    for i, metric in enumerate(metrics_for_bar):
        offset = width * i - (width * (len(metrics_for_bar) - 1) / 2)
        values = results_df[metric].values
        bars = ax1.bar(x + offset, values, width, label=metric, color=colors[i], alpha=0.8)

        for bar, value in zip(bars, values):
            height = bar.get_height()
            ax1.text(bar.get_x() + bar.get_width()/2., height + 0.01,
                    f'{value:.3f}', ha='center', va='bottom', fontsize=5)

    ax1.set_xlabel('Model', fontsize=11)
    ax1.set_ylabel('Score', fontsize=11)
    ax1.set_title('(C) Comprehensive Performance Comparison (Optimized+Ensemble)', size=14, fontweight='bold', pad=15)
    ax1.set_xticks(x)
    ax1.set_xticklabels(results_df.index, rotation=15)
    ax1.set_ylim(0, 1.05)
    ax1.legend(title='Evaluation Metrics', fontsize=8, title_fontsize=9)
    ax1.grid(True, alpha=0.3, axis='y')

    # 2. Right subplot
    sorted_auc = results_df['AUC-ROC'].sort_values(ascending=True)
    normalized_auc = (sorted_auc.values - sorted_auc.min()) / (sorted_auc.max() - sorted_auc.min() + 1e-8)
    colors_auc = plt.cm.Blues(normalized_auc * 0.7 + 0.3)

    bars = ax2.barh(range(len(sorted_auc)), sorted_auc.values, color=colors_auc, height=0.6)
    ax2.set_yticks(range(len(sorted_auc)))
    ax2.set_yticklabels(sorted_auc.index)
    ax2.set_xlabel('AUC-ROC Value', fontsize=11)
    ax2.set_title('(D) AUC-ROC Comparison of Models (Optimized+Ensemble)', size=14, fontweight='bold', pad=15)
    ax2.set_xlim(0, 1.05)

    for i, bar in enumerate(bars):
        width = bar.get_width()
        label_x = width + 0.01
        if label_x > 1.0:
            label_x = width - 0.02
            text_color = 'white'
```

```
        else:
            text_color = 'black'

        ax2.text(label_x, bar.get_y() + bar.get_height()/2,
                 f'{width:.3f}', ha='left', va='center', fontsize=10,
                 fontweight='bold', color=text_color)

    plt.suptitle('ASD Severity Prediction Model Performance for 48-71 Months (Optimized+Ensemble)', fontsize=18, fontweight='bold', y=0.98)
    plt.tight_layout(rect=[0, 0, 1, 0.95])
    # Save as PDF in output directory
    output_path = os.path.join(output_dir, '147_Combined_Comparison_Chart.pdf')
    plt.savefig(output_path, dpi=300, bbox_inches='tight')
    print(f"✅ Saved as: {output_path}")
    plt.show()

except Exception as e:
    print(f"Error during plotting: {e}")

# =================== Detailed Performance Comparison Table ====================
print("\n" + "=" * 100)
print("Detailed Performance Comparison Table (Suitable for Research Papers)")
print("=" * 100)

detailed_comparison = results_df.round(4)
detailed_comparison['Rank'] = detailed_comparison.mean(axis=1).rank(ascending=False).astype(int)
detailed_comparison = detailed_comparison.sort_values('Rank')

print("\nDetailed Model Performance Comparison (Sorted by Overall Rank):")
print(detailed_comparison)

# Save detailed comparison table as CSV
output_path = os.path.join(output_dir, '147_Detailed_Performance_Comparison.csv')
detailed_comparison.to_csv(output_path)
print(f"\n✅ Detailed performance comparison table saved as: {output_path}")

best_overall = detailed_comparison.index[0]
print(f"\n    Overall Recommended Best Model: {best_overall}")
print("Reason: Performs well and balanced across multiple evaluation metrics")

print("\n" + "=" * 80)
print("Optimized analysis completed!")
print(f"All results have been saved in the '{output_dir}' directory:")
```

```
print(f"1. 147_Best_Parameters_Table.csv - Best hyperparameters for each model")
print(f"2. 147_Detailed_Parameters.json - Detailed parameter information")
print(f"3. 147_Detailed_Performance_Comparison.csv - Performance metrics")
print(f"4. Various performance charts in PDF format")
print("=" * 80)
```
