# Supplementary material for: Research on the severity of symptoms in children with ASD based on integrated machine learning and structural equation modeling: age-specific predictive features and mediation effect path analysis
Source: Front Pediatr. 2026 Jun 17;14:1841816. doi: 10.3389/fped.2026.1841816 (PMC13318893; doi:10.3389/fped.2026.1841816)
Supplement: Supplementary file 11 [file Datasheet2.pdf]

Feature importance ranking code for the total sample + DeLong's test

```
# -*- coding: utf-8 -*-
"""
Multiple Machine Learning Algorithms Comparison with Feature Importance Analysis - ASD Severity Prediction (608 Cases)
Using Fixed Optimized Parameters from GridSearchCV Results
Added DeLong's Test for AUC Comparison
"""
import pandas as pd
import numpy as np
from sklearn.model_selection import train_test_split, cross_val_score, StratifiedKFold
from sklearn.preprocessing import StandardScaler
from sklearn.metrics import (accuracy_score, precision_score, recall_score,
                             f1_score, roc_auc_score, matthews_corrcoef,
                             balanced_accuracy_score, roc_curve)
import matplotlib.pyplot as plt
import seaborn as sns
from sklearn.ensemble import RandomForestClassifier, GradientBoostingClassifier, VotingClassifier
from sklearn.svm import SVC
from sklearn.linear_model import LogisticRegression
from sklearn.neighbors import KNeighborsClassifier
from sklearn.tree import DecisionTreeClassifier
from sklearn.inspection import permutation_importance
from scipy import stats
from scipy.stats import norm
import warnings
import os
import json
from datetime import datetime

warnings.filterwarnings('ignore')

# Set output directory
output_dir = "C:/Correlation between different first diagnosis ages and developmental levels"
if not os.path.exists(output_dir):
    os.makedirs(output_dir)

# Create feature importance subdirectory
feature_importance_dir = os.path.join(output_dir, "feature_importance")
if not os.path.exists(feature_importance_dir):
    os.makedirs(feature_importance_dir)

# Create DeLong test subdirectory
```

```
delong_dir = os.path.join(output_dir, "delong_test")
if not os.path.exists(delong_dir):
        os.makedirs(delong_dir)

# Set English font for plots
plt.rcParams['font.sans-serif'] = ['Arial', 'DejaVu Sans', 'sans-serif']
plt.rcParams['axes.unicode_minus'] = False
# Read data
data = pd.read_excel('C:/Correlation between different first diagnosis ages and developmental levels/608cases Python initial analysis (developmental level grading) version.xlsx',
  usecols=['age','HAZ', 'WAZ','BAZ','adaptive behavior', 'gross motor', 'fine motor', 'language', 'personal-social', 'Is_Severe'])

# Get feature names
feature_names = ['age','HAZ', 'WAZ', 'BAZ', 'adaptive behavior', 'gross motor', 'fine motor', 'language', 'personal-social']
print("Basic Information:")
print(f"Data shape: {data.shape}")
print("Features used:", feature_names)
print("Class distribution:")
print(data['Is_Severe'].value_counts())

# Split data
x_train, x_test, y_train, y_test = train_test_split(
        data.iloc[:,0:9], data.iloc[:,9], test_size=0.2, random_state=42)

# Standardize
scaler = StandardScaler()
x_train_scaled = scaler.fit_transform(x_train)
x_test_scaled = scaler.transform(x_test)

# =================== Data Augmentation Strategy ===================
print("\nApplying data augmentation strategies...")
try:
        from imblearn.over_sampling import SMOTE
        # Automatically adjust k_neighbors to prevent small sample errors
        min_samples = y_train.value_counts().min()
        k_neighbors = min(5, min_samples - 1) if min_samples > 1 else 1

        smote = SMOTE(random_state=42, k_neighbors=k_neighbors)
        x_train_scaled, y_train = smote.fit_resample(x_train_scaled, y_train)
        print(f" ✅ SMOTE data augmentation successful! Training samples increased from {len(x_train)} to {len(x_train_scaled)}")
except ImportError:
```

```
        print("    imblearn library not detected, switching to random oversampling...")
        # Manual simple oversampling implementation
        y_train_np = y_train.values if hasattr(y_train, 'values') else y_train
        classes, counts = np.unique(y_train_np, return_counts=True)
        max_count = counts.max()

        x_resampled = []
        y_resampled = []

        for cls in classes:
            cls_idx = np.where(y_train_np == cls)[0]
            x_cls = x_train_scaled[cls_idx]

            if len(x_cls) < max_count:
                indices = np.random.choice(len(x_cls), max_count, replace=True)
                x_cls = x_cls[indices]

            x_resampled.append(x_cls)
            y_resampled.append(np.full(max_count, cls))

        x_train_scaled = np.vstack(x_resampled)
        y_train = np.concatenate(y_resampled)
        print(f"☑ Random oversampling successful! Training samples increased to {len(y_train)}")
except Exception as e:
    print(f"✖ Data augmentation failed: {e}, continuing with original data...")

# Cross-validation strategy
cv_strategy = StratifiedKFold(n_splits=5, shuffle=True, random_state=42)

# ==================== Define Models with Fixed Optimized Parameters
====================
print("\n" + "=" * 80)
print("Initializing models with fixed optimized parameters...")
print("=" * 80)

# Fixed optimized parameters from GridSearchCV results (608 cases)
fixed_optimized_models = {
    'Random Forest': RandomForestClassifier(
        class_weight='balanced',    # 608 cases parameter: balanced
        max_depth=20,               # 608 cases parameter: 20
        max_features='sqrt',        # 608 cases parameter: sqrt
        min_samples_leaf=1,         # 608 cases parameter: 1
        min_samples_split=2,        # 608 cases parameter: 2
        n_estimators=200,           # 608 cases parameter: 200
```

```
            random_state=42,
            n_jobs=-1
        ),
        'Gradient Boosting': GradientBoostingClassifier(
            learning_rate=0.05,           # 608 cases parameter: 0.05
            max_depth=5,                  # 608 cases parameter: 5
            n_estimators=200,             # 608 cases parameter: 200
            subsample=0.8,                # 608 cases parameter: 0.8
            random_state=42
        ),
        'Support Vector Machine': SVC(
            C=10,                         # 608 cases parameter: 10
            class_weight='balanced', # 608 cases parameter: balanced
            gamma=0.1,                    # 608 cases parameter: 0.1
            kernel='rbf',                 # 608 cases parameter: rbf
            probability=True,
            random_state=42
        ),
        'Logistic Regression': LogisticRegression(
            C=0.1,                        # 608 cases parameter: 0.1
            class_weight=None,            # 608 cases parameter: None
            penalty='l2',                 # 608 cases parameter: l2
            solver='liblinear',           # 608 cases parameter: liblinear
            max_iter=2000,
            random_state=42
        ),
        'K-Nearest Neighbors': KNeighborsClassifier(
            n_neighbors=11,               # 608 cases parameter: 11
            p=1,                          # 608 cases parameter: 1
            weights='distance'            # 608 cases parameter: distance
        ),
}

# =================== DeLong's Test Function ===================
def delong_roc_variance(ground_truth, predictions):
        """
        Calculate the variance of the AUC using DeLong's method
        """
        # Get positive and negative samples
        pos_indices = np.where(ground_truth == 2)[0]    # Assuming 2 is positive class
        neg_indices = np.where(ground_truth == 1)[0]    # Assuming 1 is negative class

        n_pos = len(pos_indices)
        n_neg = len(neg_indices)
```

```
    if n_pos == 0 or n_neg == 0:
        return 0, 0

    # Create the structural components
    v10 = np.zeros((len(ground_truth),))
    v01 = np.zeros((len(ground_truth),))

    for i in range(n_pos):
        for j in range(n_neg):
            if predictions[pos_indices[i]] > predictions[neg_indices[j]]:
                v10[pos_indices[i]] += 1
            elif predictions[pos_indices[i]] < predictions[neg_indices[j]]:
                v01[pos_indices[i]] += 1

    v10 = v10 / n_neg
    v01 = v01 / n_pos

    # Calculate variance
    var_pos = np.var(v10[pos_indices]) / n_pos
    var_neg = np.var(v01[neg_indices]) / n_neg

    return var_pos + var_neg, var_pos, var_neg


def delong_roc_test(ground_truth, pred1, pred2):
    """
    DeLong's test for comparing two AUCs
    """
    # Calculate AUC for both predictions
    auc1 = roc_auc_score(ground_truth, pred1)
    auc2 = roc_auc_score(ground_truth, pred2)

    # Calculate variances
    var1, v1_pos, v1_neg = delong_roc_variance(ground_truth, pred1)
    var2, v2_pos, v2_neg = delong_roc_variance(ground_truth, pred2)

    # Calculate covariance
    pos_indices = np.where(ground_truth == 2)[0]
    neg_indices = np.where(ground_truth == 1)[0]

    n_pos = len(pos_indices)
    n_neg = len(neg_indices)
```

```
    if n_pos == 0 or n_neg == 0:
        return auc1, auc2, 0, 1.0  # Return p-value=1.0 if can't calculate

    # Create structural components for both models
    v10_1 = np.zeros((len(ground_truth),))
    v10_2 = np.zeros((len(ground_truth),))
    v01_1 = np.zeros((len(ground_truth),))
    v01_2 = np.zeros((len(ground_truth),))

    for i in range(n_pos):
        for j in range(n_neg):
            # Model 1
            if pred1[pos_indices[i]] > pred1[neg_indices[j]]:
                v10_1[pos_indices[i]] += 1
            elif pred1[pos_indices[i]] < pred1[neg_indices[j]]:
                v01_1[pos_indices[i]] += 1

            # Model 2
            if pred2[pos_indices[i]] > pred2[neg_indices[j]]:
                v10_2[pos_indices[i]] += 1
            elif pred2[pos_indices[i]] < pred2[neg_indices[j]]:
                v01_2[pos_indices[i]] += 1

    v10_1 = v10_1 / n_neg
    v10_2 = v10_2 / n_neg
    v01_1 = v01_1 / n_pos
    v01_2 = v01_2 / n_pos

    # Calculate covariance
    cov_pos = np.mean(v10_1[pos_indices] * v10_2[pos_indices]) - np.mean(v10_1[pos_indices]) * np.mean(v10_2[pos_indices])
    cov_neg = np.mean(v01_1[neg_indices] * v01_2[neg_indices]) - np.mean(v01_1[neg_indices]) * np.mean(v01_2[neg_indices])

    cov = cov_pos / n_pos + cov_neg / n_neg

    # Calculate z-score
    if var1 + var2 - 2 * cov <= 0:
        z_score = 0
        p_value = 1.0
    else:
        z_score = (auc1 - auc2) / np.sqrt(var1 + var2 - 2 * cov)
        p_value = 2 * (1 - norm.cdf(np.abs(z_score)))
```

```
        return auc1, auc2, z_score, p_value


# Store model performance metrics
results = {
        'Model': [],
        'Accuracy': [], 'Precision': [], 'Recall': [], 'F1 Score': [],
        'AUC-ROC': [], 'Balanced Accuracy': [], 'Matthews Correlation Coefficient': [],
'Cross-validation Accuracy': []
}

# Store model predictions for DeLong test
model_probabilities = {}

# Store feature importance results
feature_importance_results = {
        'Model': [],
        'Features': [],
        'Importance_Values': [],
        'Importance_Rank': [],
        'Top_3_Features': [],
        'Top_3_Values': []
}

# Store best parameter records
best_params_records = {
        'Model': [],
        'Best Parameters': [],
        'Best CV Score': [],
        'Search_Space_Size': []
}

# Fixed best CV scores (608 cases)
fixed_best_cv_scores = {
        'Random Forest': 0.7208,
        'Gradient Boosting': 0.7208,
        'Support Vector Machine': 0.6887,
        'Logistic Regression': 0.6566,
        'K-Nearest Neighbors': 0.6943,
}

# Search space sizes (608 cases)
search_space_sizes = {
        'Random Forest': 180,
```

```
    'Gradient Boosting': 54,
    'Support Vector Machine': 64,
    'Logistic Regression': 16,
    'K-Nearest Neighbors': 20,
}

# Store best parameters
for model_name in fixed_optimized_models.keys():
    best_params_records['Model'].append(model_name)
    best_params_records['Best CV Score'].append(fixed_best_cv_scores.get(model_name, 0))
    best_params_records['Search_Space_Size'].append(search_space_sizes.get(model_name,
0))

    if model_name == 'Random Forest':
        best_params_records['Best Parameters'].append({
            'class_weight': 'balanced',
            'max_depth': 20,
            'max_features': 'sqrt',
            'min_samples_leaf': 1,
            'min_samples_split': 2,
            'n_estimators': 200
        })
    elif model_name == 'Gradient Boosting':
        best_params_records['Best Parameters'].append({
            'learning_rate': 0.05,
            'max_depth': 5,
            'n_estimators': 200,
            'subsample': 0.8
        })
    elif model_name == 'Support Vector Machine':
        best_params_records['Best Parameters'].append({
            'C': 10,
            'class_weight': 'balanced',
            'gamma': 0.1,
            'kernel': 'rbf'
        })
    elif model_name == 'Logistic Regression':
        best_params_records['Best Parameters'].append({
            'C': 0.1,
            'class_weight': None,
            'penalty': 'l2',
            'solver': 'liblinear'
        })
    elif model_name == 'K-Nearest Neighbors':
```

```
            best_params_records['Best Parameters'].append({
                'n_neighbors': 11,
                'p': 1,
                'weights': 'distance'
            })


print("\n" + "=" * 80)
print("Starting training and evaluation for each model with fixed optimized parameters...")
print("=" * 80)

# Train and evaluate each model
for model_name, model in fixed_optimized_models.items():
    print(f"\nTraining {model_name} with fixed optimized parameters...")

    # Train model
    model.fit(x_train_scaled, y_train)

    # Cross-validation scores (recalculated)
    cv_scores = cross_val_score(model, x_train_scaled, y_train, cv=cv_strategy,
scoring='accuracy')
    cv_mean = cv_scores.mean()

    # Predictions
    y_pred = model.predict(x_test_scaled)
    y_prob = model.predict_proba(x_test_scaled) if hasattr(model, "predict_proba") else None

    # Store probabilities for DeLong test
    if y_prob is not None:
        model_probabilities[model_name] = y_prob[:, 1]    # Store probability of positive class

    # Calculate metrics
    accuracy = accuracy_score(y_test, y_pred)
    precision = precision_score(y_test, y_pred, average='binary', pos_label=2)
    recall = recall_score(y_test, y_pred, average='binary', pos_label=2)
    f1 = f1_score(y_test, y_pred, average='binary', pos_label=2)

    if y_prob is not None:
        auc_roc = roc_auc_score(y_test, y_prob[:, 1])
    else:
        auc_roc = 0.0

    balanced_acc = balanced_accuracy_score(y_test, y_pred)
    mcc = matthews_corrcoef(y_test, y_pred)
```

```
        # Store results
        results['Model'].append(model_name)
        results['Accuracy'].append(accuracy)
        results['Precision'].append(precision)
        results['Recall'].append(recall)
        results['F1 Score'].append(f1)
        results['AUC-ROC'].append(auc_roc)
        results['Balanced Accuracy'].append(balanced_acc)
        results['Matthews Correlation Coefficient'].append(mcc)
        results['Cross-validation Accuracy'].append(cv_mean)

        print(f"{model_name}  results:  Accuracy={accuracy:.3f}  |  F1={f1:.3f}  |  AUC={auc_roc:.3f}  |
Cross-validation={cv_mean:.3f}")

        # =================== Feature Importance Analysis ===================
        print(f"    Calculating feature importance for {model_name}...")

        try:
            if model_name in ['Random Forest', 'Gradient Boosting', 'Decision Tree']:
                # Tree-based models have built-in feature importance
                if hasattr(model, 'feature_importances_'):
                    importance = model.feature_importances_
                    importance_type = "Gini Importance"
                else:
                    importance = np.zeros(len(feature_names))
                    importance_type = "Unavailable"

            elif model_name == 'Logistic Regression':
                # Logistic Regression uses coefficients
                if hasattr(model, 'coef_'):
                    importance = np.abs(model.coef_[0])
                    importance_type = "Absolute Coefficient"
                else:
                    importance = np.zeros(len(feature_names))
                    importance_type = "Unavailable"

            elif model_name == 'Support Vector Machine':
                # For non-linear SVM, use permutation importance
                try:
                    perm_importance = permutation_importance(
                        model,     x_test_scaled,     y_test,     n_repeats=10,     random_state=42,
n_jobs=-1
                    )
```

```
                importance = np.abs(perm_importance.importances_mean)
                importance_type = "Permutation Importance"
            except:
                importance = np.zeros(len(feature_names))
                importance_type = "Unavailable"

        elif model_name == 'K-Nearest Neighbors':
            # KNN has no built-in importance, use permutation importance
            try:
                perm_importance = permutation_importance(
                    model, x_test_scaled, y_test, n_repeats=10, random_state=42,
n_jobs=-1
                )
                importance = np.abs(perm_importance.importances_mean)
                importance_type = "Permutation Importance"
            except:
                importance = np.zeros(len(feature_names))
                importance_type = "Unavailable"
        else:
            importance = np.zeros(len(feature_names))
            importance_type = "Unavailable"

        # Normalize importance to sum to 1
        if np.sum(importance) > 0:
            importance_normalized = importance / np.sum(importance)
        else:
            importance_normalized = importance

        # Create ranking
        ranked_indices = np.argsort(importance_normalized)[::-1]
        ranked_features = [feature_names[i] for i in ranked_indices]
        ranked_values = importance_normalized[ranked_indices]

        # Get top 3 features
        top_3_features = ranked_features[:3] if len(ranked_features) >= 3 else ranked_features
        top_3_values = ranked_values[:3] if len(ranked_values) >= 3 else ranked_values

        # Store feature importance results
        feature_importance_results['Model'].append(model_name)
        feature_importance_results['Features'].append(ranked_features)
        feature_importance_results['Importance_Values'].append(ranked_values)
        feature_importance_results['Importance_Rank'].append(list(range(1,
len(ranked_features) + 1)))
        feature_importance_results['Top_3_Features'].append(top_3_features)
```

```
            feature_importance_results['Top_3_Values'].append(top_3_values)

            print(f"    Importance type: {importance_type}")
            print(f"     Top 3 features: {', '.join([f'{feat}({val:.4f})' for feat, val in zip(top_3_features,
top_3_values)])}")

        except Exception as e:
            print(f"    Error calculating feature importance: {e}")
            # Add default values
            feature_importance_results['Model'].append(model_name)
            feature_importance_results['Features'].append(feature_names)

feature_importance_results['Importance_Values'].append(np.zeros(len(feature_names)))
            feature_importance_results['Importance_Rank'].append(list(range(1,
len(feature_names) + 1)))
            feature_importance_results['Top_3_Features'].append([])
            feature_importance_results['Top_3_Values'].append([])

# =================== Add Ensemble Model (Voting Classifier) ===================
print("\n" + "=" * 80)
print("Training ensemble model (soft voting)...")

# 1. Sort models by CV score
model_cv_scores = []
for name, model in fixed_optimized_models.items():
    idx = results['Model'].index(name)
    score = results['Cross-validation Accuracy'][idx]
    model_cv_scores.append((name, model, score))

# Sort in descending order
model_cv_scores.sort(key=lambda x: x[2], reverse=True)

# 2. Select top 4 models
top_n = min(4, len(model_cv_scores))
selected_models = model_cv_scores[:top_n]

print(f"\nTop {len(selected_models)} models for ensemble:")
estimators = []
weights = []
for name, model, score in selected_models:
    print(f"    {name}: CV score = {score:.4f}")
    estimators.append((name, model))
    weights.append(score)    # Use CV score as weight
```

```
# 3. Build weighted VotingClassifier
print(f"Training weighted ensemble model (weighted soft voting)...")
voting_clf = VotingClassifier(estimators=estimators, voting='soft', weights=weights)
voting_clf.fit(x_train_scaled, y_train)

y_pred_vote = voting_clf.predict(x_test_scaled)
y_prob_vote = voting_clf.predict_proba(x_test_scaled)

# Store probabilities for DeLong test
model_probabilities['Ensemble Model'] = y_prob_vote[:, 1]

# Calculate ensemble model metrics
acc_vote = accuracy_score(y_test, y_pred_vote)
prec_vote = precision_score(y_test, y_pred_vote, average='binary', pos_label=2)
rec_vote = recall_score(y_test, y_pred_vote, average='binary', pos_label=2)
f1_vote = f1_score(y_test, y_pred_vote, average='binary', pos_label=2)
auc_vote = roc_auc_score(y_test, y_prob_vote[:, 1])
bal_acc_vote = balanced_accuracy_score(y_test, y_pred_vote)
mcc_vote = matthews_corrcoef(y_test, y_pred_vote)
cv_vote = cross_val_score(voting_clf, x_train_scaled, y_train, cv=cv_strategy,
scoring='accuracy').mean()

# Add ensemble model results
results['Model'].append('Ensemble Model')
results['Accuracy'].append(acc_vote)
results['Precision'].append(prec_vote)
results['Recall'].append(rec_vote)
results['F1 Score'].append(f1_vote)
results['AUC-ROC'].append(auc_vote)
results['Balanced Accuracy'].append(bal_acc_vote)
results['Matthews Correlation Coefficient'].append(mcc_vote)
results['Cross-validation Accuracy'].append(cv_vote)

print(f"Ensemble model results: Accuracy={acc_vote:.3f} | F1={f1_vote:.3f} |
AUC={auc_vote:.3f}")

# =================== DeLong's Test Analysis ===================
print("\n" + "=" * 80)
print("Performing DeLong's Test for AUC Comparison...")
print("=" * 80)

# Create results for DeLong test
delong_results = {
    'Model 1': [],
```

```
    'Model 2': [],
    'AUC 1': [],
    'AUC 2': [],
    'Z-Score': [],
    'P-Value': [],
    'Significant (p<0.05)': [],
    'Significant (p<0.01)': [],
    'Significant (p<0.001)': []
}

# Get all model names that have probability predictions
model_names_with_probs = list(model_probabilities.keys())

print(f"\nTotal {len(model_names_with_probs)} models will be compared:")
for i, name in enumerate(model_names_with_probs):
    auc = roc_auc_score(y_test, model_probabilities[name])
    print(f"    {i+1}. {name}: AUC = {auc:.4f}")

print(f"\nTotal pairwise comparisons: {len(model_names_with_probs) *
(len(model_names_with_probs) - 1) // 2}")

# Perform pairwise comparisons
comparison_count = 0
significant_count_05 = 0
significant_count_01 = 0
significant_count_001 = 0

for i in range(len(model_names_with_probs)):
    for j in range(i + 1, len(model_names_with_probs)):
        model1_name = model_names_with_probs[i]
        model2_name = model_names_with_probs[j]

        prob1 = model_probabilities[model1_name]
        prob2 = model_probabilities[model2_name]

        # Perform DeLong's test
        auc1, auc2, z_score, p_value = delong_roc_test(y_test.values, prob1, prob2)

        # Store results
        delong_results['Model 1'].append(model1_name)
        delong_results['Model 2'].append(model2_name)
        delong_results['AUC 1'].append(auc1)
        delong_results['AUC 2'].append(auc2)
        delong_results['Z-Score'].append(z_score)
```

```
        delong_results['P-Value'].append(p_value)
        delong_results['Significant (p<0.05)'].append('Yes' if p_value < 0.05 else 'No')
        delong_results['Significant (p<0.01)'].append('Yes' if p_value < 0.01 else 'No')
        delong_results['Significant (p<0.001)'].append('Yes' if p_value < 0.001 else 'No')

        comparison_count += 1

        if p_value < 0.05:
            significant_count_05 += 1
            if p_value < 0.01:
                significant_count_01 += 1
                if p_value < 0.001:
                    significant_count_001 += 1

        # Print results
        significance_marker = ""
        if p_value < 0.001:
            significance_marker = " ***"
        elif p_value < 0.01:
            significance_marker = " **"
        elif p_value < 0.05:
            significance_marker = " *"

        print(f"    {model1_name} vs {model2_name}:")
        print(f"        AUC: {auc1:.4f} vs {auc2:.4f},   ΔAUC = {auc1 - auc2:.4f}")
        print(f"        Z-score = {z_score:.4f}, P-value = {p_value:.6f}{significance_marker}")

# Create DeLong test results DataFrame
delong_df = pd.DataFrame(delong_results)

# Save DeLong test results
delong_output_path = os.path.join(delong_dir, '608_Delong_Test_Results.csv')
delong_df.to_csv(delong_output_path, index=False, encoding='utf-8-sig')
print(f"\n✅ DeLong's test results saved as: {delong_output_path}")

# ==================== Create DeLong Test Summary ====================
print("\n" + "=" * 80)
print("DeLong's Test Summary")
print("=" * 80)

print(f"\nTotal pairwise comparisons: {comparison_count}")
print(f"Significant at p<0.05: {significant_count_05}
({significant_count_05/comparison_count*100:.1f}%)")
print(f"Significant at p<0.01: {significant_count_01}
```

```
({significant_count_01/comparison_count*100:.1f}%)")
print(f"Significant at p<0.001: {significant_count_001}
({significant_count_001/comparison_count*100:.1f}%)")

# Find the best model based on AUC
best_auc_model = max(model_probabilities.keys(), key=lambda x: roc_auc_score(y_test,
model_probabilities[x]))
best_auc = roc_auc_score(y_test, model_probabilities[best_auc_model])

print(f"\nBest performing model based on AUC: {best_auc_model} (AUC = {best_auc:.4f})")

# Check if the best model is significantly better than others
print(f"\nComparisons with {best_auc_model}:")
best_comparisons = delong_df[(delong_df['Model 1'] == best_auc_model) | (delong_df['Model 2']
== best_auc_model)]
for idx, row in best_comparisons.iterrows():
    if row['Model 1'] == best_auc_model:
        other_model = row['Model 2']
    else:
        other_model = row['Model 1']

    significance = ""
    if row['P-Value'] < 0.001:
        significance = "***"
    elif row['P-Value'] < 0.01:
        significance = "**"
    elif row['P-Value'] < 0.05:
        significance = "*"

    print(f"    vs {other_model}: P-value = {row['P-Value']:.6f}{significance}")

# =================== Create DeLong Test Visualization (Only Heatmap)
===================
print("\n" + "=" * 80)
print("Creating DeLong's Test visualization (P-value Heatmap)...")
print("=" * 80)

try:
    # Create single heatmap of p-values
    fig, ax = plt.subplots(1, 1, figsize=(12, 10))

    n_models = len(model_names_sorted) if 'model_names_sorted' in locals() else
len(model_names_with_probs)
```

```
    # Sort model names if not already sorted
    if 'model_names_sorted' not in locals():
        model_names_sorted = sorted(model_names_with_probs)

    p_value_matrix = np.zeros((n_models, n_models))

    for i in range(n_models):
        for j in range(n_models):
            if i == j:
                p_value_matrix[i, j] = 1.0
            else:
                model1 = model_names_sorted[i]
                model2 = model_names_sorted[j]

                # Find the comparison
                for idx, row in delong_df.iterrows():
                    if (row['Model 1'] == model1 and row['Model 2'] == model2) or \
                       (row['Model 1'] == model2 and row['Model 2'] == model1):
                        p_value_matrix[i, j] = row['P-Value']
                        break

    # Create heatmap
    im = ax.imshow(p_value_matrix, cmap='RdYlGn_r', vmin=0, vmax=1)
    ax.set_xticks(range(n_models))
    ax.set_yticks(range(n_models))
    ax.set_xticklabels(model_names_sorted, rotation=45, ha='right', fontsize=10)
    ax.set_yticklabels(model_names_sorted, fontsize=10)
    ax.set_title('(A) DeLong Test P-Value Matrix for AUC Comparison', fontsize=14,
fontweight='bold', pad=15)

    # Add text annotations
    for i in range(n_models):
        for j in range(n_models):
            if i == j:
                text = '1.0000\n(self)'
                text_color = 'black'
            else:
                p_val = p_value_matrix[i, j]
                text = f'{p_val:.4f}'
                if p_val < 0.05:
                    text += '\n*'
                    if p_val < 0.01:
                        text += '*'
                        if p_val < 0.001:
```

```
                        text += '*'

                    text_color = 'white' if p_val < 0.3 or p_val > 0.7 else 'black'

                ax.text(j, i, text, ha='center', va='center', fontsize=9, color=text_color,
fontweight='bold')

        # Add colorbar
        cbar = plt.colorbar(im, ax=ax, shrink=0.8)
        cbar.set_label('P-Value', fontsize=11)

        # Add significance legend
        ax.text(0.02, -0.15, '* p < 0.05    ** p < 0.01    *** p < 0.001',
                transform=ax.transAxes, fontsize=9, style='italic')

        plt.tight_layout()
        delong_plot_path = os.path.join(delong_dir, '608_Delong_Test_Heatmap.tiff')
        plt.savefig(delong_plot_path, dpi=600, bbox_inches='tight')
        print(f"✅ DeLong's test P-value heatmap saved as: {delong_plot_path}")
        plt.show()

except Exception as e:
    print(f"Error creating DeLong test visualization: {e}")

# =================== Add feature importance for ensemble model ===================
print("\nCalculating feature importance for ensemble model...")
try:
    ensemble_importance = np.zeros(len(feature_names))
    total_weight = 0

    for (name, _), weight in zip(estimators, weights):
        # Find this model in feature_importance_results
        model_idx = None
        for i, model_name in enumerate(feature_importance_results['Model']):
            if model_name == name:
                model_idx = i
                break

        if model_idx is not None:
            # Get importance values for this model
            importance_values                                                    =
feature_importance_results['Importance_Values'][model_idx]
            # Reorder to original feature order
            features_order = feature_importance_results['Features'][model_idx]
```

```
                importance_dict = dict(zip(features_order, importance_values))

                # Convert to original order
                ordered_importance = np.array([importance_dict.get(feat, 0) for feat in
feature_names])
                ensemble_importance += ordered_importance * weight
                total_weight += weight

    if total_weight > 0:
        ensemble_importance = ensemble_importance / total_weight
        # Normalize
        if np.sum(ensemble_importance) > 0:
            ensemble_importance = ensemble_importance / np.sum(ensemble_importance)

    # Add feature importance results for ensemble model
    ranked_indices = np.argsort(ensemble_importance)[::-1]
    ranked_features = [feature_names[i] for i in ranked_indices]
    ranked_values = ensemble_importance[ranked_indices]
    top_3_features = ranked_features[:3] if len(ranked_features) >= 3 else ranked_features
    top_3_values = ranked_values[:3] if len(ranked_values) >= 3 else ranked_values

    feature_importance_results['Model'].append('Ensemble Model')
    feature_importance_results['Features'].append(ranked_features)
    feature_importance_results['Importance_Values'].append(ranked_values)
    feature_importance_results['Importance_Rank'].append(list(range(1, len(ranked_features) +
1)))
    feature_importance_results['Top_3_Features'].append(top_3_features)
    feature_importance_results['Top_3_Values'].append(top_3_values)

    print(f"Ensemble model top 3 features: {', '.join([f'{feat}({val:.4f})' for feat, val in
zip(top_3_features, top_3_values)])}")

except Exception as e:
    print(f"Error calculating ensemble model feature importance: {e}")

# ==================== Create Feature Importance DataFrame ====================
print("\n" + "=" * 80)
print("Creating Feature Importance DataFrame...")
print("=" * 80)

# Create feature importance DataFrame
feature_importance_df = pd.DataFrame(feature_importance_results)
# Save feature importance results
feature_importance_output_path = os.path.join(feature_importance_dir,
```

```
'608_Feature_Importance_Summary.csv')
feature_importance_df.to_csv(feature_importance_output_path, index=False, encoding='utf-8-sig')
print(f"✅ Feature importance summary saved as: {feature_importance_output_path}")

# =================== Calculate Overall Feature Importance ===================
print("\nCalculating overall feature importance...")

# Create importance matrix for each model
importance_matrix = []
model_names = feature_importance_df['Model'].tolist()

for i, model_name in enumerate(model_names):
    importance_dict = dict(zip(feature_importance_df['Features'].iloc[i],
                               feature_importance_df['Importance_Values'].iloc[i]))

    # Convert to original feature order
    row = [importance_dict.get(feat, 0) for feat in feature_names]
    importance_matrix.append(row)

importance_matrix = np.array(importance_matrix)

# Calculate average importance for each feature (considering only non-zero values)
overall_feature_importance = {}
for j, feat in enumerate(feature_names):
    # Get importance values for this feature across all models
    feat_importances = importance_matrix[:, j]
    # Consider only non-zero values
    non_zero_importances = feat_importances[feat_importances > 0]

    if len(non_zero_importances) > 0:
        overall_feature_importance[feat] = np.mean(non_zero_importances)
    else:
        overall_feature_importance[feat] = 0

# Normalize overall importance to sum to 1
total_importance = sum(overall_feature_importance.values())
if total_importance > 0:
    overall_feature_importance = {k: v/total_importance for k, v in overall_feature_importance.items()}

# Sort by importance
overall_feature_importance_sorted = dict(sorted(overall_feature_importance.items(),
                                                key=lambda x: x[1], reverse=True))
```

```
# Save overall feature importance
overall_importance_df = pd.DataFrame(list(overall_feature_importance_sorted.items()),
                                     columns=['Feature', 'Overall_Importance'])
overall_importance_output_path = os.path.join(feature_importance_dir,
'608_Overall_Feature_Importance.csv')
overall_importance_df.to_csv(overall_importance_output_path, index=False,
encoding='utf-8-sig')
print(f"✅ Overall feature importance saved as: {overall_importance_output_path}")

# ==================== Create Results DataFrame ====================
results_df = pd.DataFrame(results)
results_df.set_index('Model', inplace=True)

# Save performance results
performance_output_path = os.path.join(output_dir, '608_Performance_Results.csv')
results_df.to_csv(performance_output_path, encoding='utf-8-sig')
print(f"✅ Performance results saved as: {performance_output_path}")


# ==================== Generate Algorithm Model Comparison Combined Plot
====================
print("\n" + "=" * 80)
print("Generating algorithm model comparison combined plot...")
print("=" * 80)

try:
    # Create combined plot (for publication)
    fig, (ax1, ax2) = plt.subplots(1, 2, figsize=(20, 9))

    # 1. Left subplot - Comprehensive performance comparison
    metrics_for_bar = ['Accuracy', 'Precision', 'Recall', 'F1 Score', 'AUC-ROC', 'Balanced
Accuracy']
    x = np.arange(len(results_df.index))
    width = 0.13
    colors = plt.cm.Set3(np.linspace(0, 1, len(metrics_for_bar)))

    for i, metric in enumerate(metrics_for_bar):
        offset = width * i - (width * (len(metrics_for_bar) - 1) / 2)
        values = results_df[metric].values
        bars = ax1.bar(x + offset, values, width, label=metric, color=colors[i], alpha=0.8)

        for bar, value in zip(bars, values):
            height = bar.get_height()
```

```
                # All numbers above bars are uniformly black, placed above the bars
                ax1.text(bar.get_x() + bar.get_width()/2., height + 0.01,
                        f'{value:.3f}', ha='center', va='bottom', fontsize=5, color='black',
fontweight='bold')

    ax1.set_xlabel('Model', fontsize=11)
    ax1.set_ylabel('Score', fontsize=11)
    ax1.set_title('(A) Performance of different machine models on the testing database', size=14,
fontweight='bold', pad=15)
    ax1.set_xticks(x)
    ax1.set_xticklabels(results_df.index, rotation=15)
    ax1.set_ylim(0, 1.05)
    # Legend uniformly on the right inside the box
    ax1.legend(title='Evaluation Metrics', fontsize=8, title_fontsize=9, loc='upper right')
    ax1.grid(True, alpha=0.3, axis='y')

    # 2. Right subplot - AUC-ROC comparison
    sorted_auc = results_df['AUC-ROC'].sort_values(ascending=True)
    normalized_auc = (sorted_auc.values - sorted_auc.min()) / (sorted_auc.max() -
sorted_auc.min() + 1e-8)
    colors_auc = plt.cm.Blues(normalized_auc * 0.7 + 0.3)

    bars = ax2.barh(range(len(sorted_auc)), sorted_auc.values, color=colors_auc, height=0.6)
    ax2.set_yticks(range(len(sorted_auc)))
    ax2.set_yticklabels(sorted_auc.index)
    ax2.set_xlabel('AUC-ROC Value', fontsize=11)
    ax2.set_title('(B)', size=14, fontweight='bold', pad=15)
    ax2.set_xlim(0, 1.05)

    for i, bar in enumerate(bars):
        width = bar.get_width()
        label_x = width + 0.01
        if label_x > 1.0:
            label_x = width - 0.02
            text_color = 'white'
        else:
            text_color = 'black'

        ax2.text(label_x, bar.get_y() + bar.get_height()/2,
                f'{width:.3f}', ha='left', va='center', fontsize=10,
                fontweight='bold', color=text_color)
    fig.tight_layout()    # Automatically adjust layout, usually solves issues
    # Save as TIFF format
    output_path = os.path.join(output_dir, '608_Algorithm_Comparison_Combined.tiff')
```

```
    plt.savefig(output_path, dpi=600, bbox_inches='tight')
    print(f"☑ Algorithm model comparison combined plot saved as: {output_path}")
    plt.show()

except Exception as e:
    print(f"Error generating combined plot: {e}")


# =================== Generate Feature Importance Visualization ===================
print("\n" + "=" * 80)
print("Generating feature importance visualization...")
print("=" * 80)


try:
    # 1. Overall feature importance plot
    print("\n1. Generating overall feature importance plot...")
    plt.figure(figsize=(12, 8))

    features = list(overall_feature_importance_sorted.keys())
    importance_values = list(overall_feature_importance_sorted.values())

    colors = plt.cm.viridis(np.linspace(0.3, 0.9, len(features)))
    bars = plt.barh(range(len(features)), importance_values, color=colors)

    plt.yticks(range(len(features)), features, fontsize=10)
    plt.xlabel('Normalized Importance Score', fontsize=12)
    plt.title('(A)',
              size=14, fontweight='bold', pad=15)
    plt.xlim(0, max(importance_values) * 1.15)

    # Add value labels
    for i, bar in enumerate(bars):
        width = bar.get_width()
        plt.text(width + 0.005, bar.get_y() + bar.get_height()/2,
                 f'{width:.4f}', ha='left', va='center', fontsize=10)

    plt.tight_layout()
    output_path = os.path.join(feature_importance_dir, '608_Overall_Feature_Importance.tiff')
    plt.savefig(output_path, dpi=600, bbox_inches='tight')
    print(f"    ☑ Saved as: {output_path}")
    plt.show()

except Exception as e:
```

```python
    print(f"Error generating feature importance visualization: {e}")

# =================== Print Summary Results ===================
print("\n" + "=" * 80)
print("Performance Comparison Summary")
print("=" * 80)

print(results_df.round(3))

# Find the best model for each metric
print("\nBest model for each metric:")
for metric in ['Accuracy', 'Precision', 'Recall', 'F1 Score', 'AUC-ROC', 'Balanced Accuracy',
'Matthews Correlation Coefficient', 'Cross-validation Accuracy']:
    if metric in results_df.columns:
        best_model = results_df[metric].idxmax()
        best_score = results_df[metric].max()
        print(f"{metric}: {best_model} ({best_score:.3f})")

print("\n" + "=" * 80)
print("Feature Importance Summary")
print("=" * 80)

print("\nOverall feature importance ranking:")
for i, (feature, score) in enumerate(overall_feature_importance_sorted.items(), 1):
    print(f"{i}. {feature}: {score:.4f}")

print("\nTop 3 features for each model:")
for idx, row in feature_importance_df.iterrows():
    print(f"\n{row['Model']}:")
    for feat, val in zip(row['Top_3_Features'], row['Top_3_Values']):
        print(f"    - {feat}: {val:.4f}")

# Find the top 3 most important features
top_3_overall = list(overall_feature_importance_sorted.items())[:3]
print(f"\n    Top 3 most important features:")
for i, (feature, score) in enumerate(top_3_overall, 1):
    print(f"{i}. {feature} (importance score: {score:.4f})")

# Create detailed comparison table
detailed_comparison = results_df.round(4)
detailed_comparison['Overall_Rank'] =
detailed_comparison.mean(axis=1).rank(ascending=False).astype(int)
detailed_comparison = detailed_comparison.sort_values('Overall_Rank')
```

```python
detailed_csv_path = os.path.join(output_dir, '608_Detailed_Performance_Comparison.csv')
detailed_comparison.to_csv(detailed_csv_path, encoding='utf-8-sig')
print(f"\n✅ Detailed performance comparison table saved as: {detailed_csv_path}")

best_overall = detailed_comparison.index[0]
print(f"\n    Recommended best overall model: {best_overall}")
print(f"Overall ranking score: {detailed_comparison.loc[best_overall].mean():.4f}")

# ==================== DeLong Test Final Summary ====================
print("\n" + "=" * 80)
print("DeLong's Test Final Summary")
print("=" * 80)

print(f"\nTotal models compared: {len(model_names_with_probs)}")
print(f"Total pairwise comparisons: {comparison_count}")
print(f"\nSignificance levels:")
print(f"  p < 0.05: {significant_count_05} comparisons ({significant_count_05/comparison_count*100:.1f}%)")
print(f"  p < 0.01: {significant_count_01} comparisons ({significant_count_01/comparison_count*100:.1f}%)")
print(f"  p < 0.001: {significant_count_001} comparisons ({significant_count_001/comparison_count*100:.1f}%)")

print(f"\nBest model by AUC: {best_auc_model} (AUC = {best_auc:.4f})")
print(f"Models not significantly different from {best_auc_model} (p ≥ 0.05):")
for idx, row in best_comparisons.iterrows():
    other_model = row['Model 2'] if row['Model 1'] == best_auc_model else row['Model 1']
    if row['P-Value'] >= 0.05:
        print(f"    - {other_model} (p = {row['P-Value']:.4f})")

# ==================== Final Summary ====================
print("\n" + "=" * 80)
print("Analysis completed successfully!")
print("=" * 80)

print(f"\n    All files generated in '{output_dir}' directory:")

print("\n1. Algorithm model comparison combined plot:")
print(f"    - 608_Algorithm_Comparison_Combined.tiff - Algorithm model comparison combined plot (for publication)")

print("\n2. Performance results files:")
print(f"    - 608_Performance_Results.csv - Performance metrics")
print(f"    - 608_Detailed_Performance_Comparison.csv - Detailed performance comparison")
```

```
print(f"\n3. Feature importance analysis files (in 'feature_importance' subdirectory):")
print(f"      - 608_Feature_Importance_Summary.csv - Feature importance for each model")
print(f"      - 608_Overall_Feature_Importance.csv - Overall feature importance")
print(f"      - 608_Overall_Feature_Importance.tiff - Overall feature importance plot")

print(f"\n4. DeLong's Test files (in 'delong_test' subdirectory):")
print(f"      - 608_Delong_Test_Results.csv - DeLong's test pairwise comparison results")
print(f"      - 608_Delong_Test_Heatmap.tiff - DeLong's test P-value heatmap")

print("\n" + "=" * 80)
print("Key Findings:")
print("=" * 80)
print(f"1. Best performing model: {best_overall}")
print(f"2. Most important features: {', '.join([f[0] for f in top_3_overall])}")
print(f"3. Total number of models evaluated: {len(fixed_optimized_models) + 1} (including ensemble model)")
print(f"4. All feature importance values have been properly normalized, ranging from 0-1")
print(f"5. All charts have been saved in TIFF format, suitable for publication")
print(f"6. DeLong's test completed with {comparison_count} pairwise comparisons")
print(f"7. {significant_count_05} significant differences found (p<0.05) among model AUCs")
print("=" * 80)
```

Feature importance ranking code for the low-age group + DeLong's test

```
# -*- coding: utf-8 -*-
"""
Multiple Machine Learning Algorithms Comparison with Feature Importance Analysis - ASD Severity Prediction (461 Cases)
Using Fixed Optimized Parameters from GridSearchCV Results
Added DeLong's Test for AUC Comparison
"""
import pandas as pd
import numpy as np
from sklearn.model_selection import train_test_split, cross_val_score, StratifiedKFold
from sklearn.preprocessing import StandardScaler
from sklearn.metrics import (accuracy_score, precision_score, recall_score,
                             f1_score, roc_auc_score, matthews_corrcoef,
                             balanced_accuracy_score, roc_curve)
import matplotlib.pyplot as plt
import seaborn as sns
from sklearn.ensemble import RandomForestClassifier, GradientBoostingClassifier, VotingClassifier
from sklearn.svm import SVC
from sklearn.linear_model import LogisticRegression
from sklearn.neighbors import KNeighborsClassifier
from sklearn.tree import DecisionTreeClassifier
from sklearn.inspection import permutation_importance
from scipy import stats
from scipy.stats import norm
import warnings
import os
import json
from datetime import datetime

warnings.filterwarnings('ignore')

# Create output directory - Modified to your specified directory
output_dir = "C:/Correlation between different first diagnosis ages and developmental levels"
if not os.path.exists(output_dir):
    os.makedirs(output_dir)

# Create feature importance subdirectory
feature_importance_dir = os.path.join(output_dir, "feature_importance")
if not os.path.exists(feature_importance_dir):
    os.makedirs(feature_importance_dir)

# Create DeLong test subdirectory
```

```
delong_dir = os.path.join(output_dir, "delong_test")
if not os.path.exists(delong_dir):
    os.makedirs(delong_dir)

# Set English font for plots
plt.rcParams['font.sans-serif'] = ['Arial', 'DejaVu Sans', 'sans-serif']
plt.rcParams['axes.unicode_minus'] = False

# Read data - Changed 'personal social' to 'personal-social'
data = pd.read_excel('C:/Correlation between different first diagnosis ages and developmental levels/461cases Python initial analysis (developmental level grading) version.xlsx',
    usecols=['HAZ', 'WAZ','BAZ','adaptive behavior', 'gross motor', 'fine motor', 'language', 'personal-social', 'Is_Severe'])

# Get feature names - Changed 'personal social' to 'personal-social'
feature_names = ['HAZ', 'WAZ', 'BAZ', 'adaptive behavior', 'gross motor', 'fine motor', 'language', 'personal-social']

print("Data Information:")
print(f"Data Shape: {data.shape}")
print("Features used:", feature_names)
print("Class Distribution:")
print(data['Is_Severe'].value_counts())

# Split data
x_train, x_test, y_train, y_test = train_test_split(
    data.iloc[:,0:8], data.iloc[:,8], test_size=0.2, random_state=30)

# Standardization
scaler = StandardScaler()
x_train_scaled = scaler.fit_transform(x_train)
x_test_scaled = scaler.transform(x_test)

# ==================== Data Augmentation Strategy ====================
print("\nApplying data augmentation strategy...")
try:
    from imblearn.over_sampling import SMOTE
    # Automatically adjust k_neighbors to avoid errors with small samples
    min_samples = y_train.value_counts().min()
    k_neighbors = min(5, min_samples - 1) if min_samples > 1 else 1

    smote = SMOTE(random_state=42, k_neighbors=k_neighbors)
    x_train_scaled, y_train = smote.fit_resample(x_train_scaled, y_train)
    print(f" ☑ SMOTE data augmentation successful! Training samples increased from
```

```
{len(x_train)} to {len(x_train_scaled)}")
except ImportError:
    print("    imblearn library not detected, switching to random oversampling...")
    # Manual simple oversampling implementation
    import numpy as np
    y_train_np = y_train.values if hasattr(y_train, 'values') else y_train
    classes, counts = np.unique(y_train_np, return_counts=True)
    max_count = counts.max()

    x_resampled = []
    y_resampled = []

    for cls in classes:
        cls_idx = np.where(y_train_np == cls)[0]
        x_cls = x_train_scaled[cls_idx]

        if len(x_cls) < max_count:
            indices = np.random.choice(len(x_cls), max_count, replace=True)
            x_cls = x_cls[indices]

        x_resampled.append(x_cls)
        y_resampled.append(np.full(max_count, cls))

    x_train_scaled = np.vstack(x_resampled)
    y_train = np.concatenate(y_resampled)
    print(f"✅ Random oversampling successful! Training samples increased to {len(y_train)}")
except Exception as e:
    print(f"❌ Data augmentation failed: {e}, continuing with original data...")

# Cross-validation strategy
cv_strategy = StratifiedKFold(n_splits=5, shuffle=True, random_state=42)

# =================== Define Models with Fixed Optimized Parameters
===================
print("\n" + "=" * 80)
print("Initializing models with fixed optimized parameters from GridSearchCV...")
print("=" * 80)

# Fixed optimized parameters from your GridSearchCV results
fixed_optimized_models = {
    'Random Forest': RandomForestClassifier(
        class_weight=None,
        max_depth=10,
        max_features='sqrt',
```

```
            min_samples_leaf=1,
            min_samples_split=5,
            n_estimators=80,
            random_state=30,
            n_jobs=-1
        ),
        'Gradient Boosting': GradientBoostingClassifier(
            learning_rate=0.1,
            max_depth=4,
            n_estimators=50,
            subsample=0.8,
            random_state=30
        ),
        'Support Vector Machine': SVC(
            C=0.1,
            class_weight=None,
            gamma=0.1,
            kernel='rbf',
            probability=True,
            random_state=42
        ),
        'Logistic Regression': LogisticRegression(
            C=0.1,
            class_weight=None,
            penalty='l2',
            solver='liblinear',
            max_iter=1000,
            random_state=42
        ),
        'K-Nearest Neighbors': KNeighborsClassifier(
            n_neighbors=11,
            p=2,
            weights='distance'
        ),
}

# =================== DeLong's Test Function ===================
def delong_roc_variance(ground_truth, predictions):
        """
        Calculate the variance of the AUC using DeLong's method
        """
        # Get positive and negative samples
        pos_indices = np.where(ground_truth == 2)[0]    # Assuming 2 is positive class
        neg_indices = np.where(ground_truth == 1)[0]    # Assuming 1 is negative class
```

```
    n_pos = len(pos_indices)
    n_neg = len(neg_indices)

    if n_pos == 0 or n_neg == 0:
        return 0, 0

    # Create the structural components
    v10 = np.zeros((len(ground_truth),))
    v01 = np.zeros((len(ground_truth),))

    for i in range(n_pos):
        for j in range(n_neg):
            if predictions[pos_indices[i]] > predictions[neg_indices[j]]:
                v10[pos_indices[i]] += 1
            elif predictions[pos_indices[i]] < predictions[neg_indices[j]]:
                v01[pos_indices[i]] += 1

    v10 = v10 / n_neg
    v01 = v01 / n_pos

    # Calculate variance
    var_pos = np.var(v10[pos_indices]) / n_pos
    var_neg = np.var(v01[neg_indices]) / n_neg

    return var_pos + var_neg, var_pos, var_neg


def delong_roc_test(ground_truth, pred1, pred2):
    """
    DeLong's test for comparing two AUCs
    """
    # Calculate AUC for both predictions
    auc1 = roc_auc_score(ground_truth, pred1)
    auc2 = roc_auc_score(ground_truth, pred2)

    # Calculate variances
    var1, v1_pos, v1_neg = delong_roc_variance(ground_truth, pred1)
    var2, v2_pos, v2_neg = delong_roc_variance(ground_truth, pred2)

    # Calculate covariance
    pos_indices = np.where(ground_truth == 2)[0]
    neg_indices = np.where(ground_truth == 1)[0]
```

```
    n_pos = len(pos_indices)
    n_neg = len(neg_indices)

    if n_pos == 0 or n_neg == 0:
        return auc1, auc2, 0, 1.0  # Return p-value=1.0 if can't calculate

    # Create structural components for both models
    v10_1 = np.zeros((len(ground_truth),))
    v10_2 = np.zeros((len(ground_truth),))
    v01_1 = np.zeros((len(ground_truth),))
    v01_2 = np.zeros((len(ground_truth),))

    for i in range(n_pos):
        for j in range(n_neg):
            # Model 1
            if pred1[pos_indices[i]] > pred1[neg_indices[j]]:
                v10_1[pos_indices[i]] += 1
            elif pred1[pos_indices[i]] < pred1[neg_indices[j]]:
                v01_1[pos_indices[i]] += 1

            # Model 2
            if pred2[pos_indices[i]] > pred2[neg_indices[j]]:
                v10_2[pos_indices[i]] += 1
            elif pred2[pos_indices[i]] < pred2[neg_indices[j]]:
                v01_2[pos_indices[i]] += 1

    v10_1 = v10_1 / n_neg
    v10_2 = v10_2 / n_neg
    v01_1 = v01_1 / n_pos
    v01_2 = v01_2 / n_pos

    # Calculate covariance
    cov_pos = np.mean(v10_1[pos_indices] * v10_2[pos_indices]) - np.mean(v10_1[pos_indices]) * np.mean(v10_2[pos_indices])
    cov_neg = np.mean(v01_1[neg_indices] * v01_2[neg_indices]) - np.mean(v01_1[neg_indices]) * np.mean(v01_2[neg_indices])

    cov = cov_pos / n_pos + cov_neg / n_neg

    # Calculate z-score
    if var1 + var2 - 2 * cov <= 0:
        z_score = 0
        p_value = 1.0
    else:
```

```
        z_score = (auc1 - auc2) / np.sqrt(var1 + var2 - 2 * cov)
        p_value = 2 * (1 - norm.cdf(np.abs(z_score)))

    return auc1, auc2, z_score, p_value


# Store model performance metrics
results = {
    'Model': [],
    'Accuracy': [], 'Precision': [], 'Recall': [], 'F1 Score': [],
    'AUC-ROC': [], 'Balanced Accuracy': [], 'Matthews Correlation Coefficient': [],
'Cross-validation Accuracy': []
}

# Store model predictions for DeLong test
model_probabilities = {}

# Store feature importance results
feature_importance_results = {
    'Model': [],
    'Features': [],
    'Importance_Values': [],
    'Importance_Rank': [],
    'Top_3_Features': [],
    'Top_3_Values': []
}

# Store best parameters for each model (from your fixed results)
best_params_records = {
    'Model': [],
    'Best Parameters': [],
    'Best CV Score': [],
    'Search_Space_Size': []
}

# Fixed best CV scores and search space sizes from your results
fixed_best_cv_scores = {
    'Random Forest': 0.6568,
    'Gradient Boosting': 0.6617,
    'Support Vector Machine': 0.6401,
    'Logistic Regression': 0.6449,
    'K-Nearest Neighbors': 0.6569,
}
```

```
# Search space sizes from your results
search_space_sizes = {
    'Random Forest': 360,
    'Gradient Boosting': 54,
    'Support Vector Machine': 64,
    'Logistic Regression': 16,
    'KNN': 20,
}

# Store best parameters
for model_name in fixed_optimized_models.keys():
    best_params_records['Model'].append(model_name)
    best_params_records['Best CV Score'].append(fixed_best_cv_scores.get(model_name, 0))
    best_params_records['Search_Space_Size'].append(search_space_sizes.get(model_name,
0))

    if model_name == 'Random Forest':
        best_params_records['Best Parameters'].append({
            'class_weight': None,
            'max_depth': 10,
            'max_features': 'sqrt',
            'min_samples_leaf': 1,
            'min_samples_split': 5,
            'n_estimators': 80
        })
    elif model_name == 'Gradient Boosting':
        best_params_records['Best Parameters'].append({
            'learning_rate': 0.1,
            'max_depth': 4,
            'n_estimators': 50,
            'subsample': 0.8
        })
    elif model_name == 'Support Vector Machine':
        best_params_records['Best Parameters'].append({
            'C': 0.1,
            'class_weight': None,
            'gamma': 0.1,
            'kernel': 'rbf'
        })
    elif model_name == 'Logistic Regression':
        best_params_records['Best Parameters'].append({
            'C': 0.1,
            'class_weight': None,
            'penalty': 'l2',
```

```
            'solver': 'liblinear'
        })
    elif model_name == 'K-Nearest Neighbors':
        best_params_records['Best Parameters'].append({
            'n_neighbors': 11,
            'p': 2,
            'weights': 'distance'
        })

print("\n" + "=" * 80)
print("Starting training and evaluation of each model with fixed optimized parameters...")
print("=" * 80)

# Train and evaluate each model
for model_name, model in fixed_optimized_models.items():
    print(f"\nTraining {model_name} with fixed optimized parameters...")

    # Train model
    model.fit(x_train_scaled, y_train)

    # Cross-validation scores (recalculated)
    cv_scores = cross_val_score(model, x_train_scaled, y_train, cv=cv_strategy,
scoring='accuracy')
    cv_mean = cv_scores.mean()

    # Predictions
    y_pred = model.predict(x_test_scaled)
    y_prob = model.predict_proba(x_test_scaled) if hasattr(model, "predict_proba") else None

    # Store probabilities for DeLong test
    if y_prob is not None:
        model_probabilities[model_name] = y_prob[:, 1]    # Store probability of positive class

    # Calculate metrics
    accuracy = accuracy_score(y_test, y_pred)
    precision = precision_score(y_test, y_pred, average='binary', pos_label=2)
    recall = recall_score(y_test, y_pred, average='binary', pos_label=2)
    f1 = f1_score(y_test, y_pred, average='binary', pos_label=2)

    if y_prob is not None:
        auc_roc = roc_auc_score(y_test, y_prob[:, 1])
    else:
        auc_roc = 0.0
```

```
        balanced_acc = balanced_accuracy_score(y_test, y_pred)
        mcc = matthews_corrcoef(y_test, y_pred)

        # Store results
        results['Model'].append(model_name)
        results['Accuracy'].append(accuracy)
        results['Precision'].append(precision)
        results['Recall'].append(recall)
        results['F1 Score'].append(f1)
        results['AUC-ROC'].append(auc_roc)
        results['Balanced Accuracy'].append(balanced_acc)
        results['Matthews Correlation Coefficient'].append(mcc)
        results['Cross-validation Accuracy'].append(cv_mean)

        print(f"{model_name}  results:  Accuracy={accuracy:.3f}  |  F1={f1:.3f}  |  AUC={auc_roc:.3f}  |
    Cross-validation={cv_mean:.3f}")

        # =================== Feature Importance Analysis ===================
        print(f"    Calculating feature importance for {model_name}...")

        try:
            if model_name in ['Random Forest', 'Gradient Boosting', 'Decision Tree']:
                # Tree-based models have built-in feature importance
                if hasattr(model, 'feature_importances_'):
                    importance = model.feature_importances_
                    importance_type = "Gini Importance"
                else:
                    importance = np.zeros(len(feature_names))
                    importance_type = "Unavailable"

            elif model_name == 'Logistic Regression':
                # Logistic Regression uses coefficients
                if hasattr(model, 'coef_'):
                    importance = np.abs(model.coef_[0])
                    importance_type = "Absolute Coefficient"
                else:
                    importance = np.zeros(len(feature_names))
                    importance_type = "Unavailable"

            elif model_name == 'Support Vector Machine':
                # For non-linear SVM, use permutation importance
                try:
                    perm_importance = permutation_importance(
                        model,    x_test_scaled,    y_test,    n_repeats=10,    random_state=42,
```

```
n_jobs=-1
                )
                importance = np.abs(perm_importance.importances_mean)
                importance_type = "Permutation Importance"
            except:
                importance = np.zeros(len(feature_names))
                importance_type = "Unavailable"

        elif model_name == 'K-Nearest Neighbors':
            # KNN has no built-in importance, use permutation importance
            try:
                perm_importance = permutation_importance(
                    model,    x_test_scaled,    y_test,    n_repeats=10,    random_state=42,
n_jobs=-1
                )
                importance = np.abs(perm_importance.importances_mean)
                importance_type = "Permutation Importance"
            except:
                importance = np.zeros(len(feature_names))
                importance_type = "Unavailable"
        else:
            importance = np.zeros(len(feature_names))
            importance_type = "Unavailable"

        # Normalize importance to sum to 1
        if np.sum(importance) > 0:
            importance_normalized = importance / np.sum(importance)
        else:
            importance_normalized = importance

        # Create ranking
        ranked_indices = np.argsort(importance_normalized)[::-1]
        ranked_features = [feature_names[i] for i in ranked_indices]
        ranked_values = importance_normalized[ranked_indices]

        # Get top 3 features
        top_3_features = ranked_features[:3] if len(ranked_features) >= 3 else ranked_features
        top_3_values = ranked_values[:3] if len(ranked_values) >= 3 else ranked_values

        # Store feature importance results
        feature_importance_results['Model'].append(model_name)
        feature_importance_results['Features'].append(ranked_features)
        feature_importance_results['Importance_Values'].append(ranked_values)
        feature_importance_results['Importance_Rank'].append(list(range(1,
```

```
len(ranked_features) + 1)))
            feature_importance_results['Top_3_Features'].append(top_3_features)
            feature_importance_results['Top_3_Values'].append(top_3_values)

            print(f"    Importance type: {importance_type}")
            print(f"    Top 3 features: {', '.join([f'{feat}({val:.4f})' for feat, val in zip(top_3_features,
top_3_values)])}")

        except Exception as e:
            print(f"    Error calculating feature importance: {e}")
            # Add default values
            feature_importance_results['Model'].append(model_name)
            feature_importance_results['Features'].append(feature_names)

feature_importance_results['Importance_Values'].append(np.zeros(len(feature_names)))
            feature_importance_results['Importance_Rank'].append(list(range(1,
len(feature_names) + 1)))
            feature_importance_results['Top_3_Features'].append([])
            feature_importance_results['Top_3_Values'].append([])

# =================== Add Ensemble Model (Voting Classifier) ===================
print("\n" + "=" * 80)
print("Training ensemble model (soft voting)...")

# 1. Sort models by CV score
model_cv_scores = []
for name, model in fixed_optimized_models.items():
    idx = results['Model'].index(name)
    score = results['Cross-validation Accuracy'][idx]
    model_cv_scores.append((name, model, score))

# Sort in descending order
model_cv_scores.sort(key=lambda x: x[2], reverse=True)

# 2. Select top 4 models
top_n = min(4, len(model_cv_scores))
selected_models = model_cv_scores[:top_n]

print(f"\nTop {len(selected_models)} models for ensemble:")
estimators = []
weights = []
for name, model, score in selected_models:
    print(f"    {name}: CV score = {score:.4f}")
    estimators.append((name, model))
```

```
        weights.append(score)    # Use CV score as weight

# 3. Build weighted VotingClassifier
print(f"Training weighted ensemble model (weighted soft voting)...")
voting_clf = VotingClassifier(estimators=estimators, voting='soft', weights=weights)
voting_clf.fit(x_train_scaled, y_train)

y_pred_vote = voting_clf.predict(x_test_scaled)
y_prob_vote = voting_clf.predict_proba(x_test_scaled)

# Store probabilities for DeLong test - Changed to 'Ensemble Model'
model_probabilities['Ensemble Model'] = y_prob_vote[:, 1]

# Calculate ensemble model metrics
acc_vote = accuracy_score(y_test, y_pred_vote)
prec_vote = precision_score(y_test, y_pred_vote, average='binary', pos_label=2)
rec_vote = recall_score(y_test, y_pred_vote, average='binary', pos_label=2)
f1_vote = f1_score(y_test, y_pred_vote, average='binary', pos_label=2)
auc_vote = roc_auc_score(y_test, y_prob_vote[:, 1])
bal_acc_vote = balanced_accuracy_score(y_test, y_pred_vote)
mcc_vote = matthews_corrcoef(y_test, y_pred_vote)
cv_vote = cross_val_score(voting_clf, x_train_scaled, y_train, cv=cv_strategy,
scoring='accuracy').mean()

# Add ensemble model results - Changed to 'Ensemble Model'
results['Model'].append('Ensemble Model')
results['Accuracy'].append(acc_vote)
results['Precision'].append(prec_vote)
results['Recall'].append(rec_vote)
results['F1 Score'].append(f1_vote)
results['AUC-ROC'].append(auc_vote)
results['Balanced Accuracy'].append(bal_acc_vote)
results['Matthews Correlation Coefficient'].append(mcc_vote)
results['Cross-validation Accuracy'].append(cv_vote)

print(f"Ensemble model results: Accuracy={acc_vote:.3f} | F1={f1_vote:.3f} |
AUC={auc_vote:.3f}")

# =================== DeLong's Test Analysis ===================
print("\n" + "=" * 80)
print("Performing DeLong's Test for AUC Comparison...")
print("=" * 80)

# Create results for DeLong test
```

```
delong_results = {
        'Model 1': [],
        'Model 2': [],
        'AUC 1': [],
        'AUC 2': [],
        'Z-Score': [],
        'P-Value': [],
        'Significant (p<0.05)': [],
        'Significant (p<0.01)': [],
        'Significant (p<0.001)': []
}

# Get all model names that have probability predictions
model_names_with_probs = list(model_probabilities.keys())

print(f"\nTotal {len(model_names_with_probs)} models will be compared:")
for i, name in enumerate(model_names_with_probs):
        auc = roc_auc_score(y_test, model_probabilities[name])
        print(f"    {i+1}. {name}: AUC = {auc:.4f}")

print(f"\nTotal pairwise comparisons: {len(model_names_with_probs) *
(len(model_names_with_probs) - 1) // 2}")

# Perform pairwise comparisons
comparison_count = 0
significant_count_05 = 0
significant_count_01 = 0
significant_count_001 = 0

for i in range(len(model_names_with_probs)):
        for j in range(i + 1, len(model_names_with_probs)):
                model1_name = model_names_with_probs[i]
                model2_name = model_names_with_probs[j]

                prob1 = model_probabilities[model1_name]
                prob2 = model_probabilities[model2_name]

                # Perform DeLong's test
                auc1, auc2, z_score, p_value = delong_roc_test(y_test.values, prob1, prob2)

                # Store results
                delong_results['Model 1'].append(model1_name)
                delong_results['Model 2'].append(model2_name)
                delong_results['AUC 1'].append(auc1)
```

```
            delong_results['AUC 2'].append(auc2)
            delong_results['Z-Score'].append(z_score)
            delong_results['P-Value'].append(p_value)
            delong_results['Significant (p<0.05)'].append('Yes' if p_value < 0.05 else 'No')
            delong_results['Significant (p<0.01)'].append('Yes' if p_value < 0.01 else 'No')
            delong_results['Significant (p<0.001)'].append('Yes' if p_value < 0.001 else 'No')

            comparison_count += 1

            if p_value < 0.05:
                significant_count_05 += 1
                if p_value < 0.01:
                    significant_count_01 += 1
                    if p_value < 0.001:
                        significant_count_001 += 1

            # Print results
            significance_marker = ""
            if p_value < 0.001:
                significance_marker = " ***"
            elif p_value < 0.01:
                significance_marker = " **"
            elif p_value < 0.05:
                significance_marker = " *"

            print(f"    {model1_name} vs {model2_name}:")
            print(f"       AUC: {auc1:.4f} vs {auc2:.4f},   △AUC = {auc1 - auc2:.4f}")
            print(f"       Z-score = {z_score:.4f}, P-value = {p_value:.6f}{significance_marker}")

# Create DeLong test results DataFrame
delong_df = pd.DataFrame(delong_results)

# Save DeLong test results
delong_output_path = os.path.join(delong_dir, '461_Delong_Test_Results.csv')
delong_df.to_csv(delong_output_path, index=False, encoding='utf-8-sig')
print(f"\n✅ DeLong's test results saved as: {delong_output_path}")

# ==================== Create DeLong Test Summary ====================
print("\n" + "=" * 80)
print("DeLong's Test Summary")
print("=" * 80)

print(f"\nTotal pairwise comparisons: {comparison_count}")
print(f"Significant at p<0.05: {significant_count_05}
```

```
({significant_count_05/comparison_count*100:.1f}%)")
print(f"Significant at p<0.01: {significant_count_01}
({significant_count_01/comparison_count*100:.1f}%)")
print(f"Significant at p<0.001: {significant_count_001}
({significant_count_001/comparison_count*100:.1f}%)")

# Find the best model based on AUC
best_auc_model = max(model_names_with_probs, key=lambda x: roc_auc_score(y_test,
model_probabilities[x]))
best_auc = roc_auc_score(y_test, model_probabilities[best_auc_model])

print(f"\nBest performing model based on AUC: {best_auc_model} (AUC = {best_auc:.4f})")

# Check if the best model is significantly better than others
print(f"\nComparisons with {best_auc_model}:")
best_comparisons = delong_df[(delong_df['Model 1'] == best_auc_model) | (delong_df['Model 2']
== best_auc_model)]
for idx, row in best_comparisons.iterrows():
    if row['Model 1'] == best_auc_model:
        other_model = row['Model 2']
    else:
        other_model = row['Model 1']

    significance = ""
    if row['P-Value'] < 0.001:
        significance = "***"
    elif row['P-Value'] < 0.01:
        significance = "**"
    elif row['P-Value'] < 0.05:
        significance = "*"

    print(f"    vs {other_model}: P-value = {row['P-Value']:.6f}{significance}")

# =================== Create DeLong Test Visualization (Heatmap) ===================
print("\n" + "=" * 80)
print("Creating DeLong's Test visualization (P-value Heatmap)...")
print("=" * 80)

try:
    # Create single heatmap of p-values
    fig, ax = plt.subplots(1, 1, figsize=(12, 10))

    # Sort model names
    model_names_sorted = sorted(model_names_with_probs)
```

```
    n_models = len(model_names_sorted)

    p_value_matrix = np.zeros((n_models, n_models))

    for i in range(n_models):
        for j in range(n_models):
            if i == j:
                p_value_matrix[i, j] = 1.0
            else:
                model1 = model_names_sorted[i]
                model2 = model_names_sorted[j]

                # Find the comparison
                for idx, row in delong_df.iterrows():
                    if (row['Model 1'] == model1 and row['Model 2'] == model2) or \
                       (row['Model 1'] == model2 and row['Model 2'] == model1):
                        p_value_matrix[i, j] = row['P-Value']
                        break

    # Create heatmap
    im = ax.imshow(p_value_matrix, cmap='RdYlGn_r', vmin=0, vmax=1)
    ax.set_xticks(range(n_models))
    ax.set_yticks(range(n_models))
    ax.set_xticklabels(model_names_sorted, rotation=45, ha='right', fontsize=10)
    ax.set_yticklabels(model_names_sorted, fontsize=10)
    ax.set_title('(B) DeLong Test P-Value Matrix for AUC Comparison', fontsize=14,
fontweight='bold', pad=15)

    # Add text annotations
    for i in range(n_models):
        for j in range(n_models):
            if i == j:
                text = '1.0000\n(self)'
                text_color = 'black'
            else:
                p_val = p_value_matrix[i, j]
                text = f'{p_val:.4f}'
                if p_val < 0.05:
                    text += '\n*'
                    if p_val < 0.01:
                        text += '*'
                        if p_val < 0.001:
                            text += '*'
```

```
                text_color = 'white' if p_val < 0.3 or p_val > 0.7 else 'black'

            ax.text(j, i, text, ha='center', va='center', fontsize=9, color=text_color,
fontweight='bold')

    # Add colorbar
    cbar = plt.colorbar(im, ax=ax, shrink=0.8)
    cbar.set_label('P-Value', fontsize=11)

    # Add significance legend
    ax.text(0.02, -0.15, '* p < 0.05    ** p < 0.01    *** p < 0.001',
            transform=ax.transAxes, fontsize=9, style='italic')

    plt.tight_layout()
    delong_plot_path = os.path.join(delong_dir, '461_Delong_Test_Heatmap.tiff')
    plt.savefig(delong_plot_path, dpi=600, bbox_inches='tight')
    print(f"✅ DeLong's test P-value heatmap saved as: {delong_plot_path}")
    plt.show()

except Exception as e:
    print(f"Error creating DeLong test visualization: {e}")

# =================== Add feature importance for ensemble model ===================
print("\nCalculating feature importance for ensemble model...")
try:
    ensemble_importance = np.zeros(len(feature_names))
    total_weight = 0

    for (name, _), weight in zip(estimators, weights):
        # Find this model in feature_importance_results
        model_idx = None
        for i, model_name in enumerate(feature_importance_results['Model']):
            if model_name == name:
                model_idx = i
                break

        if model_idx is not None:
            # Get importance values for this model
            importance_values                                                    =
feature_importance_results['Importance_Values'][model_idx]
            # Reorder to original feature order
            features_order = feature_importance_results['Features'][model_idx]
            importance_dict = dict(zip(features_order, importance_values))
```

```
            # Convert to original order
            ordered_importance = np.array([importance_dict.get(feat, 0) for feat in
feature_names])
            ensemble_importance += ordered_importance * weight
            total_weight += weight

    if total_weight > 0:
        ensemble_importance = ensemble_importance / total_weight
        # Normalize
        if np.sum(ensemble_importance) > 0:
            ensemble_importance = ensemble_importance / np.sum(ensemble_importance)

    # Add feature importance results for ensemble model
    ranked_indices = np.argsort(ensemble_importance)[::-1]
    ranked_features = [feature_names[i] for i in ranked_indices]
    ranked_values = ensemble_importance[ranked_indices]
    top_3_features = ranked_features[:3] if len(ranked_features) >= 3 else ranked_features
    top_3_values = ranked_values[:3] if len(ranked_values) >= 3 else ranked_values

    feature_importance_results['Model'].append('Ensemble Model')
    feature_importance_results['Features'].append(ranked_features)
    feature_importance_results['Importance_Values'].append(ranked_values)
    feature_importance_results['Importance_Rank'].append(list(range(1, len(ranked_features) +
1)))
    feature_importance_results['Top_3_Features'].append(top_3_features)
    feature_importance_results['Top_3_Values'].append(top_3_values)

    print(f"Ensemble model top 3 features: {', '.join([f'{feat}({val:.4f})' for feat, val in
zip(top_3_features, top_3_values)])}")

except Exception as e:
    print(f"Error calculating ensemble model feature importance: {e}")

# =================== Create Feature Importance DataFrame ===================
print("\n" + "=" * 80)
print("Creating Feature Importance DataFrame...")
print("=" * 80)

# Create feature importance DataFrame
feature_importance_df = pd.DataFrame(feature_importance_results)
# Save feature importance results
feature_importance_output_path = os.path.join(feature_importance_dir,
'461_Feature_Importance_Summary.csv')
feature_importance_df.to_csv(feature_importance_output_path, index=False,
```

```
encoding='utf-8-sig')
print(f"✅ Feature importance summary saved as: {feature_importance_output_path}")

# =================== Calculate Overall Feature Importance ===================
print("\nCalculating overall feature importance...")

# Create importance matrix for each model
importance_matrix = []
model_names = feature_importance_df['Model'].tolist()

for i, model_name in enumerate(model_names):
    importance_dict = dict(zip(feature_importance_df['Features'].iloc[i],
                               feature_importance_df['Importance_Values'].iloc[i]))

    # Convert to original feature order
    row = [importance_dict.get(feat, 0) for feat in feature_names]
    importance_matrix.append(row)

importance_matrix = np.array(importance_matrix)

# Calculate average importance for each feature (considering only non-zero values)
overall_feature_importance = {}
for j, feat in enumerate(feature_names):
    # Get importance values for this feature across all models
    feat_importances = importance_matrix[:, j]
    # Consider only non-zero values
    non_zero_importances = feat_importances[feat_importances > 0]

    if len(non_zero_importances) > 0:
        overall_feature_importance[feat] = np.mean(non_zero_importances)
    else:
        overall_feature_importance[feat] = 0

# Normalize overall importance to sum to 1
total_importance = sum(overall_feature_importance.values())
if total_importance > 0:
    overall_feature_importance = {k: v/total_importance for k, v in
overall_feature_importance.items()}

# Sort by importance
overall_feature_importance_sorted = dict(sorted(overall_feature_importance.items(),
                                                key=lambda x: x[1], reverse=True))

# Save overall feature importance
```

```
overall_importance_df = pd.DataFrame(list(overall_feature_importance_sorted.items()),
                                     columns=['Feature', 'Overall_Importance'])
overall_importance_output_path = os.path.join(feature_importance_dir,
'461_Overall_Feature_Importance.csv')
overall_importance_df.to_csv(overall_importance_output_path, index=False,
encoding='utf-8-sig')
print(f"☑ Overall feature importance saved as: {overall_importance_output_path}")

# =================== Create Results DataFrame ===================
results_df = pd.DataFrame(results)
results_df.set_index('Model', inplace=True)

# Save performance results
performance_output_path = os.path.join(output_dir, '461_Performance_Results.csv')
results_df.to_csv(performance_output_path, encoding='utf-8-sig')
print(f"☑ Performance results saved as: {performance_output_path}")

# =================== Generate Algorithm Model Comparison Combined Plot
===================
print("\n" + "=" * 80)
print("Generating algorithm model comparison combined plot...")
print("=" * 80)

try:
    # Create combined plot (for publication)
    fig, (ax1, ax2) = plt.subplots(1, 2, figsize=(20, 9))

    # 1. Left subplot - Comprehensive performance comparison
    metrics_for_bar = ['Accuracy', 'Precision', 'Recall', 'F1 Score', 'AUC-ROC', 'Balanced
Accuracy']
    x = np.arange(len(results_df.index))
    width = 0.13
    colors = plt.cm.Set3(np.linspace(0, 1, len(metrics_for_bar)))

    for i, metric in enumerate(metrics_for_bar):
        offset = width * i - (width * (len(metrics_for_bar) - 1) / 2)
        values = results_df[metric].values
        bars = ax1.bar(x + offset, values, width, label=metric, color=colors[i], alpha=0.8)

        for bar, value in zip(bars, values):
            height = bar.get_height()
            # All numbers above bars are uniformly black, placed above the bars
            ax1.text(bar.get_x() + bar.get_width()/2., height + 0.01,
                    f'{value:.3f}', ha='center', va='bottom', fontsize=5, color='black',
```

```
fontweight='bold')

        ax1.set_xlabel('Model', fontsize=11)
        ax1.set_ylabel('Score', fontsize=11)
        ax1.set_title('(C) Performance of different machine models on the testing database', size=14,
fontweight='bold', pad=15)
        ax1.set_xticks(x)
        ax1.set_xticklabels(results_df.index, rotation=15)
        ax1.set_ylim(0, 1.05)
        # Legend uniformly on the right inside the box
        ax1.legend(title='Evaluation Metrics', fontsize=8, title_fontsize=9, loc='upper right')
        ax1.grid(True, alpha=0.3, axis='y')

        # 2. Right subplot - AUC-ROC comparison
        sorted_auc = results_df['AUC-ROC'].sort_values(ascending=True)
        normalized_auc = (sorted_auc.values - sorted_auc.min()) / (sorted_auc.max() -
sorted_auc.min() + 1e-8)
        colors_auc = plt.cm.Blues(normalized_auc * 0.7 + 0.3)

        bars = ax2.barh(range(len(sorted_auc)), sorted_auc.values, color=colors_auc, height=0.6)
        ax2.set_yticks(range(len(sorted_auc)))
        ax2.set_yticklabels(sorted_auc.index)
        ax2.set_xlabel('AUC-ROC Value', fontsize=11)
        ax2.set_title('(D) ', size=14, fontweight='bold', pad=15)
        ax2.set_xlim(0, 1.05)

        for i, bar in enumerate(bars):
                width = bar.get_width()
                label_x = width + 0.01
                if label_x > 1.0:
                        label_x = width - 0.02
                        text_color = 'white'
                else:
                        text_color = 'black'

                ax2.text(label_x, bar.get_y() + bar.get_height()/2,
                                f'{width:.3f}', ha='left', va='center', fontsize=10,
                                fontweight='bold', color=text_color)
        fig.tight_layout()
        # Save as TIFF format
        output_path = os.path.join(output_dir, '461_Algorithm_Comparison_Combined.tiff')
        plt.savefig(output_path, dpi=600, bbox_inches='tight')
        print(f"✅ Algorithm model comparison combined plot saved as: {output_path}")
        plt.show()
```

```
except Exception as e:
    print(f"Error generating combined plot: {e}")

# =================== Generate Feature Importance Visualization ===================
print("\n" + "=" * 80)
print("Generating feature importance visualization...")
print("=" * 80)

try:
    # 1. Overall feature importance plot
    print("\n1. Generating overall feature importance plot...")
    plt.figure(figsize=(12, 8))

    features = list(overall_feature_importance_sorted.keys())
    importance_values = list(overall_feature_importance_sorted.values())

    colors = plt.cm.viridis(np.linspace(0.3, 0.9, len(features)))
    bars = plt.barh(range(len(features)), importance_values, color=colors)

    plt.yticks(range(len(features)), features, fontsize=10)
    plt.xlabel('Normalized Importance Score', fontsize=12)
    plt.title('(B) ',
              size=14, fontweight='bold', pad=15)
    plt.xlim(0, max(importance_values) * 1.15)

    # Add value labels
    for i, bar in enumerate(bars):
        width = bar.get_width()
        plt.text(width + 0.005, bar.get_y() + bar.get_height()/2,
                 f'{width:.4f}', ha='left', va='center', fontsize=10)

    plt.tight_layout()
    output_path = os.path.join(feature_importance_dir, '461_Overall_Feature_Importance.tiff')
    plt.savefig(output_path, dpi=600, bbox_inches='tight')
    print(f"    ✅ Saved as: {output_path}")
    plt.show()

except Exception as e:
    print(f"Error generating feature importance visualization: {e}")

# =================== Print Summary Results ===================
print("\n" + "=" * 80)
print("Performance Comparison Summary")
```

```
print("=" * 80)

print(results_df.round(3))

# Find the best model for each metric
print("\nBest model for each metric:")
for metric in ['Accuracy', 'Precision', 'Recall', 'F1 Score', 'AUC-ROC', 'Balanced Accuracy',
'Matthews Correlation Coefficient', 'Cross-validation Accuracy']:
    if metric in results_df.columns:
        best_model = results_df[metric].idxmax()
        best_score = results_df[metric].max()
        print(f"{metric}: {best_model} ({best_score:.3f})")

print("\n" + "=" * 80)
print("Feature Importance Summary")
print("=" * 80)

print("\nOverall feature importance ranking:")
for i, (feature, score) in enumerate(overall_feature_importance_sorted.items(), 1):
    print(f"{i}. {feature}: {score:.4f}")

print("\nTop 3 features for each model:")
for idx, row in feature_importance_df.iterrows():
    print(f"\n{row['Model']}:")
    for feat, val in zip(row['Top_3_Features'], row['Top_3_Values']):
        print(f"    - {feat}: {val:.4f}")

# Find the top 3 most important features
top_3_overall = list(overall_feature_importance_sorted.items())[:3]
print(f"\n    Top 3 most important features:")
for i, (feature, score) in enumerate(top_3_overall, 1):
    print(f"{i}. {feature} (importance score: {score:.4f})")

# Create detailed comparison table
detailed_comparison = results_df.round(4)
detailed_comparison['Overall_Rank'] =
detailed_comparison.mean(axis=1).rank(ascending=False).astype(int)
detailed_comparison = detailed_comparison.sort_values('Overall_Rank')

detailed_csv_path = os.path.join(output_dir, '461_Detailed_Performance_Comparison.csv')
detailed_comparison.to_csv(detailed_csv_path, encoding='utf-8-sig')
print(f"\n✅ Detailed performance comparison table saved as: {detailed_csv_path}")

best_overall = detailed_comparison.index[0]
```

```
print(f"\n    Recommended best overall model: {best_overall}")
print(f"Overall ranking score: {detailed_comparison.loc[best_overall].mean():.4f}")

# =================== DeLong Test Final Summary ===================
print("\n" + "=" * 80)
print("DeLong's Test Final Summary")
print("=" * 80)

print(f"\nTotal models compared: {len(model_names_with_probs)}")
print(f"Total pairwise comparisons: {comparison_count}")
print(f"\nSignificance levels:")
print(f"    p < 0.05: {significant_count_05} comparisons ({significant_count_05/comparison_count*100:.1f}%)")
print(f"    p < 0.01: {significant_count_01} comparisons ({significant_count_01/comparison_count*100:.1f}%)")
print(f"    p < 0.001: {significant_count_001} comparisons ({significant_count_001/comparison_count*100:.1f}%)")

print(f"\nBest model by AUC: {best_auc_model} (AUC = {best_auc:.4f})")
if best_auc_model != best_overall:
    print(f"Note: Best model by overall performance is {best_overall}")

print(f"\nModels not significantly different from {best_auc_model} (p ⩾ 0.05):")
for idx, row in best_comparisons.iterrows():
    other_model = row['Model 2'] if row['Model 1'] == best_auc_model else row['Model 1']
    if row['P-Value'] >= 0.05:
        print(f"    - {other_model} (p = {row['P-Value']:.4f})")

# =================== Final Summary ===================
print("\n" + "=" * 80)
print("Analysis completed successfully!")
print("=" * 80)

print(f"\n    All files generated in '{output_dir}' directory:")

print("\n1. Algorithm model comparison combined plot:")
print(f"    - 461_Algorithm_Comparison_Combined.tiff - Algorithm model comparison combined plot (for publication)")

print("\n2. Performance results files:")
print(f"    - 461_Performance_Results.csv - Performance metrics")
print(f"    - 461_Detailed_Performance_Comparison.csv - Detailed performance comparison")

print(f"\n3. Feature importance analysis files (in 'feature_importance' subdirectory):")
```

```
print(f"      - 461_Feature_Importance_Summary.csv - Feature importance for each model")
print(f"      - 461_Overall_Feature_Importance.csv - Overall feature importance")
print(f"      - 461_Overall_Feature_Importance.tiff - Overall feature importance plot")

print(f"\n4. DeLong's Test files (in 'delong_test' subdirectory):")
print(f"      - 461_Delong_Test_Results.csv - DeLong's test pairwise comparison results")
print(f"      - 461_Delong_Test_Heatmap.tiff - DeLong's test P-value heatmap")

print("\n" + "=" * 80)
print("Key Findings:")
print("=" * 80)
print(f"1. Best performing model: {best_overall}")
print(f"2. Most important features: {', '.join([f[0] for f in top_3_overall])}")
print(f"3. Total number of models evaluated: {len(fixed_optimized_models) + 1} (including ensemble model)")
print(f"4. All feature importance values have been properly normalized, ranging from 0-1")
print(f"5. All charts have been saved in TIFF format, suitable for publication")
print(f"6. DeLong's test completed with {comparison_count} pairwise comparisons")
print(f"7. {significant_count_05} significant differences found (p<0.05) among model AUCs")
print("=" * 80)
```

Feature importance ranking code for the high-age group + DeLong's test

```
# -*- coding: utf-8 -*-
"""
Multiple Machine Learning Algorithms Comparison (Optimized with GridSearchCV + Ensemble Learning) - ASD Severity Prediction (147 cases)
Using Fixed Optimized Parameters with Feature Importance Analysis
Added DeLong's Test for AUC Comparison
"""
import pandas as pd
import numpy as np
from sklearn.model_selection import train_test_split, cross_val_score, StratifiedKFold
from sklearn.preprocessing import StandardScaler
from sklearn.metrics import (accuracy_score, precision_score, recall_score,
                             f1_score, roc_auc_score, matthews_corrcoef,
                             balanced_accuracy_score, roc_curve)
import matplotlib.pyplot as plt
import seaborn as sns
from sklearn.ensemble import RandomForestClassifier, GradientBoostingClassifier, VotingClassifier
from sklearn.svm import SVC
from sklearn.linear_model import LogisticRegression
from sklearn.neighbors import KNeighborsClassifier
from sklearn.tree import DecisionTreeClassifier
from sklearn.inspection import permutation_importance
from scipy import stats
from scipy.stats import norm
import warnings
import os
import json
from datetime import datetime

warnings.filterwarnings('ignore')

# Create output directory
output_dir = "C:/Correlation between different first diagnosis ages and developmental levels"
if not os.path.exists(output_dir):
    os.makedirs(output_dir)

# Create feature importance subdirectory
feature_importance_dir = os.path.join(output_dir, "feature_importance")
if not os.path.exists(feature_importance_dir):
    os.makedirs(feature_importance_dir)

# Create DeLong test subdirectory
```

```
delong_dir = os.path.join(output_dir, "delong_test")
if not os.path.exists(delong_dir):
    os.makedirs(delong_dir)

# Set English font for plots
plt.rcParams['font.sans-serif'] = ['Arial', 'DejaVu Sans', 'sans-serif']
plt.rcParams['axes.unicode_minus'] = False

# Read data - Changed 'personal social' to 'personal-social'
data = pd.read_excel("C:/Correlation between different first diagnosis ages and developmental
levels/147cases Python initial analysis (developmental level grading) version.xlsx",
    usecols=['HAZ', 'WAZ','BAZ','adaptive behavior', 'gross motor', 'fine motor', 'language',
'personal-social', 'Is_Severe'])

# Get feature names - Changed 'personal social' to 'personal-social'
feature_names = ['HAZ', 'WAZ', 'BAZ', 'adaptive behavior', 'gross motor', 'fine motor', 'language',
'personal-social']
print("Basic data information:")
print(f"Data shape: {data.shape}")
print("Features used:", feature_names)
print("Class distribution:")
print(data['Is_Severe'].value_counts())

# Split data
x_train, x_test, y_train, y_test = train_test_split(
    data.iloc[:,0:8], data.iloc[:,8], test_size=0.3, random_state=42)

# Standardization
scaler = StandardScaler()
x_train_scaled = scaler.fit_transform(x_train)
x_test_scaled = scaler.transform(x_test)

# =================== Data Augmentation Strategy ===================
print("\nApplying data augmentation strategy...")
try:
    from imblearn.over_sampling import SMOTE
    # Automatically adjust k_neighbors to prevent errors with small samples
    min_samples = y_train.value_counts().min()
    k_neighbors = min(5, min_samples - 1) if min_samples > 1 else 1

    smote = SMOTE(random_state=42, k_neighbors=k_neighbors)
    x_train_scaled, y_train = smote.fit_resample(x_train_scaled, y_train)
    print(f"✅ SMOTE data augmentation successful! Training samples increased from
{len(x_train)} to {len(x_train_scaled)}")
```

```
except ImportError:
    print("    imblearn library not detected, switching to random oversampling...")
    import numpy as np
    y_train_np = y_train.values if hasattr(y_train, 'values') else y_train
    classes, counts = np.unique(y_train_np, return_counts=True)
    max_count = counts.max()

    x_resampled = []
    y_resampled = []

    for cls in classes:
        cls_idx = np.where(y_train_np == cls)[0]
        x_cls = x_train_scaled[cls_idx]

        if len(x_cls) < max_count:
            indices = np.random.choice(len(x_cls), max_count, replace=True)
            x_cls = x_cls[indices]

        x_resampled.append(x_cls)
        y_resampled.append(np.full(max_count, cls))

    x_train_scaled = np.vstack(x_resampled)
    y_train = np.concatenate(y_resampled)
    print(f"✅ Random oversampling successful! Training samples increased to {len(y_train)}")
except Exception as e:
    print(f"❌ Data augmentation failed: {e}, continuing with original data...")

# Cross-validation strategy
cv_strategy = StratifiedKFold(n_splits=5, shuffle=True, random_state=42)

# ==================== Define Models with Fixed Optimized Parameters
====================
print("\n" + "=" * 80)
print("Initializing models with fixed optimized parameters from GridSearchCV...")
print("=" * 80)

# Fixed optimized parameters from your GridSearchCV results
fixed_optimized_models = {
    'Random Forest': RandomForestClassifier(
        class_weight=None,
        max_depth=5,
        max_features='sqrt',
        min_samples_leaf=4,
        min_samples_split=5,
```

```
            n_estimators=200,
            random_state=42,
            n_jobs=-1
        ),
        'Gradient Boosting': GradientBoostingClassifier(
            learning_rate=0.05,
            max_depth=5,
            n_estimators=100,
            subsample=0.8,
            random_state=42
        ),
        'Support Vector Machine': SVC(
            C=100,
            class_weight=None,
            gamma=0.1,
            kernel='rbf',
            probability=True,
            random_state=42
        ),
        'Logistic Regression': LogisticRegression(
            C=1,
            class_weight='balanced',
            penalty='l2',
            solver='lbfgs',
            max_iter=1000,
            random_state=42
        ),
        'K-Nearest Neighbors': KNeighborsClassifier(
            n_neighbors=5,
            p=2,
            weights='distance'
        ),
    }

# =================== DeLong's Test Function ===================
def delong_roc_variance(ground_truth, predictions):
    """
    Calculate the variance of the AUC using DeLong's method
    """
    # Get positive and negative samples
    pos_indices = np.where(ground_truth == 2)[0]    # Assuming 2 is positive class
    neg_indices = np.where(ground_truth == 1)[0]    # Assuming 1 is negative class

    n_pos = len(pos_indices)
```

```
    n_neg = len(neg_indices)

    if n_pos == 0 or n_neg == 0:
        return 0, 0

    # Create the structural components
    v10 = np.zeros((len(ground_truth),))
    v01 = np.zeros((len(ground_truth),))

    for i in range(n_pos):
        for j in range(n_neg):
            if predictions[pos_indices[i]] > predictions[neg_indices[j]]:
                v10[pos_indices[i]] += 1
            elif predictions[pos_indices[i]] < predictions[neg_indices[j]]:
                v01[pos_indices[i]] += 1

    v10 = v10 / n_neg
    v01 = v01 / n_pos

    # Calculate variance
    var_pos = np.var(v10[pos_indices]) / n_pos
    var_neg = np.var(v01[neg_indices]) / n_neg

    return var_pos + var_neg, var_pos, var_neg


def delong_roc_test(ground_truth, pred1, pred2):
    """
    DeLong's test for comparing two AUCs
    """
    # Calculate AUC for both predictions
    auc1 = roc_auc_score(ground_truth, pred1)
    auc2 = roc_auc_score(ground_truth, pred2)

    # Calculate variances
    var1, v1_pos, v1_neg = delong_roc_variance(ground_truth, pred1)
    var2, v2_pos, v2_neg = delong_roc_variance(ground_truth, pred2)

    # Calculate covariance
    pos_indices = np.where(ground_truth == 2)[0]
    neg_indices = np.where(ground_truth == 1)[0]

    n_pos = len(pos_indices)
    n_neg = len(neg_indices)
```

```
    if n_pos == 0 or n_neg == 0:
        return auc1, auc2, 0, 1.0    # Return p-value=1.0 if can't calculate

    # Create structural components for both models
    v10_1 = np.zeros((len(ground_truth),))
    v10_2 = np.zeros((len(ground_truth),))
    v01_1 = np.zeros((len(ground_truth),))
    v01_2 = np.zeros((len(ground_truth),))

    for i in range(n_pos):
        for j in range(n_neg):
            # Model 1
            if pred1[pos_indices[i]] > pred1[neg_indices[j]]:
                v10_1[pos_indices[i]] += 1
            elif pred1[pos_indices[i]] < pred1[neg_indices[j]]:
                v01_1[pos_indices[i]] += 1

            # Model 2
            if pred2[pos_indices[i]] > pred2[neg_indices[j]]:
                v10_2[pos_indices[i]] += 1
            elif pred2[pos_indices[i]] < pred2[neg_indices[j]]:
                v01_2[pos_indices[i]] += 1

    v10_1 = v10_1 / n_neg
    v10_2 = v10_2 / n_neg
    v01_1 = v01_1 / n_pos
    v01_2 = v01_2 / n_pos

    # Calculate covariance
    cov_pos = np.mean(v10_1[pos_indices] * v10_2[pos_indices]) - np.mean(v10_1[pos_indices]) * np.mean(v10_2[pos_indices])
    cov_neg = np.mean(v01_1[neg_indices] * v01_2[neg_indices]) - np.mean(v01_1[neg_indices]) * np.mean(v01_2[neg_indices])

    cov = cov_pos / n_pos + cov_neg / n_neg

    # Calculate z-score
    if var1 + var2 - 2 * cov <= 0:
        z_score = 0
        p_value = 1.0
    else:
        z_score = (auc1 - auc2) / np.sqrt(var1 + var2 - 2 * cov)
        p_value = 2 * (1 - norm.cdf(np.abs(z_score)))
```

```
    return auc1, auc2, z_score, p_value


# Store model performance metrics
results = {
    'Model': [],
    'Accuracy': [], 'Precision': [], 'Recall': [], 'F1 Score': [],
    'AUC-ROC': [], 'Balanced Accuracy': [], 'Matthews Correlation Coefficient': [],
'Cross-validation Accuracy': []
}

# Store model predictions for DeLong test
model_probabilities = {}

# Store feature importance results
feature_importance_results = {
    'Model': [],
    'Features': [],
    'Importance_Values': [],
    'Importance_Rank': [],
    'Top_3_Features': [],
    'Top_3_Values': []
}

# Store best parameters for each model (from your fixed results)
best_params_records = {
    'Model': [],
    'Best Parameters': [],
    'Best CV Score': [],
    'Search_Space_Size': []
}

# Fixed best CV scores and search space sizes from your results
fixed_best_cv_scores = {
    'Random Forest': 0.6352,
    'Gradient Boosting': 0.6052,
    'Support Vector Machine': 0.5967,
    'Logistic Regression': 0.7114,
    'K-Nearest Neighbors': 0.7019,
}

# Search space sizes from your results
search_space_sizes = {
```

```
    'Random Forest': 180,
    'Gradient Boosting': 54,
    'Support Vector Machine': 64,
    'Logistic Regression': 16,
    'K-Nearest Neighbors': 20,
}

# Store best parameters
for model_name in fixed_optimized_models.keys():
    best_params_records['Model'].append(model_name)
    best_params_records['Best CV Score'].append(fixed_best_cv_scores.get(model_name, 0))
    best_params_records['Search_Space_Size'].append(search_space_sizes.get(model_name,
0))

    if model_name == 'Random Forest':
        best_params_records['Best Parameters'].append({
            'class_weight': None,
            'max_depth': 5,
            'max_features': 'sqrt',
            'min_samples_leaf': 4,
            'min_samples_split': 5,
            'n_estimators': 200
        })
    elif model_name == 'Gradient Boosting':
        best_params_records['Best Parameters'].append({
            'learning_rate': 0.05,
            'max_depth': 5,
            'n_estimators': 100,
            'subsample': 0.8
        })
    elif model_name == 'Support Vector Machine':
        best_params_records['Best Parameters'].append({
            'C': 100,
            'class_weight': None,
            'gamma': 0.1,
            'kernel': 'rbf'
        })
    elif model_name == 'Logistic Regression':
        best_params_records['Best Parameters'].append({
            'C': 1,
            'class_weight': 'balanced',
            'penalty': 'l2',
            'solver': 'lbfgs'
        })
```

```
    elif model_name == 'K-Nearest Neighbors':
        best_params_records['Best Parameters'].append({
            'n_neighbors': 5,
            'p': 2,
            'weights': 'distance'
        })

print("\n" + "=" * 80)
print("Starting training and evaluation of each model with fixed optimized parameters...")
print("=" * 80)

# Train and evaluate each model
for model_name, model in fixed_optimized_models.items():
    print(f"\nTraining {model_name} with fixed optimized parameters...")

    # Train model
    model.fit(x_train_scaled, y_train)

    # Cross-validation scores (recalculated)
    cv_scores = cross_val_score(model, x_train_scaled, y_train, cv=cv_strategy,
scoring='accuracy')
    cv_mean = cv_scores.mean()

    # Predictions
    y_pred = model.predict(x_test_scaled)
    y_prob = model.predict_proba(x_test_scaled) if hasattr(model, "predict_proba") else None

    # Store probabilities for DeLong test
    if y_prob is not None:
        model_probabilities[model_name] = y_prob[:, 1]    # Store probability of positive class

    # Calculate metrics
    accuracy = accuracy_score(y_test, y_pred)
    precision = precision_score(y_test, y_pred, average='binary', pos_label=2)
    recall = recall_score(y_test, y_pred, average='binary', pos_label=2)
    f1 = f1_score(y_test, y_pred, average='binary', pos_label=2)

    if y_prob is not None:
        auc_roc = roc_auc_score(y_test, y_prob[:, 1])
    else:
        auc_roc = 0.0

    balanced_acc = balanced_accuracy_score(y_test, y_pred)
    mcc = matthews_corrcoef(y_test, y_pred)
```

```
        # Store results
        results['Model'].append(model_name)
        results['Accuracy'].append(accuracy)
        results['Precision'].append(precision)
        results['Recall'].append(recall)
        results['F1 Score'].append(f1)
        results['AUC-ROC'].append(auc_roc)
        results['Balanced Accuracy'].append(balanced_acc)
        results['Matthews Correlation Coefficient'].append(mcc)
        results['Cross-validation Accuracy'].append(cv_mean)

        print(f"{model_name}  results:  Accuracy={accuracy:.3f}  |  F1={f1:.3f}  |  AUC={auc_roc:.3f}  |
Cross-validation={cv_mean:.3f}")

        # =================== Feature Importance Analysis ===================
        print(f"    Calculating feature importance for {model_name}...")

        try:
            if model_name in ['Random Forest', 'Gradient Boosting', 'Decision Tree']:
                # Tree-based models have built-in feature importance
                if hasattr(model, 'feature_importances_'):
                    importance = model.feature_importances_
                    importance_type = "Gini Importance"
                else:
                    importance = np.zeros(len(feature_names))
                    importance_type = "Unavailable"

            elif model_name == 'Logistic Regression':
                # Logistic Regression uses coefficients
                if hasattr(model, 'coef_'):
                    importance = np.abs(model.coef_[0])
                    importance_type = "Absolute Coefficient"
                else:
                    importance = np.zeros(len(feature_names))
                    importance_type = "Unavailable"

            elif model_name == 'Support Vector Machine':
                # For non-linear SVM, use permutation importance
                try:
                    perm_importance = permutation_importance(
                        model,     x_test_scaled,     y_test,     n_repeats=10,     random_state=42,
n_jobs=-1
                    )
```

```
                importance = np.abs(perm_importance.importances_mean)
                importance_type = "Permutation Importance"
            except:
                importance = np.zeros(len(feature_names))
                importance_type = "Unavailable"

        elif model_name == 'K-Nearest Neighbors':
            # KNN has no built-in importance, use permutation importance
            try:
                perm_importance = permutation_importance(
                    model, x_test_scaled, y_test, n_repeats=10, random_state=42,
n_jobs=-1
                )
                importance = np.abs(perm_importance.importances_mean)
                importance_type = "Permutation Importance"
            except:
                importance = np.zeros(len(feature_names))
                importance_type = "Unavailable"
        else:
            importance = np.zeros(len(feature_names))
            importance_type = "Unavailable"

        # Normalize importance to sum to 1
        if np.sum(importance) > 0:
            importance_normalized = importance / np.sum(importance)
        else:
            importance_normalized = importance

        # Create ranking
        ranked_indices = np.argsort(importance_normalized)[::-1]
        ranked_features = [feature_names[i] for i in ranked_indices]
        ranked_values = importance_normalized[ranked_indices]

        # Get top 3 features
        top_3_features = ranked_features[:3] if len(ranked_features) >= 3 else ranked_features
        top_3_values = ranked_values[:3] if len(ranked_values) >= 3 else ranked_values

        # Store feature importance results
        feature_importance_results['Model'].append(model_name)
        feature_importance_results['Features'].append(ranked_features)
        feature_importance_results['Importance_Values'].append(ranked_values)
        feature_importance_results['Importance_Rank'].append(list(range(1,
len(ranked_features) + 1)))
        feature_importance_results['Top_3_Features'].append(top_3_features)
```

```
            feature_importance_results['Top_3_Values'].append(top_3_values)

            print(f"    Importance type: {importance_type}")
            print(f"    Top 3 features: {', '.join([f'{feat}({val:.4f})' for feat, val in zip(top_3_features,
top_3_values)])}")

        except Exception as e:
            print(f"    Error calculating feature importance: {e}")
            # Add default values
            feature_importance_results['Model'].append(model_name)
            feature_importance_results['Features'].append(feature_names)

feature_importance_results['Importance_Values'].append(np.zeros(len(feature_names)))
            feature_importance_results['Importance_Rank'].append(list(range(1,
len(feature_names) + 1)))
            feature_importance_results['Top_3_Features'].append([])
            feature_importance_results['Top_3_Values'].append([])

# =================== Add Ensemble Model (Voting Classifier) ===================
print("\n" + "=" * 80)
print("Training ensemble model (soft voting)...")

# 1. Sort models by CV score
model_cv_scores = []
for name, model in fixed_optimized_models.items():
    idx = results['Model'].index(name)
    score = results['Cross-validation Accuracy'][idx]
    model_cv_scores.append((name, model, score))

# Sort in descending order
model_cv_scores.sort(key=lambda x: x[2], reverse=True)

# 2. Select top 4 models
top_n = min(4, len(model_cv_scores))
selected_models = model_cv_scores[:top_n]

print(f"\nTop {len(selected_models)} models for ensemble:")
estimators = []
weights = []
for name, model, score in selected_models:
    print(f"    {name}: CV score = {score:.4f}")
    estimators.append((name, model))
    weights.append(score)    # Use CV score as weight
```

```
# 3. Build weighted VotingClassifier
print(f"Training weighted ensemble model (weighted soft voting)...")
voting_clf = VotingClassifier(estimators=estimators, voting='soft', weights=weights)
voting_clf.fit(x_train_scaled, y_train)

y_pred_vote = voting_clf.predict(x_test_scaled)
y_prob_vote = voting_clf.predict_proba(x_test_scaled)

# Store probabilities for DeLong test - Changed to 'Ensemble Model'
model_probabilities['Ensemble Model'] = y_prob_vote[:, 1]

# Calculate ensemble model metrics
acc_vote = accuracy_score(y_test, y_pred_vote)
prec_vote = precision_score(y_test, y_pred_vote, average='binary', pos_label=2)
rec_vote = recall_score(y_test, y_pred_vote, average='binary', pos_label=2)
f1_vote = f1_score(y_test, y_pred_vote, average='binary', pos_label=2)
auc_vote = roc_auc_score(y_test, y_prob_vote[:, 1])
bal_acc_vote = balanced_accuracy_score(y_test, y_pred_vote)
mcc_vote = matthews_corrcoef(y_test, y_pred_vote)
cv_vote = cross_val_score(voting_clf, x_train_scaled, y_train, cv=cv_strategy,
scoring='accuracy').mean()

# Add ensemble model results - Changed to 'Ensemble Model'
results['Model'].append('Ensemble Model')
results['Accuracy'].append(acc_vote)
results['Precision'].append(prec_vote)
results['Recall'].append(rec_vote)
results['F1 Score'].append(f1_vote)
results['AUC-ROC'].append(auc_vote)
results['Balanced Accuracy'].append(bal_acc_vote)
results['Matthews Correlation Coefficient'].append(mcc_vote)
results['Cross-validation Accuracy'].append(cv_vote)

print(f"Ensemble model results: Accuracy={acc_vote:.3f} | F1={f1_vote:.3f} |
AUC={auc_vote:.3f}")

# =================== DeLong's Test Analysis ====================
print("\n" + "=" * 80)
print("Performing DeLong's Test for AUC Comparison...")
print("=" * 80)

# Create results for DeLong test
delong_results = {
    'Model 1': [],
```

```
    'Model 2': [],
    'AUC 1': [],
    'AUC 2': [],
    'Z-Score': [],
    'P-Value': [],
    'Significant (p<0.05)': [],
    'Significant (p<0.01)': [],
    'Significant (p<0.001)': []
}

# Get all model names that have probability predictions
model_names_with_probs = list(model_probabilities.keys())

print(f"\nTotal {len(model_names_with_probs)} models will be compared:")
for i, name in enumerate(model_names_with_probs):
    auc = roc_auc_score(y_test, model_probabilities[name])
    print(f"    {i+1}. {name}: AUC = {auc:.4f}")

print(f"\nTotal pairwise comparisons: {len(model_names_with_probs) * (len(model_names_with_probs) - 1) // 2}")

# Perform pairwise comparisons
comparison_count = 0
significant_count_05 = 0
significant_count_01 = 0
significant_count_001 = 0

for i in range(len(model_names_with_probs)):
    for j in range(i + 1, len(model_names_with_probs)):
        model1_name = model_names_with_probs[i]
        model2_name = model_names_with_probs[j]

        prob1 = model_probabilities[model1_name]
        prob2 = model_probabilities[model2_name]

        # Perform DeLong's test
        auc1, auc2, z_score, p_value = delong_roc_test(y_test.values, prob1, prob2)

        # Store results
        delong_results['Model 1'].append(model1_name)
        delong_results['Model 2'].append(model2_name)
        delong_results['AUC 1'].append(auc1)
        delong_results['AUC 2'].append(auc2)
        delong_results['Z-Score'].append(z_score)
```

```
            delong_results['P-Value'].append(p_value)
            delong_results['Significant (p<0.05)'].append('Yes' if p_value < 0.05 else 'No')
            delong_results['Significant (p<0.01)'].append('Yes' if p_value < 0.01 else 'No')
            delong_results['Significant (p<0.001)'].append('Yes' if p_value < 0.001 else 'No')

            comparison_count += 1

            if p_value < 0.05:
                significant_count_05 += 1
                if p_value < 0.01:
                    significant_count_01 += 1
                    if p_value < 0.001:
                        significant_count_001 += 1

            # Print results
            significance_marker = ""
            if p_value < 0.001:
                significance_marker = " ***"
            elif p_value < 0.01:
                significance_marker = " **"
            elif p_value < 0.05:
                significance_marker = " *"

            print(f"    {model1_name} vs {model2_name}:")
            print(f"       AUC: {auc1:.4f} vs {auc2:.4f},   ΔAUC = {auc1 - auc2:.4f}")
            print(f"       Z-score = {z_score:.4f}, P-value = {p_value:.6f}{significance_marker}")

# Create DeLong test results DataFrame
delong_df = pd.DataFrame(delong_results)

# Save DeLong test results
delong_output_path = os.path.join(delong_dir, '147_Delong_Test_Results.csv')
delong_df.to_csv(delong_output_path, index=False, encoding='utf-8-sig')
print(f"\n✅ DeLong's test results saved as: {delong_output_path}")

# =================== Create DeLong Test Summary ===================
print("\n" + "=" * 80)
print("DeLong's Test Summary")
print("=" * 80)

print(f"\nTotal pairwise comparisons: {comparison_count}")
print(f"Significant at p<0.05: {significant_count_05}
({significant_count_05/comparison_count*100:.1f}%)")
print(f"Significant at p<0.01: {significant_count_01}
```

```
({significant_count_01/comparison_count*100:.1f}%)")
print(f"Significant at p<0.001: {significant_count_001}
({significant_count_001/comparison_count*100:.1f}%)")

# Find the best model based on AUC
best_auc_model = max(model_names_with_probs, key=lambda x: roc_auc_score(y_test,
model_probabilities[x]))
best_auc = roc_auc_score(y_test, model_probabilities[best_auc_model])

print(f"\nBest performing model based on AUC: {best_auc_model} (AUC = {best_auc:.4f})")

# Check if the best model is significantly better than others
print(f"\nComparisons with {best_auc_model}:")
best_comparisons = delong_df[(delong_df['Model 1'] == best_auc_model) | (delong_df['Model 2']
== best_auc_model)]
for idx, row in best_comparisons.iterrows():
    if row['Model 1'] == best_auc_model:
        other_model = row['Model 2']
    else:
        other_model = row['Model 1']

    significance = ""
    if row['P-Value'] < 0.001:
        significance = "***"
    elif row['P-Value'] < 0.01:
        significance = "**"
    elif row['P-Value'] < 0.05:
        significance = "*"

    print(f"    vs {other_model}: P-value = {row['P-Value']:.6f}{significance}")

# =================== Create DeLong Test Visualization (Heatmap) ===================
print("\n" + "=" * 80)
print("Creating DeLong's Test visualization (P-value Heatmap)...")
print("=" * 80)

try:
    # Create single heatmap of p-values
    fig, ax = plt.subplots(1, 1, figsize=(12, 10))

    # Sort model names
    model_names_sorted = sorted(model_names_with_probs)
    n_models = len(model_names_sorted)
```

```
p_value_matrix = np.zeros((n_models, n_models))

for i in range(n_models):
    for j in range(n_models):
        if i == j:
            p_value_matrix[i, j] = 1.0
        else:
            model1 = model_names_sorted[i]
            model2 = model_names_sorted[j]

            # Find the comparison
            for idx, row in delong_df.iterrows():
                if (row['Model 1'] == model1 and row['Model 2'] == model2) or \
                   (row['Model 1'] == model2 and row['Model 2'] == model1):
                    p_value_matrix[i, j] = row['P-Value']
                    break

# Create heatmap
im = ax.imshow(p_value_matrix, cmap='RdYlGn_r', vmin=0, vmax=1)
ax.set_xticks(range(n_models))
ax.set_yticks(range(n_models))
ax.set_xticklabels(model_names_sorted, rotation=45, ha='right', fontsize=10)
ax.set_yticklabels(model_names_sorted, fontsize=10)
ax.set_title('(C) DeLong Test P-Value Matrix for AUC Comparison ',
             fontsize=14, fontweight='bold', pad=15)

# Add text annotations
for i in range(n_models):
    for j in range(n_models):
        if i == j:
            text = '1.0000\n(self)'
            text_color = 'black'
        else:
            p_val = p_value_matrix[i, j]
            text = f'{p_val:.4f}'
            if p_val < 0.05:
                text += '\n*'
                if p_val < 0.01:
                    text += '*'
                    if p_val < 0.001:
                        text += '*'

            text_color = 'white' if p_val < 0.3 or p_val > 0.7 else 'black'
```

```
                ax.text(j, i, text, ha='center', va='center', fontsize=9, color=text_color,
fontweight='bold')

        # Add colorbar
        cbar = plt.colorbar(im, ax=ax, shrink=0.8)
        cbar.set_label('P-Value', fontsize=11)

        # Add significance legend
        ax.text(0.02, -0.15, '* p < 0.05    ** p < 0.01    *** p < 0.001',
                transform=ax.transAxes, fontsize=9, style='italic')

        plt.tight_layout()
        delong_plot_path = os.path.join(delong_dir, '147_Delong_Test_Heatmap.tiff')
        plt.savefig(delong_plot_path, dpi=600, bbox_inches='tight')
        print(f"✅ DeLong's test P-value heatmap saved as: {delong_plot_path}")
        plt.show()

except Exception as e:
        print(f"Error creating DeLong test visualization: {e}")

# =================== Add feature importance for ensemble model ===================
print("\nCalculating feature importance for ensemble model...")
try:
        ensemble_importance = np.zeros(len(feature_names))
        total_weight = 0

        for (name, _), weight in zip(estimators, weights):
                # Find this model in feature_importance_results
                model_idx = None
                for i, model_name in enumerate(feature_importance_results['Model']):
                        if model_name == name:
                                model_idx = i
                                break

                if model_idx is not None:
                        # Get importance values for this model
                        importance_values =
feature_importance_results['Importance_Values'][model_idx]
                        # Reorder to original feature order
                        features_order = feature_importance_results['Features'][model_idx]
                        importance_dict = dict(zip(features_order, importance_values))

                        # Convert to original order
                        ordered_importance = np.array([importance_dict.get(feat, 0) for feat in
```

```
feature_names])
                    ensemble_importance += ordered_importance * weight
                    total_weight += weight

        if total_weight > 0:
            ensemble_importance = ensemble_importance / total_weight
            # Normalize
            if np.sum(ensemble_importance) > 0:
                ensemble_importance = ensemble_importance / np.sum(ensemble_importance)

        # Add feature importance results for ensemble model
        ranked_indices = np.argsort(ensemble_importance)[::-1]
        ranked_features = [feature_names[i] for i in ranked_indices]
        ranked_values = ensemble_importance[ranked_indices]
        top_3_features = ranked_features[:3] if len(ranked_features) >= 3 else ranked_features
        top_3_values = ranked_values[:3] if len(ranked_values) >= 3 else ranked_values

        feature_importance_results['Model'].append('Ensemble Model')
        feature_importance_results['Features'].append(ranked_features)
        feature_importance_results['Importance_Values'].append(ranked_values)
        feature_importance_results['Importance_Rank'].append(list(range(1, len(ranked_features) +
1)))
        feature_importance_results['Top_3_Features'].append(top_3_features)
        feature_importance_results['Top_3_Values'].append(top_3_values)

        print(f"Ensemble model top 3 features: {', '.join([f'{feat}({val:.4f})' for feat, val in
zip(top_3_features, top_3_values)])}")

except Exception as e:
    print(f"Error calculating ensemble model feature importance: {e}")

# =================== Create Feature Importance DataFrame ===================
print("\n" + "=" * 80)
print("Creating Feature Importance DataFrame...")
print("=" * 80)

# Create feature importance DataFrame
feature_importance_df = pd.DataFrame(feature_importance_results)
# Save feature importance results
feature_importance_output_path = os.path.join(feature_importance_dir,
'147_Feature_Importance_Summary.csv')
feature_importance_df.to_csv(feature_importance_output_path, index=False,
encoding='utf-8-sig')
print(f"✅ Feature importance summary saved as: {feature_importance_output_path}")
```

```
# =================== Calculate Overall Feature Importance ===================
print("\nCalculating overall feature importance...")

# Create importance matrix for each model
importance_matrix = []
model_names = feature_importance_df['Model'].tolist()

for i, model_name in enumerate(model_names):
    importance_dict = dict(zip(feature_importance_df['Features'].iloc[i],
                               feature_importance_df['Importance_Values'].iloc[i]))

    # Convert to original feature order
    row = [importance_dict.get(feat, 0) for feat in feature_names]
    importance_matrix.append(row)

importance_matrix = np.array(importance_matrix)

# Calculate average importance for each feature (considering only non-zero values)
overall_feature_importance = {}
for j, feat in enumerate(feature_names):
    # Get importance values for this feature across all models
    feat_importances = importance_matrix[:, j]
    # Consider only non-zero values
    non_zero_importances = feat_importances[feat_importances > 0]

    if len(non_zero_importances) > 0:
        overall_feature_importance[feat] = np.mean(non_zero_importances)
    else:
        overall_feature_importance[feat] = 0

# Normalize overall importance to sum to 1
total_importance = sum(overall_feature_importance.values())
if total_importance > 0:
    overall_feature_importance = {k: v/total_importance for k, v in
overall_feature_importance.items()}

# Sort by importance
overall_feature_importance_sorted = dict(sorted(overall_feature_importance.items(),
                                                key=lambda x: x[1], reverse=True))

# Save overall feature importance
overall_importance_df = pd.DataFrame(list(overall_feature_importance_sorted.items()),
                                     columns=['Feature', 'Overall_Importance'])
```

```
overall_importance_output_path = os.path.join(feature_importance_dir,
'147_Overall_Feature_Importance.csv')
overall_importance_df.to_csv(overall_importance_output_path, index=False,
encoding='utf-8-sig')
print(f"☑ Overall feature importance saved as: {overall_importance_output_path}")

# ==================== Create Results DataFrame ====================
results_df = pd.DataFrame(results)
results_df.set_index('Model', inplace=True)

# Save performance results
performance_output_path = os.path.join(output_dir, '147_Performance_Results.csv')
results_df.to_csv(performance_output_path, encoding='utf-8-sig')
print(f"☑ Performance results saved as: {performance_output_path}")

# ==================== Generate Algorithm Model Comparison Combined Plot
====================
print("\n" + "=" * 80)
print("Generating algorithm model comparison combined plot...")
print("=" * 80)

try:
    # Create combined plot (for publication)
    fig, (ax1, ax2) = plt.subplots(1, 2, figsize=(20, 9))

    # 1. Left subplot - Comprehensive performance comparison
    metrics_for_bar = ['Accuracy', 'Precision', 'Recall', 'F1 Score', 'AUC-ROC', 'Balanced
Accuracy']
    x = np.arange(len(results_df.index))
    width = 0.13
    colors = plt.cm.Set3(np.linspace(0, 1, len(metrics_for_bar)))

    for i, metric in enumerate(metrics_for_bar):
        offset = width * i - (width * (len(metrics_for_bar) - 1) / 2)
        values = results_df[metric].values
        bars = ax1.bar(x + offset, values, width, label=metric, color=colors[i], alpha=0.8)

        for bar, value in zip(bars, values):
            height = bar.get_height()
            # All numbers above bars are uniformly black, placed above the bars
            ax1.text(bar.get_x() + bar.get_width()/2., height + 0.01,
                    f'{value:.3f}', ha='center', va='bottom', fontsize=5, color='black',
fontweight='bold')
```

```
    ax1.set_xlabel('Model', fontsize=11)
    ax1.set_ylabel('Score', fontsize=11)
    ax1.set_title('(E) Performance of different machine models on the testing database', size=14, fontweight='bold', pad=15)
    ax1.set_xticks(x)
    ax1.set_xticklabels(results_df.index, rotation=15)
    ax1.set_ylim(0, 1.05)
    # Legend uniformly on the right inside the box
    ax1.legend(title='Evaluation Metrics', fontsize=8, title_fontsize=9, loc='upper right')
    ax1.grid(True, alpha=0.3, axis='y')

    # 2. Right subplot - AUC-ROC comparison
    sorted_auc = results_df['AUC-ROC'].sort_values(ascending=True)
    normalized_auc = (sorted_auc.values - sorted_auc.min()) / (sorted_auc.max() - sorted_auc.min() + 1e-8)
    colors_auc = plt.cm.Blues(normalized_auc * 0.7 + 0.3)

    bars = ax2.barh(range(len(sorted_auc)), sorted_auc.values, color=colors_auc, height=0.6)
    ax2.set_yticks(range(len(sorted_auc)))
    ax2.set_yticklabels(sorted_auc.index)
    ax2.set_xlabel('AUC-ROC Value', fontsize=11)
    ax2.set_title('(F) ', size=14, fontweight='bold', pad=15)
    ax2.set_xlim(0, 1.05)

    for i, bar in enumerate(bars):
        width = bar.get_width()
        label_x = width + 0.01
        if label_x > 1.0:
            label_x = width - 0.02
            text_color = 'white'
        else:
            text_color = 'black'

        ax2.text(label_x, bar.get_y() + bar.get_height()/2,
                 f'{width:.3f}', ha='left', va='center', fontsize=10,
                 fontweight='bold', color=text_color)
    fig.tight_layout()
    # Save as TIFF format
    output_path = os.path.join(output_dir, '147_Algorithm_Comparison_Combined.tiff')
    plt.savefig(output_path, dpi=600, bbox_inches='tight')
    print(f"✅ Algorithm model comparison combined plot saved as: {output_path}")
    plt.show()

except Exception as e:
```

```
    print(f"Error generating combined plot: {e}")

# =================== Generate Feature Importance Visualization ====================
print("\n" + "=" * 80)
print("Generating feature importance visualization...")
print("=" * 80)

try:
    # 1. Overall feature importance plot
    print("\n1. Generating overall feature importance plot...")
    plt.figure(figsize=(12, 8))

    features = list(overall_feature_importance_sorted.keys())
    importance_values = list(overall_feature_importance_sorted.values())

    colors = plt.cm.viridis(np.linspace(0.3, 0.9, len(features)))
    bars = plt.barh(range(len(features)), importance_values, color=colors)

    plt.yticks(range(len(features)), features, fontsize=10)
    plt.xlabel('Normalized Importance Score', fontsize=12)
    plt.title('(C) ',
              size=14, fontweight='bold', pad=15)
    plt.xlim(0, max(importance_values) * 1.15)

    # Add value labels
    for i, bar in enumerate(bars):
        width = bar.get_width()
        plt.text(width + 0.005, bar.get_y() + bar.get_height()/2,
                 f'{width:.4f}', ha='left', va='center', fontsize=10)

    plt.tight_layout()
    output_path = os.path.join(feature_importance_dir, '147_Overall_Feature_Importance.tiff')
    plt.savefig(output_path, dpi=600, bbox_inches='tight')
    print(f"    ☑ Saved as: {output_path}")
    plt.show()

except Exception as e:
    print(f"Error generating feature importance visualization: {e}")

# =================== Print Summary Results ===================
print("\n" + "=" * 80)
print("Performance Comparison Summary")
print("=" * 80)
```

```
print(results_df.round(3))

# Find the best model for each metric
print("\nBest model for each metric:")
for metric in ['Accuracy', 'Precision', 'Recall', 'F1 Score', 'AUC-ROC', 'Balanced Accuracy',
'Matthews Correlation Coefficient', 'Cross-validation Accuracy']:
    if metric in results_df.columns:
        best_model = results_df[metric].idxmax()
        best_score = results_df[metric].max()
        print(f"{metric}: {best_model} ({best_score:.3f})")

print("\n" + "=" * 80)
print("Feature Importance Summary")
print("=" * 80)

print("\nOverall feature importance ranking:")
for i, (feature, score) in enumerate(overall_feature_importance_sorted.items(), 1):
    print(f"{i}. {feature}: {score:.4f}")

print("\nTop 3 features for each model:")
for idx, row in feature_importance_df.iterrows():
    print(f"\n{row['Model']}:")
    for feat, val in zip(row['Top_3_Features'], row['Top_3_Values']):
        print(f"    - {feat}: {val:.4f}")

# Find the top 3 most important features
top_3_overall = list(overall_feature_importance_sorted.items())[:3]
print(f"\n    Top 3 most important features:")
for i, (feature, score) in enumerate(top_3_overall, 1):
    print(f"{i}. {feature} (importance score: {score:.4f})")

# Create detailed comparison table
detailed_comparison = results_df.round(4)
detailed_comparison['Overall_Rank'] =
detailed_comparison.mean(axis=1).rank(ascending=False).astype(int)
detailed_comparison = detailed_comparison.sort_values('Overall_Rank')

detailed_csv_path = os.path.join(output_dir, '147_Detailed_Performance_Comparison.csv')
detailed_comparison.to_csv(detailed_csv_path, encoding='utf-8-sig')
print(f"\n✅ Detailed performance comparison table saved as: {detailed_csv_path}")

best_overall = detailed_comparison.index[0]
print(f"\n    Recommended best overall model: {best_overall}")
print(f"Overall ranking score: {detailed_comparison.loc[best_overall].mean():.4f}")
```

```
# =================== DeLong Test Final Summary ===================
print("\n" + "=" * 80)
print("DeLong's Test Final Summary")
print("=" * 80)

print(f"\nTotal models compared: {len(model_names_with_probs)}")
print(f"Total pairwise comparisons: {comparison_count}")
print(f"\nSignificance levels:")
print(f"  p < 0.05: {significant_count_05} comparisons ({significant_count_05/comparison_count*100:.1f}%)")
print(f"  p < 0.01: {significant_count_01} comparisons ({significant_count_01/comparison_count*100:.1f}%)")
print(f"  p < 0.001: {significant_count_001} comparisons ({significant_count_001/comparison_count*100:.1f}%)")

print(f"\nBest model by AUC: {best_auc_model} (AUC = {best_auc:.4f})")
if best_auc_model != best_overall:
    print(f"Note: Best model by overall performance is {best_overall}")

print(f"\nModels not significantly different from {best_auc_model} (p ⩾ 0.05):")
for idx, row in best_comparisons.iterrows():
    other_model = row['Model 2'] if row['Model 1'] == best_auc_model else row['Model 1']
    if row['P-Value'] >= 0.05:
        print(f"    - {other_model} (p = {row['P-Value']:.4f})")

# =================== Final Summary ===================
print("\n" + "=" * 80)
print("Analysis completed successfully!")
print("=" * 80)

print(f"\n    All files generated in '{output_dir}' directory:")

print("\n1. Algorithm model comparison combined plot:")
print(f"      - 147_Algorithm_Comparison_Combined.tiff - Algorithm model comparison combined plot (for publication)")

print("\n2. Performance results files:")
print(f"      - 147_Performance_Results.csv - Performance metrics")
print(f"      - 147_Detailed_Performance_Comparison.csv - Detailed performance comparison")

print(f"\n3. Feature importance analysis files (in 'feature_importance' subdirectory):")
print(f"      - 147_Feature_Importance_Summary.csv - Feature importance for each model")
print(f"      - 147_Overall_Feature_Importance.csv - Overall feature importance")
```

```
print(f"      - 147_Overall_Feature_Importance.tiff - Overall feature importance plot")

print(f"\n4. DeLong's Test files (in 'delong_test' subdirectory):")
print(f"      - 147_Delong_Test_Results.csv - DeLong's test pairwise comparison results")
print(f"      - 147_Delong_Test_Heatmap.tiff - DeLong's test P-value heatmap")

print("\n" + "=" * 80)
print("Key Findings:")
print("=" * 80)
print(f"1. Best performing model: {best_overall}")
print(f"2. Most important features: {', '.join([f[0] for f in top_3_overall])}")
print(f"3. Total number of models evaluated: {len(fixed_optimized_models) + 1} (including ensemble model)")
print(f"4. All feature importance values have been properly normalized, ranging from 0-1")
print(f"5. All charts have been saved in TIFF format, suitable for publication")
print(f"6. DeLong's test completed with {comparison_count} pairwise comparisons")
print(f"7. {significant_count_05} significant differences found (p<0.05) among model AUCs")
print("=" * 80)
```
