# Supplementary material for: Research on the severity of symptoms in children with ASD based on integrated machine learning and structural equation modeling: age-specific predictive features and mediation effect path analysis
Source: Front Pediatr. 2026 Jun 17;14:1841816. doi: 10.3389/fped.2026.1841816 (PMC13318893; doi:10.3389/fped.2026.1841816)
Supplement: Supplementary file 12 [file Datasheet3.pdf]

SEM code for the total sample

```
# Load packages
library(tidyverse)
library(lavaan)
library(semPlot)
library(readxl)

# Read data
data <- read_excel('C:/Correlation between different first diagnosis ages and developmental levels/Research data/total sample.xlsx')

# Check data
str(data)
head(data)

# Construct structural equation model - optimized version
model <- '
    # Measurement model - fix one loading to 1 to set the scale
    Developmental =~ 1*adaptive_behavior + gross_motor + fine_motor + language + personal_social
    Physical =~ 1*HAZ + WAZ

    # Structural model - add path labels for effect decomposition
    Developmental ~ a1 * age
    Physical ~ a2 * age
    CARS ~ b1 * Developmental + b2 * Physical + cp * age

    # Define indirect and total effects
    indirect1 := a1 * b1
    indirect2 := a2 * b2
    total_indirect := indirect1 + indirect2
    direct := cp
    total := cp + indirect1 + indirect2

   # Add reasonable residual correlations
    # Correlations within developmental domains
    adaptive_behavior ~~ fine_motor + personal_social
    gross_motor ~~ fine_motor
    language ~~ personal_social
    gross_motor ~~ personal_social
    fine_motor ~~ personal_social
    # Correlations within physical indicators
    HAZ ~~ WAZ
```

```
    WAZ ~~ BAZ
'

# Fit the model
fit <- sem(model,
           data = data,
           estimator = "ML",
           missing = "ML",
           se = "bootstrap",    # Use bootstrap standard errors for robustness
           bootstrap = 5000)    # Number of bootstrap iterations

# View model results
summary(fit,
        standardized = TRUE,
        fit.measures = TRUE,
        rsquare = TRUE,
        ci = TRUE)    # Display confidence intervals
# Draw path diagram
semPaths(fit,
         what = "std",
         layout = "tree",    # Use tree layout for clarity
         fade = FALSE,
         residuals = TRUE,    # Display residuals for diagnostics
         nCharNodes = 0,
         edge.label.cex = 0.9,
         sizeMan = 8,
         sizeLat = 10,
         edge.color = "black",
         nodeLabels = c("age", "HAZ", "WAZ", "adaptive_behavior", "gross_motor",
                        "fine_motor", "language", "personal_social", "CARS",
                        "Developmental", "Physical"),
         label.cex = 1.1,
         curvePivot = TRUE)
```

SEM code for the low-age group

```
# Load packages
library(tidyverse)
library(lavaan)
library(semPlot)
library(readxl)

# Read data
data <- read_excel('C:/Correlation between different first diagnosis ages and developmental levels/Research data/low-age group.xlsx')

# Check data
str(data)
head(data)

# Construct structural equation model - optimized version
model <- '
    # Measurement model - fix one loading to 1 to set the scale
    Developmental =~ 1*adaptive_behavior + gross_motor + fine_motor + language + personal_social
    Physical =~ 1*HAZ + WAZ

    # Structural model - add path labels for effect decomposition
    Developmental ~ a1 * age
    Physical ~ a2 * age
    CARS ~ b1 * Developmental + b2 * Physical + cp * age

    # Define indirect and total effects
    indirect1 := a1 * b1
    indirect2 := a2 * b2
    total_indirect := indirect1 + indirect2
    direct := cp
    total := cp + indirect1 + indirect2


    # Add reasonable residual correlations
    # Correlations within developmental domains
    adaptive_behavior ~~ personal_social + gross_motor + fine_motor
    gross_motor ~~ fine_motor
    language ~~ personal_social
    gross_motor ~~ personal_social
    fine_motor ~~ personal_social
    # Correlations within physical indicators
```

```
    HAZ ~~ WAZ
    WAZ ~~ BAZ
'

# Fit the model
fit <- sem(model,
           data = data,
           estimator = "ML",
           missing = "ML",
           se = "bootstrap",   # Use bootstrap standard errors for robustness
           bootstrap = 5000)   # Number of bootstrap iterations

# View model results
summary(fit,
        standardized = TRUE,
        fit.measures = TRUE,
        rsquare = TRUE,
        ci = TRUE)   # Display confidence intervals

# Draw path diagram
semPaths(fit,
         what = "std",
         layout = "tree",   # Use tree layout for clarity
         fade = FALSE,
         residuals = TRUE,   # Display residuals for diagnostics
         nCharNodes = 0,
         edge.label.cex = 0.9,
         sizeMan = 8,
         sizeLat = 10,
         edge.color = "black",
         nodeLabels = c("age", "HAZ", "WAZ", "adaptive_behavior", "gross_motor",
                        "fine_motor", "language", "personal_social", "CARS",
                        "Developmental", "Physical"),
         label.cex = 1.1,
         curvePivot = TRUE)
```

SEM code for the high-age group

```
# Load packages
library(tidyverse)
library(lavaan)
library(semPlot)
library(readxl)

# Read data
data <- read_excel('C:/Correlation between different first diagnosis ages and developmental levels/Research data/high-age group.xlsx')

# Check data
str(data)
head(data)

# Construct structural equation model - optimized version
model <- '
    # Measurement model - fix one loading to 1 to set the scale
    Developmental =~ 1*adaptive_behavior + gross_motor + fine_motor + language + personal_social
    Physical =~ 1*HAZ + WAZ

    # Structural model - add path labels for effect decomposition
    Developmental ~ a1 * age
    Physical ~ a2 * age
    CARS ~ b1 * Developmental + b2 * Physical + cp * age

    # Define indirect and total effects
    indirect1 := a1 * b1
    indirect2 := a2 * b2
    total_indirect := indirect1 + indirect2
    direct := cp
    total := cp + indirect1 + indirect2


    # Add reasonable residual correlations
    # Correlations within developmental domains
    adaptive_behavior ~~ personal_social + gross_motor + fine_motor
    gross_motor ~~ fine_motor
    language ~~ personal_social
    gross_motor ~~ personal_social
    fine_motor ~~ personal_social
```

```
	# Correlations within physical indicators
	HAZ ~~ WAZ
	WAZ ~~ BAZ
'

# Fit the model
fit <- sem(model,
           data = data,
           estimator = "ML",
           missing = "ML",
           se = "bootstrap",    # Use bootstrap standard errors for robustness
           bootstrap = 5000)    # Number of bootstrap iterations

# View model results
summary(fit,
        standardized = TRUE,
        fit.measures = TRUE,
        rsquare = TRUE,
        ci = TRUE)    # Display confidence intervals

# Draw path diagram
semPaths(fit,
         what = "std",
         layout = "tree",    # Use tree layout for clarity
         fade = FALSE,
         residuals = TRUE,    # Display residuals for diagnostics
         nCharNodes = 0,
         edge.label.cex = 0.9,
         sizeMan = 8,
         sizeLat = 10,
         edge.color = "black",
         nodeLabels = c("age", "HAZ", "WAZ", "BAZ", "adaptive_behavior", "gross_motor",
                        "fine_motor", "language", "personal_social", "CARS",
                        "Developmental", "Physical"),
         label.cex = 1.1,
         curvePivot = TRUE)
```
