# Supplementary material for: Research on the severity of symptoms in children with ASD based on integrated machine learning and structural equation modeling: age-specific predictive features and mediation effect path analysis
Source: Front Pediatr. 2026 Jun 17;14:1841816. doi: 10.3389/fped.2026.1841816 (PMC13318893; doi:10.3389/fped.2026.1841816)
Supplement: Supplementary file 13 [file Datasheet4.pdf]

## Table 6. Path coefficients of the structural equation model for the high-age group

| Variables | B | 95%Confidence Interval | | Z | *P* | β |
|---|---|---|---|---|---|---|
| | | Lower | Upper | | | |
| **measurement model** | | | | | | |
| adaptive behavior→developmental level | 1.000 | 1.000 | 1.000 | | | 0.678 |
| gross motor→developmental level | 0.701 | 0.468 | 0.964 | 5.591 | *<0.001* | 0.481 |
| fine motor→developmental level | 1.148 | 0.972 | 1.370 | 12.216 | *<0.001* | 0.690 |
| language→developmental level | 1.253 | 0.812 | 2.070 | 3.670 | *<0.001* | 0.844 |
| personal-social→developmental level | 1.151 | 0.809 | 1.758 | 4.763 | *<0.001* | 0.787 |
| HAZ→physical development | 1.000 | 1.000 | 1.000 | | | 0.805 |
| WAZ→physical development | 0.716 | 0.572 | 0.860 | 7.202 | *<0.001* | 0.707 |
| **structural model** | | | | | | |
| age at first diagnosis→developmental level | -1.990 | -5.197 | 1.505 | -1.203 | *0.229* | -0.113 |
| age at first diagnosis→physical development | -0.422 | -1.025 | 0.093 | -1.430 | *0.153* | -0.161 |
| developmental level→CARS scores | -0.406 | -0.578 | -0.286 | -5.575 | *<0.001* | -0.443 |
| physical development→CARS scores | -0.249 | -1.030 | 0.846 | -0.547 | *0.585* | -0.041 |
| age at first diagnosis→CARS scores | -0.724 | -2.181 | 0.980 | -0.885 | *0.376* | -0.045 |
| **mediating effect** | | | | | | |
| indirect effect 1 | 0.809 | -0.602 | 2.065 | 1.201 | *0.230* | 0.050 |
| indirect effect 2 | 0.105 | -0.397 | 0.521 | 0.473 | *0.636* | 0.007 |
| total indirect effect | 0.914 | -0.580 | 2.154 | 1.325 | *0.185* | 0.057 |
| direct effect | -0.724 | -2.181 | 0.980 | -0.884 | *0.376* | -0.045 |
| total effect | 0.190 | -1.696 | 2.181 | 0.201 | *0.841* | 0.012 |

Notes:indirect effect 1=age at first diagnosis→developmental level→CARS scores,indirect effect 2=age at first diagnosis→physical development→CARS scores,total indirect effect=indirect effect 1+ indirect effect 2,direct effect=age at first diagnosis→CARS scores,total effect=total indirect effect+direct effect. B: unstandardized regression coefficient; β : standardized regression coefficient; t: t-value; p: p-value
